# Comparison of complications and bowel function among different reconstruction techniques after low anterior resection for rectal cancer: a systematic review and network meta-analysis

**DOI:** 10.1186/s12957-023-02977-z

**Published:** 2023-03-10

**Authors:** Huabing Liu, Ming Xiong, Yu Zeng, Yabo Shi, Zhihui Pei, Chuanwen Liao

**Affiliations:** 1 Medical College, Nanchang University, Nanchang, 330006 China; 2 Department of Gastrointestinal Surgery, Jiangxi Provincial People’s Hospital, The First Affiliated Hospital of Nanchang Medical College, 152 Aiguo Road, Nanchang, 330006 China

**Keywords:** Rectal cancer, Colon J-pouch, Straight colorectal anastomosis, Transverse coloplast, Side-to-end anastomosis, Network meta-analysis

## Abstract

**Background:**

Anastomosis for gastrointestinal reconstruction has been contentious after low anterior resection of rectal cancer for the past 30 years. Despite the abundance of randomized controlled trials (RCTs) on colon J-pouch (CJP), straight colorectal anastomosis (SCA), transverse coloplast (TCP), and side-to-end anastomosis (SEA), most studies are small and lack reliable clinical evidence. We conducted a systematic review and network meta-analysis to evaluate the effects of the four anastomoses on postoperative complications, bowel function, and quality of life in rectal cancer.

**Methods:**

We assessed the safety and efficacy of CJP, SCA, TCP, and SEA in adult patients with rectal cancer after surgery by searching the Cochrane Library, Embase, and PubMed databases to collect RCTs from the date of establishment to May 20, 2022. Anastomotic leakage and defecation frequency were the main outcome indicators. We pooled data through a random effects model in a Bayesian framework and assessed model inconsistency using the deviance information criterion (DIC) and node-splitting method and inter-study heterogeneity using the I-squared statistics (*I*^*2*^). The interventions were ranked according to the surface under the cumulative ranking curve (SUCRA) to compare each outcome indicator.

**Results:**

Of the 474 studies initially evaluated, 29 were eligible RCTs comprising 2631 patients. Among the four anastomoses, the SEA group had the lowest incidence of anastomotic leakage, ranking first (SUCRA_SEA_ = 0.982), followed by the CJP group (SUCRA_CJP_ = 0.628). The defecation frequency in the SEA group was comparable to those in the CJP and TCP groups at 3, 6, 12, and 24 months postoperatively. In comparison, the defecation frequency in the SCA group 12 months after surgery all ranked fourth. No statistically significant differences were found among the four anastomoses in terms of anastomotic stricture, reoperation, postoperative mortality within 30 days, fecal urgency, incomplete defecation, use of antidiarrheal medication, or quality of life.

**Conclusions:**

This study demonstrated that SEA had the lowest risk of complications, comparable bowel function, and quality of life compared to the CJP and TCP, but further research is required to determine its long-term consequences. Furthermore, we should be aware that SCA is associated with a high defecation frequency.

**Supplementary Information:**

The online version contains supplementary material available at 10.1186/s12957-023-02977-z.

## Background

Colorectal cancer is the third most common cancer globally and the second largest cause of cancer fatalities, with rectal cancer accounting for at least one-third of all cancer fatalities [1]. Transabdominal low anterior resection (LAR) combined with total mesorectal excision (TME) is the standard approach for treating mid-low rectal cancer. However, anal sphincter and parasympathetic nerve damage and loss of rectal storage function result from gastrointestinal tract reconstruction after TME. Unfortunately, statistics show that approximately 50% of patients with rectal cancer experience postoperative complications, and up to 90% of patients have “low anterior resection syndrome” [2–5]. These complications and bowel dysfunction may affect a patient’s quality of life and long-term prognosis [6–9].

Conventional straight colorectal anastomosis is associated with numerous complications and is prone to severe bowel dysfunction [10–12]. To improve the intestinal function and quality of life of patients, Lazorthes et al. [13] and Parc et al. [14] proposed colon J-pouch (CJP) in 1986. Expanding the volume of the “new rectum” significantly improves bowel function after surgery. However, anatomical factors, including pelvic stenosis, mesenteric hypertrophy, and shortage of colon, limited CJP application; therefore, Z’Graggen et al. [15] introduced transverse coloplast (TCP) in 1999. Early research indicates that TCP was highly operational and had a similar impact on bowel function as CJP but also carried a significant risk of complications. Baker [16] first proposed the side-to-end anastomosis (SEA) technique in 1950, but its advantages were insignificant. It has reemerged in the spotlight in recent years as an alternative strategy to enhance postoperative bowel function.

Previous Cochrane systematic reviews and meta-analyses have concluded that the four anastomoses have similar complication rates. However, CJP is better than straight colorectal anastomosis (SCA) in bowel function, with no difference from SEA or TCP [17, 18]. The data they aggregated were the findings of a certain period rather than a specific point in time because of the few studies; therefore, the conclusions may be skewed. There is no consensus regarding the safest and most efficient anastomosis approach, despite an increase in the number of pertinent randomized controlled trials (RCTs) published in recent years. A network meta-analysis of postoperative complications and bowel function at 3, 6, 12, and 24 months after surgery in patients with rectal cancer will assist in summarizing the various impacts of the four anastomoses from direct and indirect comparisons by combining some of the limited trials in these systematic reviews and some recently published RCTs. This study aimed to provide more robust and comprehensive evidence for determining optimal anastomosis in clinical practice.

## Methods

### Protocol

The protocol for this study is registered with PROSPERO (CRD42022332911, https://www.crd.york.ac.uk/prospero/), and we have reported the results according to the Preferred Reporting Items for Systematic Reviews and Meta-analyses (PRISMA) extended statement [19].

### Search strategy

We collected RCTs from database establishment to May 20, 2022, by searching the Cochrane Library, Embase, and PubMed databases (see Supplementary Table 2, Supplementary File 1 for the detailed search strategy). Two authors (MX and YZ) screened the search results. After reading the relevant literature abstracts, we manually searched the corresponding full texts and the references of the obtained articles to avoid missing important articles.

### Inclusion and exclusion criteria

Inclusion criteria were as follows: (1) the study participants were adults with rectal cancer who underwent surgical treatment, (2) at least two of the anastomosis techniques (CJP, SCA, TCP, SEA) were included in the study, (3) at least one of the primary outcome indicators (anastomotic leakage and defecation frequency) was included, and (4) the research type was English RCTs. Exclusion criteria were as follows: (1) non-randomized controlled trials, including reviews, retrospective studies, commentaries, and meta-analyses; (2) lack of available data or outcomes; and (3) duplicate publication of content. Two authors (MX and YZ) independently reviewed the entire text in accordance with the inclusion and exclusion criteria, consulted a third author (CWL) in case of disagreement, and decided on the inclusion of eligible studies at a conference.

### Data extraction and processing

Two authors (MX and YZ) independently extracted the following data: (1) anastomotic leakage, (2) defecation frequency, (3) anastomotic stricture, (4) reoperation, (5) postoperative mortality within 30 days, (6) fecal urgency, (7) incomplete defecation, (8) use of antidiarrheal medication, and (9) quality of life. We recorded the results of bowel function outcomes at 3, 6, 12, and 24 months following stoma retraction (or without stoma surgery). We considered the most common and concerning anastomotic leakage and defecation frequency as the primary outcome indicators, and the rest were secondary outcome indicators. Anastomotic leakage is defined as a significant crack at the edge of the anastomosis, leakage of bowel contents seen in the pelvis on imaging or endoscopy, or purulent discharge from the pelvic drainage tube. The defecation frequency was determined based on the patient-described average number of daily bowel movements.

### Quality assessment

Two authors (YBS and ZHP) independently assessed the risk of bias for all included studies using the revised Cochrane Rob2 tool, and studies with disagreements were resolved by a third author (CWL) [20]. The following five domains were assessed separately for the included studies: randomization process, deviation from the intended interventions, missing outcome data, measurement of the outcome, and selection of the reported result. Each study’s final overall risk of bias was rated as “low,” “moderate concerns,” or “high.”

### Statistical analysis

We performed a network meta-analysis using the Bayesian framework employing gemtc and rjags packages in R4.2.0 (https://www.r-project.org/). Simultaneously, the meta package was used for pairwise analysis. Network meta-analysis results provided more accurate estimates and ranked various interventions to provide clinical recommendations compared to results from traditional pairwise analyses [21, 22]. We uniformly used random effects models as conservative estimates, generating a risk ratio (RR) or mean difference (MD) with a 95% confidence interval (CI) to represent the efficacy of each intervention. We compared the consistent and inconsistent models using the deviance information criterion (DIC) [23]. A difference of the DIC less than 5 implies that the model has good goodness of fit, and there is no global inconsistency. In addition, we assessed the local inconsistency of the model using the node-splitting method [24, 25]. If the value of *P* > 0.05, the direct comparison was considered to be in good agreement with the indirect comparison. We also evaluated the heterogeneity between studies using the I-squared statistics (*I*^*2*^) [26, 27]. The range of *I*^*2*^ values was 0–100%, where 0–49% was low heterogeneity, 50–74% moderate heterogeneity, and 75–100% high heterogeneity. By calculating the surface under the cumulative ranking curve (SUCRA), we compared and ranked the safety and efficacy of various interventions. Higher ranking grades indicated lower perioperative complication rates or better bowel function. Due to the large variation in sample sizes of the included studies, sensitivity analyses on anastomotic leakage were performed to assess the reliability of the results, which included only studies with sample sizes greater than or equal to 20 in a single arm. To assess the publication bias of studies in the network meta-analysis, we used STATA 16.0 (Stata Corporation, College Station, TX, USA) to generate a comparison-adjusted funnel plot and thus explore the impact of publication bias or other small-sample studies [28]. In the absence of publication bias, the estimates for all comparisons were symmetrically distributed around the null hypothesis.

## Results

### Study characteristics

**Fig. 1:**
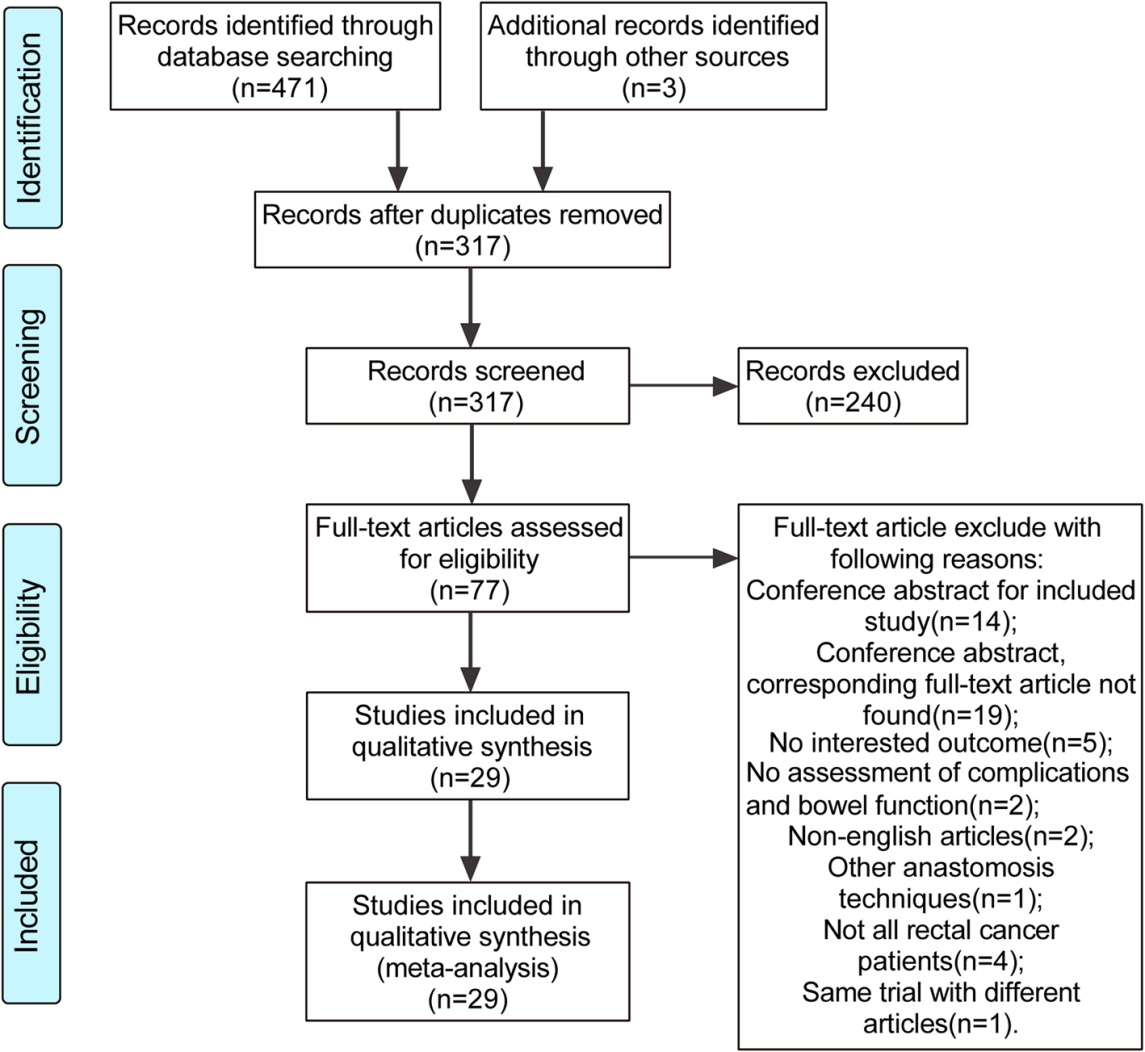
PRISMA flow diagram

We identified 471 articles by searching the database and three articles from the reference list for a total of 474 articles (Fig. 1). After eliminating duplicates and preliminary screening of titles and abstracts, we excluded irrelevant studies, and the remaining 77 articles were potentially relevant to this study. Two authors (MX and YZ) independently reviewed the full text of the relevant literature and found that 29 studies (27 trials) met the inclusion criteria, of which Ho et al. [29, 30] and Machado et al. [31, 32] were both based on the same trial and reported two different results. These 29 studies were mainly distributed in Europe, Asia, and North America, of which six [33–38] were multicenter studies, and the remaining 23 were single-center studies. The characteristics of all the included studies are shown in Supplementary Table 3 (Supplementary File 1). In all studies, 2631 patients were included. Of these, 1166 (44%) were randomly assigned to the CJP group, 671 (25%) to the SCA group, 386 (14%) to the TCP group, and 408 (17%) to the SEA group. The studies were published from 1995 to 2019, with a maximum follow-up period of 5 years. Patients in most studies underwent ileostomies or colostomies, and only a few studies had elective or no stomas. Except for Liang et al. [39], Okkabaz et al. [40], and Parc et al. [36], all studies used open surgery.

The quality assessment of the included studies is presented in Supplementary Fig. 1 and Table 4 (Supplementary File 1). Nine (31.0%) of the total included studies [32, 34, 41–47] did not specifically state how the randomization sequence was assigned, and their randomization procedures were either high-risk or had some concerns. Due to the nature of the surgery, double-blinding was not feasible; however, this had less impact on the results. Clinicians must choose the final anastomosis based on the patient’s anatomical condition. This may lead to treatments that are not pre-randomized outcomes, with four (13.8%) [45, 48–50] studies at a high risk of deviating from the expected interventions. Of the 29 studies, one (3.4%) [42] had missing outcome data, three (10.3%) [35, 37, 50] had high-risk outcome measures, and one (3.4%) [30] was at high risk for selective reporting of results.

### Results of a pairwise meta-analysis

The results of the pairwise meta-analysis of perioperative complications and bowel function are shown in Supplementary Figs. 2, 3, and 4 (Supplementary File 1). Regarding perioperative complications, compared to the CJP and SCA groups, the SEA group showed a slight decrease in the incidence of anastomotic leakage [(RR = 0.77, 95% CI 0.44–1.35) and (RR = 0.39, 95% CI 0.10–1.52)]. However, pairwise comparisons between interventions failed to show statistical differences. At 6 and 12 months postoperatively, we found that the SCA group had a substantially higher defecation frequency than the CJP group [(MD = 1.91, 95% CI 0.95–2.86) and (MD = 1.23, 95% CI 0.59–1.87)]. In terms of fecal urgency, the SCA and TCP groups had significantly more fecal urgency than the CJP group at 12 months postoperatively [(RR = 1.37, 95% CI 1.15–1.62) and (RR = 1.26, 95% CI 1.07–1.48)], respectively. Regarding the use of antidiarrheal drugs, the SCA group was significantly more frequent than the CJP group 6 months postoperatively (RR = 2.29, 95% CI 1.23–4.26). There was no discernible difference in incomplete defecation between the SCA, SEA, and CJP groups 6 and 12 months after surgery.

### Results of network meta-analysis

#### Postoperative complications

**Fig. 2:**
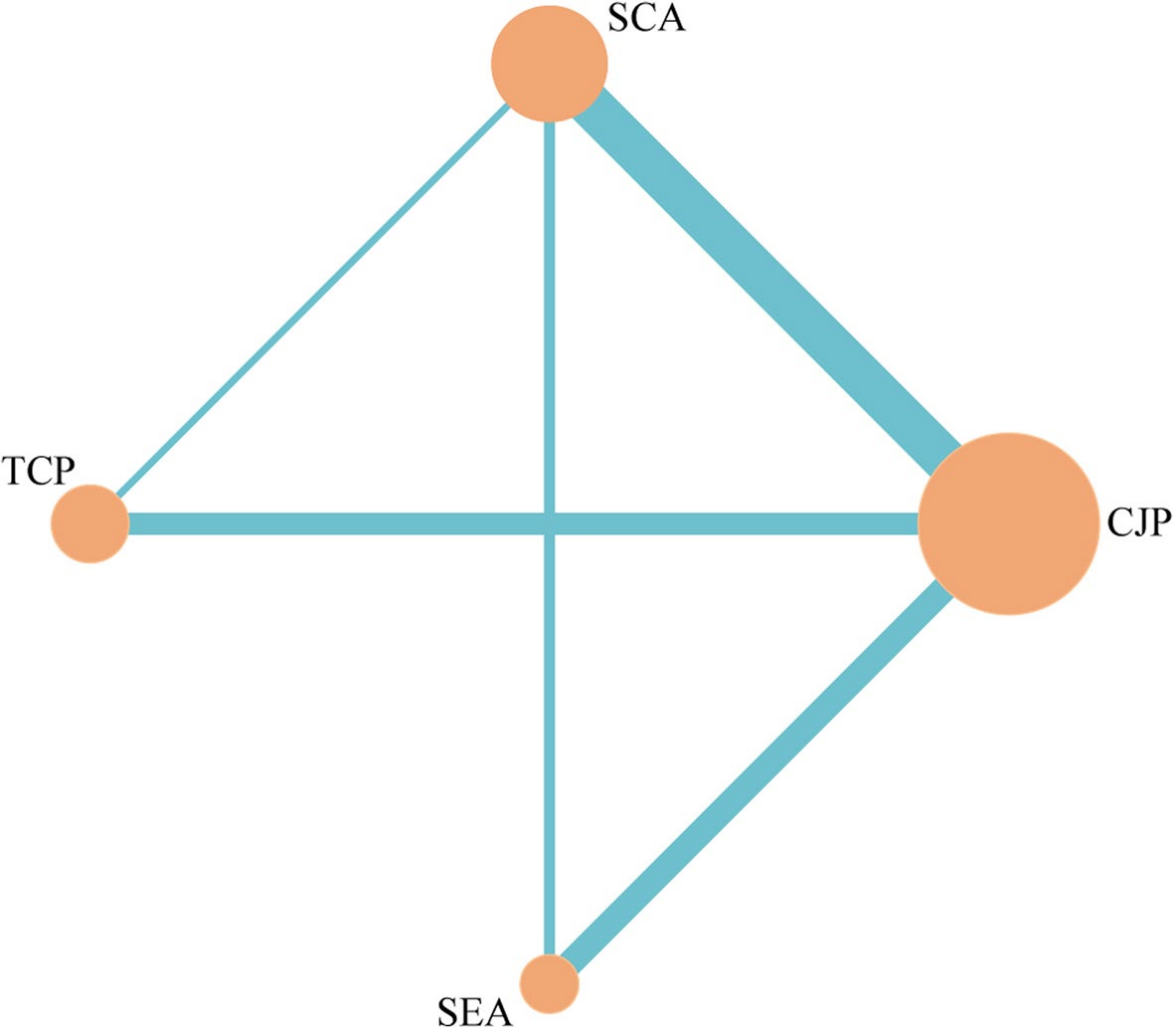
Network plot for anastomotic leakage. Circles represent interventions, and their size is proportional to the number of patients who received the corresponding intervention. Lines represent direct comparisons, and their width is proportional to the number of studies in the corresponding comparison. CJP, colon J-pouch; SCA, straight colorectal anastomosis; TCP, transverse coloplasty; SEA, side-to-end anastomosis

**Fig. 3:**
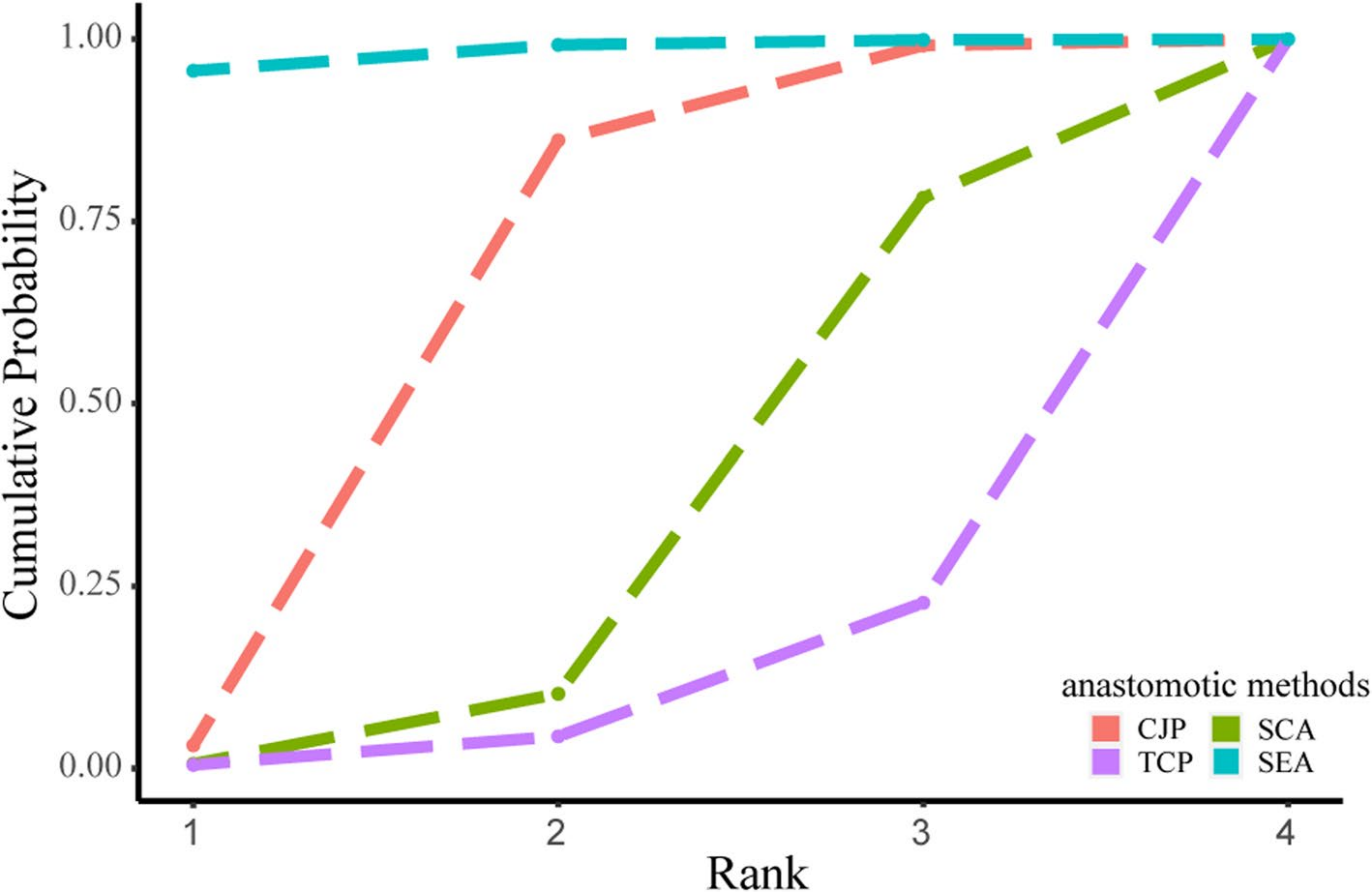
Cumulative probability rank plots for anastomotic leakage. CJP, colon J-pouch; SCA, straight colorectal anastomosis; TCP, transverse coloplasty; SEA, side-to-end anastomosis

Twenty-four studies [29, 31, 33–43, 45–47, 49–56] reported a primary safety indicator, anastomotic leakage. A network plot of the postoperative anastomotic leakage is shown in Fig. 2. The SEA group showed a considerably lower incidence of anastomotic leakage than either the SCA group or the TCP group [(RR = 0.36, 95% CI 0.14–0.81) and (RR = 0.24, 95% CI 0.06–0.74)]; however, there was no significant difference between the SEA and CJP groups. Figure 3 demonstrates that the SUCRA for the SEA group was the highest, ranking first (SUCRA_SEA_ = 0.982) and that for the CJP group was second (SUCRA_CJP_ = 0.628). These two anastomoses are the most secure. Additionally, 10 [31, 33, 34, 36, 40, 42, 43, 47, 50, 54], 13 [31, 33, 35–38, 40, 41, 43, 47, 49, 53, 55], and 14 studies [31, 33, 35, 38, 40–43, 49, 50, 52–54, 56] reported anastomotic stricture, reoperation, and postoperative mortality within 30 days, respectively. We found no differences among the four interventions. However, the SEA group ranked first for both reoperation and postoperative mortality within 30 days (SUCRA_SEA_ = 0.946 and 0.826), whereas the TCP group ranked fourth for both (SUCRA_SEA_ = 0.084 and 0.143).

#### Bowel function

##### Results at 3 months

Five [39, 43, 46, 51, 54], six [39, 43, 46, 51, 54, 55], and five studies [39, 43, 51, 54, 55] reported defecation frequency, fecal urgency, and the use of antidiarrheal medication, respectively. Like previous studies, the defecation frequency in the TCP and CJP groups ranked first (SUCRA_TCP_ = 0.727) and second (SUCRA_CJP_ = 0.720), respectively, followed by the SEA and SCA groups. However, there was no statistical difference among the groups (Table 1). There were no differences among the groups regarding fecal urgency and use of antidiarrheal medication, but the CJP group still ranked better than the SEA and SCA groups.

**Table 1:** Network league table of defecation frequency at 3 and 6 months postoperatively

CJP	**1.88 [1.07; 2.78]**	0.35 [ −1.94; 2.68]	0.04 [**−**0.80; 0.90]
**−**1.98 [**−**5.02; 1.12]	SCA	**−**1.53 [**−**4.02; 0.93]	**−1.84 [−3.03; −0.72]**
0.54 [**−**4.19; 5.27]	2.54 [**−**3.08; 8.11]	TCP	**−**0.31 [**−**2.79; 2.15]
**−**1.18 [**−**3.68; 1.34]	0.81 [**−**2.74; 4.30]	**−**1.72 [-7.04; 3.63]	SEA

The results below the diagonal in the table are 3 months postoperative and those above the diagonal are 6 months postoperative. Estimates are presented as MD with a 95% confidence interval. The statistically significant results are indicated in bold. *CJP* colon J-pouch, *SCA* straight colorectal anastomosis, *TCP* transverse coloplasty, *SEA* side-to-end anastomosis

##### Results at 6 months

Thirteen [30, 31, 36, 39, 40, 42, 43, 46, 50–52, 54, 57], eight [39, 40, 43, 46, 51, 52, 54, 55], seven [30, 31, 38, 39, 51, 52, 54], and eight studies [30, 38, 39, 43, 51, 54, 55, 57] reported defecation frequency, fecal urgency, incomplete defecation, and the use of antidiarrheal medication, respectively. The SEA, TCP, and CJP groups did not differ significantly in terms of defecation frequency, but all performed better than the SCA group (Table 1), with the second (SUCRA_SEA_ = 0.689), third (SUCRA_TCP_ = 0.556), and first (SUCRA_CJP_ = 0.722) positions, respectively. No intervention was found to be noticeably superior regarding fecal urgency, incomplete defecation, and use of antidiarrheal medication. The TCP group ranked first in fecal urgency and incomplete defecation (SUCRA_TCP_ = 0.689 and 0.603, respectively), while the CJP group ranked second in both (SUCRA_CJP_ = 0.612 and 0.548, respectively). The SEA group ranked first in the use of antidiarrheal medications (SUCRA_SEA_ = 0.718).

##### Results at 12 months

Twelve [29, 31, 33, 34, 36, 40, 43, 48, 49, 52, 54, 57], nine [34, 40, 41, 43, 46, 48, 52, 54, 57], six [29, 31, 48, 49, 52, 54], and six studies [29, 33, 43, 48, 49, 54] reported defecation frequency, fecal urgency, incomplete defecation, and the use of antidiarrheal medication, respectively. The SEA, TCP, and CJP groups did not differ significantly in terms of defecation frequency, but all performed better than the SCA group (Table 2), with the third (SUCRA_SEA_ = 0.587), second (SUCRA_TCP_ = 0.659), and first (SUCRA_CJP_ = 0.743) positions, respectively. In addition, no substantial advantage was discovered for any specific intervention of fecal urgency, incomplete defecation, and use of antidiarrheal medication; nevertheless, the SEA group ranked best for both fecal urgency and use of antidiarrheal medication (SUCRA_SEA_ = 0.773 and 0.679).

**Table 2:** Network league table of defecation frequency at 12 and 24 months postoperatively

CJP	0.54 [**−**0.37; 1.46]	0.67 [**−**0.55; 1.89]	**−**0.11 [**−**1.06; 0.86]
**−1.20 [−1.81; −0.57]**	SCA	0.13 [**−**1.09; 1.35]	**−**0.65 [**−**1.98; 0.68]
−0.06 [−0.80; 0.77]	**1.14 [0.27; 2.09]**	TCP	**−**0.78 [**−**2.33; 0.77]
−0.12 [−0.92; 0.55]	**1.08 [0.04; 1.98]**	**−**0.06 [**−**1.25; 0.91]	SEA

The results below the diagonal in the table are 12 months postoperative and those above the diagonal are 24 months postoperative. Estimates are presented as MD with a 95% confidence interval. The statistically significant results are indicated in bold. *CJP* colon J-pouch, *SCA* straight colorectal anastomosis, *TCP* transverse coloplasty, *SEA* side-to-end anastomosis

##### Results at 24 months

Four [30, 32, 34, 54] and three studies [30, 34, 54] reported defecation frequency and use of antidiarrheal medication, respectively. The four intervention groups did not differ significantly in terms of defecation frequency (Table 2); however, the SEA and CJP groups were at the first (SUCRA_SEA_ = 0.779) and second (SUCRA_CJP_ = 0.727), respectively. Similarly, there was no statistical difference in the use of antidiarrheal medication among the intervention groups, but the SEA and CJP groups were first (SUCRA_SEA_ = 0.776) and second (SUCRA_CJP_ = 0.708), respectively.

#### Quality of life

Nine studies assessed the quality of life of patients with different types of anastomoses. However, because the assessment scales and timing varied among the studies, we could not combine them for the analysis. We summarize all the results in the table below (Table 3).

**Table 3:** Summary of comparative quality of life results

Study	Intervention	Assessment scales	Assessment time	Result
Fürst 2002 [42]	CJP vs. SCA	EORTC-QLQ-C30	Preoperative, before discharge after surgery, 3/6 months after surgery	No difference between the two groups at any time
Ho 2002 [49]	CJP vs. TCP	FIQL	12 months after surgery	No difference between the two groups
Sailer 2002 [53]	CJP vs. SCA	GIQLI, EORTC-QLQ-C30, EORTC-QLQ-CR38	3/6/9/12 months after surgery	CJP group had a better quality of life at 3, 6, and 12 months after surgery
Park 2005 [44]	CJP vs. SCA	FISI, FIQL	3/12 months after surgery	CJP group had a better quality of life at 3 and 12 months after surgery
Fazio 2007 [34]	CJP vs. TCP vs. SCA	PCS, MCS	Preoperative, 4/12/24 months after surgery	No difference among the three groups at any time
Doeksen 2012 [35]	CJP vs. SEA	COREFO, SF-36, EORTC-QLQ-CR38	Preoperative, 4/12 months after surgery	No difference between the two groups at any time
Rybakov 2016 [55]	STE vs. SCA	FIQL	1/3/6 months after surgery	SEA group had a better quality of life at 1 and 3 months after surgery
Okkabaz 2017 [40]	CJP vs. SEA	SF-36, FISI, SHIM, FSFI, OABVF	Preoperative, 4/8/12 months after surgery	No difference between the two groups at any time
Parc 2019 [36]	CJP vs. SEA	SF-12, FACT-C, IIEF, FSFI, FISI	Preoperative, 6/12/24 months after surgery	Higher defecation frequency in the SEA group at 6 months after surgery; lower satisfaction with male sexual function in the CJP group

*EORTC-QLQ-C30* European Organization for Research and Treatment of Cancer-Quality of Life Questionnaire-C30, *FIQL* Fecal Incontinence Quality of Life, *GIQLI* Gastrointestinal Quality of Life Index, *EORTC-QLQ-CR38* European Organization for Research and Treatment of Cancer colorectal module-Quality of Life Questionnaire-CR38, *FISI*, Fecal Incontinence Severity Index, *FIQL* Fecal Incontinence Quality of Life, *PCS* physical component scales from the SF-36 questionnaire, *MCS* Mental component scales from the SF-36 questionnaire, *COREFO* Colorectal Functional Outcome, *SF36* The 36-Item Short Form Healthy Survey, *OABVF* Overactive Bladder Validated Form, *SF12* The 12-Item Short Form Healthy Survey, *FACT-C* Functional Assessment of Cancer Therapy-Colorectal, *SHIM* Sexual Health Inventory for Men, *FSFI* Female Sexual Function Index, *IIEF* International Index of Erectile Function. CJP, colon J-pouch, *SCA* straight colorectal anastomosis, *TCP* transverse coloplasty, *SEA* side-to-end anastomosis

## Sensitivity analysis, inconsistency, and heterogeneity

We only performed sensitivity analyses on data from anastomotic leakage because of the few studies. The DIC difference between models was reduced from 5.47 to 0.71 after removing studies with less than 20 sample sizes in a single arm, and the results were unchanged. The other outcome indicator models fit well, with the difference in DIC between the consistent and inconsistent models being less than 5. In our study, only the comparison of SCA with SEA in the fecal urgency outcome indicator at 12 months postoperatively was inconsistent (*P* = 0.02); no significant inconsistency was found in the other comparisons. Furthermore, we discovered low heterogeneity among studies on anastomotic leakage but generally high heterogeneity among studies on defecation frequency. We did not analyze it because of study limitations, but we speculated that it could be related to the variety of assessment scales and lack of objective evaluation.

## Publication bias

The effect estimates of anastomotic leakage, postoperative mortality within 30 days, and fecal urgency at 3 months postoperatively were found to be symmetric around the null hypothesis by looking at the comparison-adjusted funnel plots (see Supplementary Fig. 5B-21B, Supplementary File 1), while the other outcome indicators were asymmetric and had publication bias.

## Two-dimensional plot of primary outcomes

**Fig. 4:**
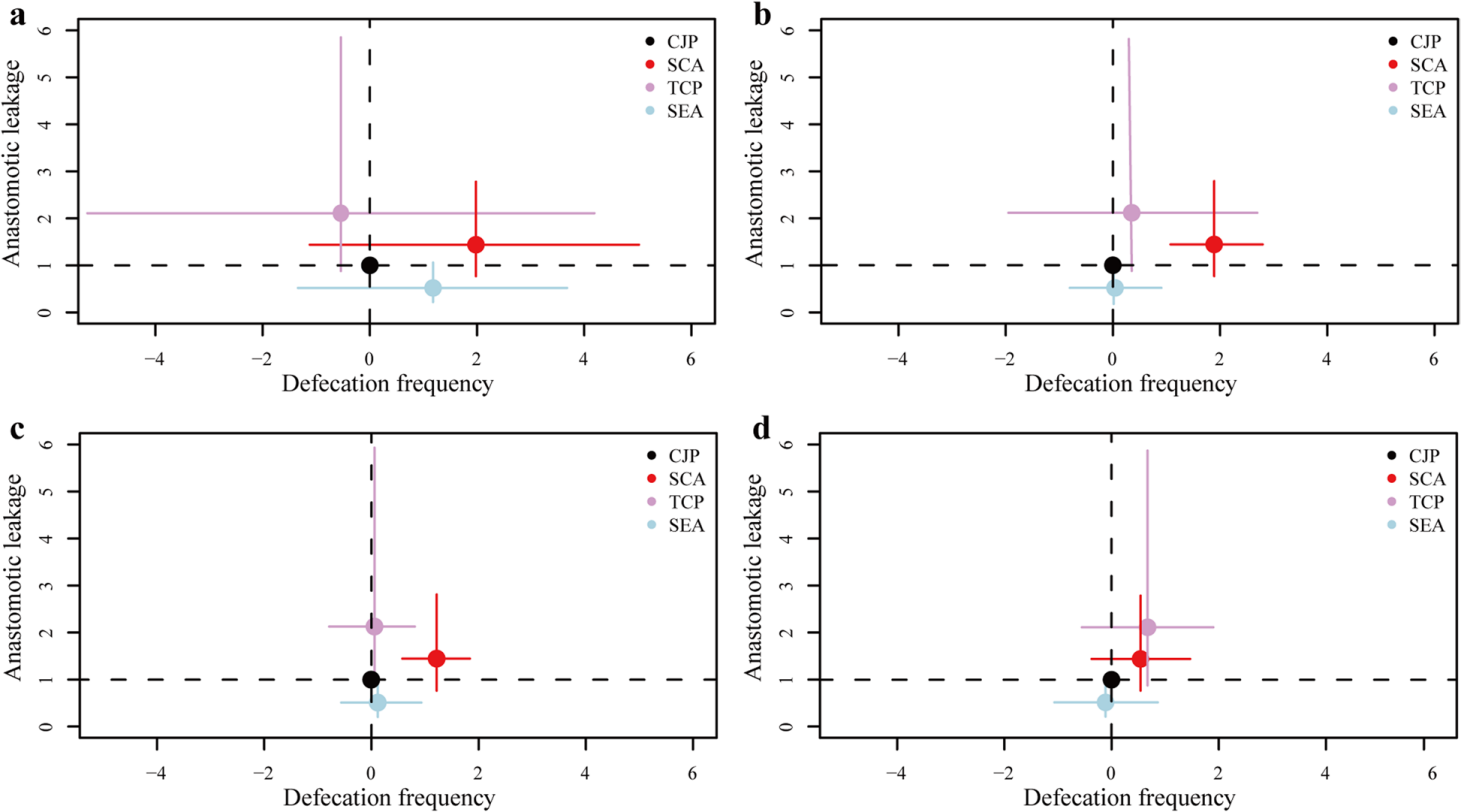
Two-dimensional plot of anastomotic leakage and defecation frequency. Using the CJP group as the control group, the *X*-axis represents the MD and 95% CI of defecation frequency, and the *Y*-axis represents the RR and 95% CI of anastomotic leakage. **a** 3 months after surgery, **b** 6 months after surgery, **c** 12 months after surgery, and **d** 24 months after surgery. CJP, colon J-pouch; SCA, straight colorectal anastomosis; TCP, transverse coloplasty; SEA, side-to-end anastomosis

The two-dimensional plot of anastomotic leakage and defecation frequency estimates revealed that SEA had the lowest probability of causing anastomotic leakage and that CJP was generally better in defecation frequency. With time, the defecation frequency of the four anastomoses tended to stabilize. The defecation frequency in the SCA group was greater at all follow-up periods; the SEA group’s frequency was the same as the CJP group’s frequency starting 6 months postoperatively, and the TCP group’s frequency fluctuated around the CJP group’s level (Fig. 4).

## Discussion

In our study analysis, SEA was the best anastomosis based on safety to avoid anastomotic leakage, CJP being the next best. In recent years, surgeons have favored SEA because of its low complication rate, good bowel function, and high operability. Two recent meta-analyses of SEA versus CJP found that the SEA group had a lower incidence of anastomotic leakage than the CJP group, although the difference was not statistically significant [58, 59]. This could be because SEA avoids requiring a lateral anastomosis at the distal colon and excessive bowel freeing, which ensures an abundant blood supply and lower anastomotic tension. Notably, anastomotic leakage can result in severe abdominal infections, pelvic abscesses, sepsis, etc., which can cause reoperations, prolonged hospital stays, cancer recurrence, and lower patient survival rates [60–63]. Due to the advantages of SEA in reducing the risk of anastomotic leakage, our findings also show that SEA outperforms other anastomotic techniques for reducing both reoperation and postoperative mortality within 30 days. SCA is the simplest and earliest form of anastomosis and is now widely accepted to have a high incidence of anastomotic leakage [64–67], as confirmed in our study. In addition, similar to the results of the previous studies by Pimentel et al. [43] and Stratilatovas et al. [45], we found the highest incidence of postoperative complications (anastomotic leakage, reoperation, and postoperative mortality within 30 days) in the TCP group. Because the coloplasty site is proximal to the colorectal anastomosis (4 cm), perfusion of the anterior rectal wall is easily compromised, resulting in all leaks after TCP is located in the anterior part of the colorectal anastomosis (below the coloplasty site) [49]. Despite the advantages of TCP, including fewer surgical restrictions and better functional bowel outcomes, serious postoperative complications have hampered its development. Factors, including neoadjuvant radiotherapy, anastomotic height, and tumor stage may negatively affect anastomotic leakage and bowel function [68–71]. Unfortunately, data from different studies were inconsistent, and we could not obtain detailed data for further analysis.

Postoperative bowel function is an important indicator of clinical efficacy. According to this meta-analysis, there was no statistically significant difference in the defecation frequency among the groups at 3 and 24 months postoperatively, with the former possibly related to high rectal sensitivity and poor adaptation in the early postoperative period and the latter with adaptation having reached equilibrium over time. At 6 and 12 months postoperatively, there was no statistically significant difference in the defecation frequency among the CJP, TCP, and SEA groups. However, all were significantly lower than that of the SCA group, with the CJP group being first. The J-shaped pouch of the CJP and the lateral limb of the SEA, as well as coloplasty, improved fecal function to some extent after increasing the volume of the “new rectum” and decreasing peristaltic waves. In a prospective RCT by Okkabaz et al. [40], a 1-year postoperative follow-up found that the number of daytime and nighttime bowel movements was essentially the same in the CJP and SEA groups, and the difference in the fecal incontinence severity index (FISI) was not statistically significant. These results are in agreement with those of Ahmed et al. [72] and Markovic et. [73] RCTs. However, a recent RCT found that SEA and CJP were not equally effective in improving postoperative bowel function [74]. Sixty patients with rectal cancer were randomly assigned to the CJP, SCA, and SEA groups. Follow-up 6 months after surgery, revealed that 70% of patients in the CJP group had normal bowel frequency. In contrast, the number of patients with normal defecation frequency was significantly lower in the other two groups (10%, 19%, *P* < 0.001). However, the study had a small sample size, short follow-up time, high missed follow-up rate, and variations in the proportion of radiotherapy among the groups, which somewhat diminished the credibility of the findings.

Regarding fecal urgency, incomplete defecation, and the use of antidiarrheal medication, we did not find a consistent advantage for any of the anastomoses. Early studies found that CJP caused incomplete defecation, but after years of clinical practice, surgeons showed that a 5–6-cm J-shaped pouch has the same defecation frequency as an 8–10-cm J-shaped pouch and significantly reduces stool retention and the use of laxatives and enemas [75, 76]. Most of the studies in this trial used smaller J-shaped pouches, the volume of which did not differ significantly from a 4–6 cm SEA’s lateral limb. Although we only pooled data on incomplete defecation at 6 and 12 months after surgery, it is sufficient to show that smaller J-shaped pouches have little effect. In a large RCT in Switzerland, 112 patients were randomly assigned to each intervention group (CJP, SEA, SCA), and no differences in composite evacuation and incontinence scores were found after 24 months of follow-up [77]. Brown et al. [17] conducted a systematic review of gastrointestinal reconstruction modalities after low anterior resection for rectal cancer and found that CJP improved bowel function for up to 2 years. Hüttner et al. [18] conducted a meta-analysis of bowel function outcomes and concluded that CJP, TCP, and SEA had better functional outcomes than SCA in the first postoperative year. However, their pooled data were divided into three stages: early (8 months), intermediate (8–18 months), and late (>18 months), and their conclusions may be uncertain. As a result, we addressed this shortcoming by statistically analyzing bowel function results at 3, 6, 12, and 24 months after surgery, respectively. From the results of our network meta-analysis, bowel function in CJP, TCP, and SEA is comparable and can be improved for 1–2 years compared with that of SCA. However, it is still difficult to definitively say which CJP and SEA is superior or inferior in terms of bowel function. Bowel function outcomes may be comparable with similar anatomical structures. Whether SEA can provide the same or better bowel function outcome as CJP in patients with rectal cancer deserves further validation through high-quality studies.

One of the primary goals of evolving rectal cancer treatment is improving the patient’s quality of life. We discovered that severe postoperative complications and bowel dysfunction do not positively correlate with the quality of life. Most patients have a higher quality of life than expected from their bowel function outcomes. Table 3 shows that five of the nine studies included did not find a statistical difference among the anastomoses. This may be because most patients with rectal cancer have become accustomed to living with bowel dysfunction before surgery and are not as severely impacted as those who do not. A multicenter prospective study assessed the quality of life and bowel function in the CJP (190 patients) and SCA groups (189 patients) 24 months after surgery and found that postoperative scores on each questionnaire were lower than baseline in both groups. However, there were no significant differences in either group [78]. Another multicenter, randomized, phase III trial (SAKK 40/04) found clinically relevant short-term deterioration in the trial outcome index (primary quality of life endpoint) in the SCA and SEA groups 6 months after surgery [79]. In contrast, scores in the CJP group remained relatively stable throughout the observation period. In addition, at 12 months, there was a significant difference in colorectal cancer symptom (secondary quality of life endpoint) scores between the SCA and CJP groups (*P* = 0.007). The results of the available studies show that the four anastomoses have a similar impact on patient’s quality of life after surgery, with possible short-term quality of life benefits for patients with J-shaped pouches; however, CJP may result in poorer sexual function in male patients. Therefore, when selecting surgical procedures for patients, particularly male patients, clinicians should consider the patient’s specific situation and the surgeon’s preferences.

### Strengths, limitations, and prospects

This systematic review integrated recent RCTs to rank postoperative complications and bowel function outcomes in rectal cancer by a network meta-analysis, giving an up-to-date basis for the anastomoses selection following rectal cancer surgery. However, some limitations limited the interpretation of our findings. First, the early trials included in this network meta-analysis were primarily single-center small-sample studies with short follow-up times and the inability to be double-blinded in clinical trials, which impacted the pooled results. Second, discrepancies in bowel dysfunction definition, measurement, and assessment scales resulted in a high level of inter-study heterogeneity in the comparison. Furthermore, some researchers have exaggerated the superiority of modified anastomosis, resulting in general publication bias in bowel function studies. Third, fecal incontinence, laxative use, enema use, anorectal pressure, overall survival, and disease-free survival, all of which are closely related to the quality of life of patients with rectal cancer, were not summarized in this network meta-analysis, and the conclusions may be limited.

Future studies should first standardize the definition of various lesions. A comprehensive, universal, and well-validated international tool should be designed to facilitate communication and dissemination of research results between different regions. Second, more long-term multicenter RCTs are required to investigate the effects of different anastomoses on patient’s quality of life and sexual and urological function.

## Conclusions

Our systematic review of postoperative complications and bowel function in rectal cancer revealed that SEA has the lowest risk of complications and the best safety profile. Six months after surgery, the CJP and SEA were identical in defecation frequency, and other indicators of bowel function were similar. We also discovered that TCP is predisposed to complications, with SCA resulting in significantly more frequent postoperative bowel movements. Furthermore, the different anastomoses did not significantly impact the patient’s quality of life. However, the heterogeneity of bowel function studies undermines the credibility of the findings. In the future, more in-depth evaluations of the benefits and risks of various anastomoses in the long-term, high-quality studies are required.

## Supplementary Information

**Additional file 1: Supplementary Table 1.** Checklist of the PRISMA extension for network meta-analysis. **Supplementary Table 2.** Number of citations by each database searched. **Supplementary Table 3. **Characteristics of the 29 studies included in the network Meta-analysis. **Supplementary Fig. 1.** Risk-of-bias summary of the randomized controlled trials. **Supplementary Table 4.** Quality assessment of included randomized controlled trials. **Supplementary Table 5.** Results of global heterogeneity and local heterogeneity. **Supplementary Table 6.** Node-splitting analysis of inconsistency. **Supplementary Table 7.** Comparisons of the fitness of consistency and inconsistency models using deviance information criteria. **Supplementary Fig. 2.** Results of pairwise meta-analysis for postoperative complications. **Supplementary Fig. 3.** Results of pairwise meta-analysis for defecation frequency. **Supplementary Fig. 4.** Results of pairwise meta-analysis for bowel function. **Supplementary Table 8A.** Relative effects table for postoperative anastomotic leakage. **Supplementary Table 8B.** Rank probabilities for postoperative anastomotic leakage. **Supplementary Fig. 5B.** Comparison-adjusted funnel plot for postoperative anastomotic leakage. **Supplementary Fig. 6A.** Network plot for postoperative anastomotic stricture. **Supplementary Table 9A.** Relative effects table for postoperative anastomotic stricture. **Supplementary Table 9B.** Rank probabilities for postoperative anastomotic stricture. **Supplementary Fig. 6B.** Comparison-adjusted funnel plot for postoperative anastomotic stricture. **Supplementary Fig. 7A.** Network plot postoperative reoperation. **Supplementary Table 10A.** Relative effects table for postoperative reoperation. **Supplementary Table 10B.** Rank probabilities for postoperative reoperation. **Supplementary Fig. 7B.** Comparison-adjusted funnel plot for postoperative reoperation. **Supplementary Fig. 8A.** Network plot for postoperative mortality within 30 days. **Supplementary Table 11A.** Relative effects table for postoperative mortality within 30 days. **Supplementary Table 11B.** Rank probabilities for postoperative mortality within 30 days. **Supplementary Fig. 8B.** Comparison-adjusted funnel plot for postoperative mortality within 30 days. **Supplementary Fig. 9A.** Network plot for defecation frequency at 3 months postoperatively. **Supplementary Table 12B.** Rank probabilities for defecation frequency at 3 months postoperatively. **Supplementary Fig. 9B.** Comparison-adjusted funnel plot for defecation frequency at 3 months postoperatively. **Supplementary Fig. 10A.** Network plot for fecal urgency at 3 months postoperatively. **Supplementary Table 13A.** Relative effects table for fecal urgency at 3 months postoperatively. **Supplementary Table 13B.** Rank probabilities for fecal urgency at 3 months postoperatively. **Supplementary Fig. 10B.** Comparison-adjusted funnel plot for fecal urgency at 3 months postoperatively. **Supplementary Fig. 11A.** Network plot for use of antidiarrheal medication at 3 months postoperatively. **Supplementary Table 14A.** Relative effects table for use of antidiarrheal medication at 3 months postoperatively. **Supplementary Table 14B.** Rank probabilities for use of antidiarrheal medication at 3 months postoperatively. **Supplementary Fig. 11B.** Comparison-adjusted funnel plot for use of antidiarrheal medication at 3 months postoperatively. **Supplementary Fig. 12A.** Network plot for defecation frequency at 6 months postoperatively. **Supplementary Table 15B.** Rank probabilities for defecation frequency at 6 months postoperatively. **Supplementary Fig .12B.** Comparison-adjusted funnel plot for defecation frequency at 6 months postoperatively. **Supplementary Fig. 13A.** Network plot for fecal urgency at 6 months postoperatively. **Supplementary Table 16A.** Relative effects table for fecal urgency at 6 months postoperatively. **Supplementary Table 16B.** Rank probabilities for fecal urgency at 6 months postoperatively. **Supplementary Fig. 13B.** Comparison-adjusted funnel plot for fecal urgency at 6 months postoperatively. **Supplementary Fig. 14A.** Network plot for incomplete defecation at 6 months postoperatively. **Supplementary Table 17A.** Relative effects table for incomplete defecation at 6 months postoperatively. **Supplementary Table 17B.** Rank probabilities for incomplete defecation at 6 months postoperatively. **Supplementary Fig. 14B.** Comparison-adjusted funnel plot for incomplete defecation at 6 months postoperatively. **Supplementary Fig. 15A.** Network plot for use of antidiarrheal medication at 6 months postoperatively. **Supplementary Table 18A.** Relative effects table for use of antidiarrheal medication at 6 months postoperatively. **Supplementary Table 18B.** Rank probabilities for use of antidiarrheal medication at 6 months postoperatively. **Supplementary Fig. 15B.** Comparison-adjusted funnel plot for use of antidiarrheal medication at 6 months postoperatively. **Supplementary Fig. 16A.** Network plot for defecation frequency at 12 months postoperatively. **Supplementary Table 19B.** Rank probabilities for defecation frequency at 12 months postoperatively. **Supplementary Fig. 16B.** Comparison-adjusted funnel plot for defecation frequency at 12 months postoperatively. **Supplementary Fig. 17A.** Network plot for fecal urgency at 12 months postoperatively. **Supplementary Table 20A.** Relative effects table for fecal urgency at 12 months postoperatively. **Supplementary Table 20B.** Rank probabilities for fecal urgency at 12 months postoperatively. **Supplementary Fig. 17B.** Comparison-adjusted funnel plot for fecal urgency at 12 months postoperatively. **Supplementary Fig. 18A.** Network plot for incomplete defecation at 12 months postoperatively. **Supplementary Table 21A.** Relative effects table for incomplete defecation at 12 months postoperatively. **Supplementary Table 21B.** Rank probabilities for incomplete defecation at 12 months postoperatively. **Supplementary Fig. 18B.** Comparison-adjusted funnel plot for incomplete defecation at 12 months postoperatively. **Supplementary Fig. 19A.** Network plot for use of antidiarrheal medication at 12 months postoperatively. **Supplementary Table 22A.** Relative effects table for use of antidiarrheal medication at 12 months postoperatively. **Supplementary Table 22B.** Rank probabilities for use of antidiarrheal medication at 12 months postoperatively. **Supplementary Fig. 19B.** Comparison-adjusted funnel plot for use of antidiarrheal medication at 12 months postoperatively. **Supplementary Fig. 20A.** Network plot for defecation frequency at 24 months postoperatively. **Supplementary Table 23B.** Rank probabilities for defecation frequency at 24 months postoperatively. **Supplementary Fig. 20B.** Comparison-adjusted funnel plot for defecation frequency at 24 months postoperatively. **Supplementary Fig. 21A.** Network plot for use of antidiarrheal medication at 24 months postoperatively. **Supplementary Table 24A.** Relative effects table for use of antidiarrheal medication at 24 months postoperatively. **Supplementary Table 24B.** Rank probabilities for use of antidiarrheal medication at 24 months postoperatively. **Supplementary Fig. 21B.** Comparison-adjusted funnel plot for use of antidiarrheal medication at 24 months postoperatively. **Supplementary Table 25A.** Relative effects table for postoperative anastomotic leakage in the sensitivity analysis. **Supplementary Table 25B.** Rank probabilities for postoperative anastomotic leakage in the sensitivity analysis.

## Data Availability

All data generated or analyzed during this study are included in this article (and its additional file).

## Abbreviations

CJP: Colon J-pouch
SCA: Straight colorectal anastomosis
TCP: Transverse coloplasty
SEA: Side-to-end anastomosis
DIC: Deviance information criterion
SUCRA: Surface under the cumulative ranking curve
LAR: Low anterior resection
TME: Total mesorectal excision
RCTs: Randomized controlled trials
PRISMA: Preferred reporting items for systematic reviews and meta-analyses
RR: Risk ratio
MD: Mean difference
CI: Confidence interval
