# Supplementary material for: Comparison of complications and bowel function among different reconstruction techniques after low anterior resection for rectal cancer: a systematic review and network meta-analysis
Source: World J Surg Oncol. 2023 Mar 10;21:87. doi: 10.1186/s12957-023-02977-z (PMC9999608; doi:10.1186/s12957-023-02977-z)
Supplement: Supplementary file 1 — Additional file 1: Supplementary Table 1. Checklist of the PRISMA extension for network meta-analysis. Supplementary Table 2. Number of citations by each database searched. Supplementary Table 3. Characteristics of the 29 studies included in the network Meta-analysis. Supplementary Fig. 1. Risk-of-bias summary of the randomized controlled trials. Supplementary Table 4. Quality assessment of included randomized controlled trials. Supplementary Table 5. Results of global heterogeneity and local heterogeneity. Supplementary Table 6. Node-splitting analysis of inconsistency. Supplementary Table 7. Comparisons of the fitness of consistency and inconsistency models using deviance information criteria. Supplementary Fig. 2. Results of pairwise meta-analysis for postoperative complications. Supplementary Fig. 3. Results of pairwise meta-analysis for defecation frequency. Supplementary Fig. 4. Results of pairwise meta-analysis for bowel function. Supplementary Table 8A. Relative effects table for postoperative anastomotic leakage. Supplementary Table 8B. Rank probabilities for postoperative anastomotic leakage. Supplementary Fig. 5B. Comparison-adjusted funnel plot for postoperative anastomotic leakage. Supplementary Fig. 6A. Network plot for postoperative anastomotic stricture. Supplementary Table 9A. Relative effects table for postoperative anastomotic stricture. Supplementary Table 9B. Rank probabilities for postoperative anastomotic stricture. Supplementary Fig. 6B. Comparison-adjusted funnel plot for postoperative anastomotic stricture. Supplementary Fig. 7A. Network plot postoperative reoperation. Supplementary Table 10A. Relative effects table for postoperative reoperation. Supplementary Table 10B. Rank probabilities for postoperative reoperation. Supplementary Fig. 7B. Comparison-adjusted funnel plot for postoperative reoperation. Supplementary Fig. 8A. Network plot for postoperative mortality within 30 days. Supplementary Table 11A. Relative effects table for postop [file 12957_2023_2977_MOESM1_ESM.docx]

**Comparison of complications and bowel function among different reconstruction techniques after low anterior resection for rectal cancer: a systematic review and network meta-analysis**

**Supplementary Material**

**Page 5-9**

**Supplementary Table 1** Checklist of the PRISMA extension for network meta-analysis.

**Page 10-12**

**Supplementary Table 2** Number of citations by each database searched.

**Page 13-14**

**Supplementary Table 3** Characteristics of the 29 studies included in the network Meta-analysis.

**Page 15-16**

**Supplementary Fig. 1** Risk-of-bias summary of the randomized controlled trials.

**Supplementary Table 4** Quality assessment of included randomized controlled trials.

**Page 17-22**

**Supplementary Table 5** Results of global heterogeneity and local heterogeneity.

**Supplementary Table 6** Node-splitting analysis of inconsistency.

**Supplementary Table 7** Comparisons of the fitness of consistency and inconsistency models using deviance information criteria.

**Page 23-25**

**Supplementary Fig. 2** Results of pairwise meta-analysis for postoperative complications.

**Supplementary Fig. 3** Results of pairwise meta-analysis for defecation frequency.

**Supplementary Fig. 4** Results of pairwise meta-analysis for bowel function.

**Page 26-88**

**Supplementary Table 8A** Relative effects table for postoperative anastomotic leakage.

**Supplementary Table 8B** Rank probabilities for postoperative anastomotic leakage.

**Supplementary Fig. 5B** Comparison-adjusted funnel plot for postoperative anastomotic leakage.

**Supplementary Fig. 6A** Network plot for postoperative anastomotic stricture.

**Supplementary Table 9A** Relative effects table for postoperative anastomotic stricture.

**Supplementary Table 9B** Rank probabilities for postoperative anastomotic stricture.

**Supplementary Fig. 6B** Comparison-adjusted funnel plot for postoperative anastomotic stricture.

**Supplementary Fig. 7A** Network plot postoperative reoperation.

**Supplementary Table 10A** Relative effects table for postoperative reoperation.

**Supplementary Table 10B** Rank probabilities for postoperative reoperation.

**Supplementary Fig. 7B** Comparison-adjusted funnel plot for postoperative reoperation.

**Supplementary Fig. 8A** Network plot for postoperative mortality within 30 days.

**Supplementary Table 11A** Relative effects table for postoperative mortality within 30 days.

**Supplementary Table 11B** Rank probabilities for postoperative mortality within 30 days.

**Supplementary Fig. 8B** Comparison-adjusted funnel plot for postoperative mortality within 30 days.

**Supplementary Fig. 9A** Network plot for defecation frequency at 3 months postoperatively.

**Supplementary Table 12B** Rank probabilities for defecation frequency at 3 months postoperatively.

**Supplementary Fig. 9B** Comparison-adjusted funnel plot for defecation frequency at 3 months postoperatively.

**Supplementary Fig. 10A** Network plot for fecal urgency at 3 months postoperatively.

**Supplementary Table 13A** Relative effects table for fecal urgency at 3 months postoperatively.

**Supplementary Table 13B** Rank probabilities for fecal urgency at 3 months postoperatively.

**Supplementary Fig. 10B** Comparison-adjusted funnel plot for fecal urgency at 3 months postoperatively.

**Supplementary Fig. 11A** Network plot for use of antidiarrheal medication at 3 months postoperatively.

**Supplementary Table 14A** Relative effects table for use of antidiarrheal medication at 3 months postoperatively.

**Supplementary Table 14B** Rank probabilities for use of antidiarrheal medication at 3 months postoperatively.

**Supplementary Fig. 11B** Comparison-adjusted funnel plot for use of antidiarrheal medication at 3 months postoperatively.

**Supplementary Fig. 12A** Network plot for defecation frequency at 6 months postoperatively.

**Supplementary Table 15B** Rank probabilities for defecation frequency at 6 months postoperatively.

**Supplementary Fig .12B** Comparison-adjusted funnel plot for defecation frequency at 6 months postoperatively.

**Supplementary Fig. 13A** Network plot for fecal urgency at 6 months postoperatively.

**Supplementary Table 16A** Relative effects table for fecal urgency at 6 months postoperatively.

**Supplementary Table 16B** Rank probabilities for fecal urgency at 6 months postoperatively.

**Supplementary Fig. 13B** Comparison-adjusted funnel plot for fecal urgency at 6 months postoperatively.

**Supplementary Fig. 14A** Network plot for incomplete defecation at 6 months postoperatively.

**Supplementary Table 17A** Relative effects table for incomplete defecation at 6 months postoperatively.

**Supplementary Table 17B** Rank probabilities for incomplete defecation at 6 months postoperatively.

**Supplementary Fig. 14B** Comparison-adjusted funnel plot for incomplete defecation at 6 months postoperatively.

**Supplementary Fig. 15A** Network plot for use of antidiarrheal medication at 6 months postoperatively.

**Supplementary Table 18A** Relative effects table for use of antidiarrheal medication at 6 months postoperatively.

**Supplementary Table 18B** Rank probabilities for use of antidiarrheal medication at 6 months postoperatively.

**Supplementary Fig. 15B** Comparison-adjusted funnel plot for use of antidiarrheal medication at 6 months postoperatively.

**Supplementary Fig. 16A** Network plot for defecation frequency at 12 months postoperatively.

**Supplementary Table 19B** Rank probabilities for defecation frequency at 12 months postoperatively.

**Supplementary Fig. 16B** Comparison-adjusted funnel plot for defecation frequency at 12 months postoperatively.

**Supplementary Fig. 17A** Network plot for fecal urgency at 12 months postoperatively.

**Supplementary Table 20A** Relative effects table for fecal urgency at 12 months postoperatively.

**Supplementary Table 20B** Rank probabilities for fecal urgency at 12 months postoperatively.

**Supplementary Fig. 17B** Comparison-adjusted funnel plot for fecal urgency at 12 months postoperatively.

**Supplementary Fig. 18A** Network plot for incomplete defecation at 12 months postoperatively.

**Supplementary Table 21A** Relative effects table for incomplete defecation at 12 months postoperatively.

**Supplementary Table 21B** Rank probabilities for incomplete defecation at 12 months postoperatively.

**Supplementary Fig. 18B** Comparison-adjusted funnel plot for incomplete defecation at 12 months postoperatively.

**Supplementary Fig. 19A** Network plot for use of antidiarrheal medication at 12 months postoperatively.

**Supplementary Table 22A** Relative effects table for use of antidiarrheal medication at 12 months postoperatively.

**Supplementary Table 22B** Rank probabilities for use of antidiarrheal medication at 12 months postoperatively.

**Supplementary Fig. 19B** Comparison-adjusted funnel plot for use of antidiarrheal medication at 12 months postoperatively.

**Supplementary Fig. 20A** Network plot for defecation frequency at 24 months postoperatively.

**Supplementary Table 23B** Rank probabilities for defecation frequency at 24 months postoperatively.

**Supplementary Fig. 20B** Comparison-adjusted funnel plot for defecation frequency at 24 months postoperatively.

**Supplementary Fig. 21A** Network plot for use of antidiarrheal medication at 24 months postoperatively.

**Supplementary Table 24A** Relative effects table for use of antidiarrheal medication at 24 months postoperatively.

**Supplementary Table 24B** Rank probabilities for use of antidiarrheal medication at 24 months postoperatively.

**Supplementary Fig. 21B** Comparison-adjusted funnel plot for use of antidiarrheal medication at 24 months postoperatively.

**Page 89-90**

**Supplementary Table 25A** Relative effects table for postoperative anastomotic leakage in the sensitivity analysis.

**Supplementary Table 25B** Rank probabilities for postoperative anastomotic leakage in the sensitivity analysis.

**Supplementary Table 1** Checklist of the PRISMA extension for network meta-analysis.

| **Section/Topic** | **Item #** | **Checklist Item** | **Reported**  **on Page #** |
| --- | --- | --- | --- |
| **TITLE** |  |  |  |
| Title | 1 | Identify the report as a systematic review *incorporating a network meta-analysis (or related form of meta-analysis).* | 1 |
| **ABSTRACT** |  |  |  |
| Structured summary | 2 | Provide a structured summary including, as applicable:  **Background:** main objectives  **Methods:** data sources; study eligibility criteria, participants, and interventions; study appraisal; and *synthesis methods, such as network meta-analysis.*  **Results:** number of studies and participants identified; summary estimates with corresponding confidence/credible intervals; *treatment rankings may also be discussed. Authors may choose to summarize pairwise comparisons against a chosen treatment included in their analyses for brevity.*  **Discussion/Conclusions:** limitations; conclusions and implications of findings.  **Other:** primary source of funding; systematic review registration number with registry name. | 2-3 |
| **INTRODUCTION** |  |  |  |
| Rationale | 3 | Describe the rationale for the review in the context of what is already known*, including mention of why a network meta-analysis has been conducted.* | 3-4 |
| Objectives | 4 | Provide an explicit statement of questions being addressed, with reference to participants, interventions, comparisons, outcomes, and study design (PICOS). | 4 |
| **METHODS** |  |  |  |
| Protocol and registration | 5 | Indicate whether a review protocol exists and if and where it can be accessed (e.g., Web address); and, if available, provide registration information, including registration number. | 4 |
| Eligibility criteria | 6 | Specify study characteristics (e.g., PICOS, length of follow-up) and report characteristics (e.g., years considered, language, publication status) used as criteria for eligibility, giving rationale. *Clearly describe eligible treatments included in the treatment network, and note whether any have been clustered or merged into the same node (with justification).* | 5 |
| Information sources | 7 | Describe all information sources (e.g., databases with dates of coverage, contact with study authors to identify additional studies) in the search and date last searched. | 4-5 |
| Search | 8 | Present full electronic search strategy for at least one database, including any limits used, such that it could be repeated. | 10-12 (Supplementary File 1) |
| Study selection | 9 | State the process for selecting studies (i.e., screening, eligibility, included in systematic review, and, if applicable, included in the meta-analysis). | 4-5 |
| Data collection process | 10 | Describe method of data extraction from reports (e.g., piloted forms, independently, in duplicate) and any processes for obtaining and confirming data from investigators. | 5-6 |
| Data items | 11 | List and define all variables for which data were sought (e.g., PICOS, funding sources) and any assumptions and simplifications made. | 5-6 |
| **Geometry of the network** | **S1** | Describe methods used to explore the geometry of the treatment network under study and potential biases related to it. This should include how the evidence base has been graphically summarized for presentation, and what characteristics were compiled and used to describe the evidence base to readers. | 6-7 |
| Risk of bias within individual studies | 12 | Describe methods used for assessing risk of bias of individual studies (including specification of whether this was done at the study or outcome level), and how this information is to be used in any data synthesis. | 6 |
| Summary measures | 13 | State the principal summary measures (e.g., risk ratio, difference in means). *Also describe the use of additional summary measures assessed, such as treatment rankings and surface under the cumulative ranking curve (SUCRA) values, as well as modified approaches used to present summary findings from meta-analyses.* | 6-7 |
| Planned methods of analysis | 14 | Describe the methods of handling data and combining results of studies for each network meta-analysis. This should include, but not be limited to:   - *Handling of multi-arm trials;* - *Selection of variance structure;* - *Selection of prior distributions in Bayesian analyses; and* - *Assessment of model fit.* | 6-7 |
| **Assessment of Inconsistency** | **S2** | Describe the statistical methods used to evaluate the agreement of direct and indirect evidence in the treatment network(s) studied. Describe efforts taken to address its presence when found. | 6-7 |
| Risk of bias across studies | 15 | Specify any assessment of risk of bias that may affect the cumulative evidence (e.g., publication bias, selective reporting within studies). | 6-7 |
| Additional analyses | 16 | Describe methods of additional analyses if done, indicating which were pre-specified. This may include, but not be limited to, the following:   - Sensitivity or subgroup analyses; - Meta-regression analyses; - *Alternative formulations of the treatment network; and* - *Use of alternative prior distributions for Bayesian analyses (if applicable).* | 6-7 |
| **RESULTS†** |  |  |  |
| Study selection | 17 | Give numbers of studies screened, assessed for eligibility, and included in the review, with reasons for exclusions at each stage, ideally with a flow diagram. | 7-8, Fig. 1 |
| **Presentation of network structure** | **S3** | Provide a network graph of the included studies to enable visualization of the geometry of the treatment network. | 9, Fig. 2,  Fig. 6A-21A (Supplementary File 1) |
| **Summary of network geometry** | **S4** | Provide a brief overview of characteristics of the treatment network. This may include commentary on the abundance of trials and randomized patients for the different interventions and pairwise comparisons in the network, gaps of evidence in the treatment network, and potential biases reflected by the network structure. | 7-8 |
| Study characteristics | 18 | For each study, present characteristics for which data were extracted (e.g., study size, PICOS, follow-up period) and provide the citations. | 7-8, 13-14 (Supplementary File 1) |
| Risk of bias within studies | 19 | Present data on risk of bias of each study and, if available, any outcome level assessment. | 8, 15-16 (Supplementary File 1) |
| Results of individual studies | 20 | For all outcomes considered (benefits or harms), present, for each study: 1) simple summary data for each intervention group, and 2) effect estimates and confidence intervals. *Modified approaches may be needed to deal with information from larger networks.* | 8-9, 23-25 (Supplementary File 1) |
| Synthesis of results | 21 | Present results of each meta-analysis done, including confidence/credible intervals. *In larger networks, authors may focus on comparisons versus a particular comparator (e.g. placebo or standard care), with full findings presented in an appendix. League tables and forest plots may be considered to summarize pairwise comparisons.* If additional summary measures were explored (such as treatment rankings), these should also be presented. | 9-11, Fig. 2-3, Table 1-2, Table 8A-24A (Supplementary File 1), Table 8B-24B (Supplementary File 1) |
| **Exploration for inconsistency** | **S5** | Describe results from investigations of inconsistency. This may include such information as measures of model fit to compare consistency and inconsistency models, *P* values from statistical tests, or summary of inconsistency estimates from different parts of the treatment network. | 11-12, 17-22 (Supplementary File 1) |
| Risk of bias across studies | 22 | Present results of any assessment of risk of bias across studies for the evidence base being studied. | 8, 15-16 (Supplementary File 1) |
| Results of additional analyses | 23 | Give results of additional analyses, if done (e.g., sensitivity or subgroup analyses, meta-regression analyses*, alternative network geometries studied, alternative choice of prior distributions for Bayesian analyses,* and so forth). | 11-12, 89-90 (Supplementary File 1) |
| **DISCUSSION** |  |  |  |
| Summary of evidence | 24 | Summarize the main findings, including the strength of evidence for each main outcome; consider their relevance to key groups (e.g., healthcare providers, users, and policy-makers). | 12-16 |
| Limitations | 25 | Discuss limitations at study and outcome level (e.g., risk of bias), and at review level (e.g., incomplete retrieval of identified research, reporting bias). *Comment on the validity of the assumptions, such as transitivity and consistency. Comment on any concerns regarding network geometry (e.g., avoidance of certain comparisons).* | 16-17 |
| Conclusions | 26 | Provide a general interpretation of the results in the context of other evidence, and implications for future research. | 17 |
| **FUNDING** |  |  |  |
| Funding | 27 | Describe sources of funding for the systematic review and other support (e.g., supply of data); role of funders for the systematic review. This should also include information regarding whether funding has been received from manufacturers of treatments in the network and/or whether some of the authors are content experts with professional conflicts of interest that could affect use of treatments in the network. | 18 |

PICOS = population, intervention, comparators, outcomes, study design.

* Text in italics indicateS wording specific to reporting of network meta-analyses that has been added to guidance from the PRISMA statement.

† Authors may wish to plan for use of appendices to present all relevant information in full detail for items in this section.

**Supplementary Table 2** Number of citations by each database searched.

| **Database** | **Citations** |
| --- | --- |
| Pubmed | 210 |
| Embase | 127 |
| Cochrane | 134 |
| **Total** | 471 |

**Search strategy for Pubmed**

#1 "Rectal Neoplasms"[MeSH Terms]

#2 ((((((((((((((((Neoplasm, Rectal[Title/Abstract]) OR (Rectal Neoplasm[Title/Abstract])) OR (Rectum Neoplasms[Title/Abstract])) OR (Neoplasm, Rectum[Title/Abstract])) OR (Rectum Neoplasm[Title/Abstract])) OR (Rectal Tumors[Title/Abstract])) OR (Rectal Tumor[Title/Abstract])) OR (Tumor, Rectal[Title/Abstract])) OR (Neoplasms, Rectal[Title/Abstract])) OR (Cancer of Rectum[Title/Abstract])) OR (Rectum Cancers[Title/Abstract])) OR (Rectal Cancer[Title/Abstract])) OR (Cancer, Rectal[Title/Abstract])) OR (Rectal Cancers[Title/Abstract])) OR (Rectum Cancer[Title/Abstract])) OR (Cancer, Rectum[Title/Abstract])) OR (Cancer of the Rectum[Title/Abstract])

#3 (#1) OR (#2)

#4 "anastomosis, surgical"[MeSH Terms]

#5 (((((Surgical Anastomosis[Title/Abstract]) OR (Anastomoses, Surgical[Title/Abstract])) OR (Surgical Anastomoses[Title/Abstract])) OR (side-to-end[Title/Abstract])) OR (end-to-side[Title/Abstract])) OR (baker[Title/Abstract])

#6 (#4) OR (#5)

#7 ((((Surgical Anastomosis[Title/Abstract]) OR (Anastomoses, Surgical[Title/Abstract])) OR (Surgical Anastomoses[Title/Abstract])) OR (end-to-end[Title/Abstract])) OR (straight colorectal anastomosis[Title/Abstract])

#8 (#4) OR (#7)

#9 "Colonic Pouches"[Mesh]

#10 ((((((((((((((((((((((((((((Colonic Pouche[Title/Abstract]) OR (Pouche, Colonic[Title/Abstract])) OR (Pouches, Colonic[Title/Abstract])) OR (Pelvic Pouches[Title/Abstract])) OR (Pouches, Pelvic[Title/Abstract])) OR (Ileoanal Pouches[Title/Abstract])) OR (Pouches, Ileoanal[Title/Abstract])) OR (Ileoanal Reservoirs[Title/Abstract])) OR (Ileoanal Reservoir[Title/Abstract])) OR (Reservoir, Ileoanal[Title/Abstract])) OR (Reservoirs, Ileoanal[Title/Abstract])) OR (W-Pouch[Title/Abstract])) OR (W Pouch[Title/Abstract])) OR (Kock Pouch[Title/Abstract])) OR (Pouch, Kock[Title/Abstract])) OR (S-Pouch[Title/Abstract])) OR (S Pouch[Title/Abstract])) OR (Ileal Pouches[Title/Abstract])) OR (Ileal Pouche[Title/Abstract])) OR (Pouche, Ileal[Title/Abstract])) OR (Pouches, Ileal[Title/Abstract])) OR (Ileal Reservoirs[Title/Abstract])) OR (Ileal Reservoir[Title/Abstract])) OR (Reservoir, Ileal[Title/Abstract])) OR (Reservoirs, Ileal[Title/Abstract])) OR (J-Pouch[Title/Abstract])) OR (J Pouch[Title/Abstract])) OR (colonic-J-pouch[Title/Abstract])) OR (coloanal-J-pouch[Title/Abstract])

#11 (#9) OR (#10)

#12 ((((transverse coloplasty[Title/Abstract]) OR (TCP[Title/Abstract])) OR (coloplasty pouch[Title/Abstract])) OR (coloplasty[Title/Abstract])) OR (transverse coloplasty pouch[Title/Abstract])

#13 randomized controlled trial[Publication Type] OR randomized[Title/Abstract] OR placebo[Title/Abstract]

#14 (#6) OR (#8) OR (#11) OR (#12)

#15 (#3) AND (#13) AND (#14)

**Search strategy for Embase**

#1 'rectum cancer'/exp

#2 'rectal neoplasms':ab,ti OR 'neoplasm, rectal':ab,ti OR 'rectal neoplasm':ab,ti OR 'rectum neoplasms':ab,ti OR 'neoplasm, rectum':ab,ti OR 'rectum neoplasm':ab,ti OR 'rectal tumors':ab,ti OR 'rectal tumor':ab,ti OR 'tumor, rectal':ab,ti OR 'neoplasms, rectal':ab,ti OR 'cancer of rectum':ab,ti OR 'rectum cancers':ab,ti OR 'rectal cancer':ab,ti OR 'cancer, rectal':ab,ti OR 'rectal cancers':ab,ti OR 'cancer, rectum':ab,ti OR 'cancer of the rectum':ab,ti OR 'rectal malignancy':ab,ti OR 'rectum malignancy':ab,ti

#3 (#1) OR (#2)

#4 'end to side anastomosis'/exp

#5 'side to end':ab,ti OR 'end to side':ab,ti OR baker:ab,ti

#6 (#4) OR (#5)

#7 'end to end anastomosis'/exp

#8 'straight colorectal anastomosis':ab,ti

#9 (#7) OR (#8)

#10 'ileal pouch-anal anastomosis'/exp

#11 'colonic pouches':ab,ti OR 'colonic pouche':ab,ti OR 'pouche, colonic':ab,ti OR 'pouches, colonic':ab,ti OR 'pelvic pouches':ab,ti OR 'pouches, pelvic':ab,ti OR 'ileoanal pouches':ab,ti OR 'pouches, ileoanal':ab,ti OR 'ileoanal reservoirs':ab,ti OR 'ileoanal reservoir':ab,ti OR 'reservoir, ileoanal':ab,ti OR 'reservoirs, ileoanal':ab,ti OR 'w pouch':ab,ti OR 'kock pouch':ab,ti OR 'pouch, kock':ab,ti OR 's pouch':ab,ti OR 's pouchs':ab,ti OR 'ileal pouches':ab,ti OR 'ileal pouche':ab,ti OR 'pouche, ileal':ab,ti OR 'pouches, ileal':ab,ti OR 'ileal reservoirs':ab,ti OR 'ileal reservoir':ab,ti OR 'reservoir, ileal':ab,ti OR 'reservoirs, ileal':ab,ti OR 'j pouch':ab,ti

#12 (#10) OR (#11)

#13 'transverse coloplasty':ab,ti OR tcp:ab,ti OR 'coloplasty pouch':ab,ti OR coloplasty:ab,ti OR 'transverse coloplasty pouch':ab,ti

#14 (#6) OR (#9) OR (#12) OR (#13)

#15 'randomized controlled trial'/exp OR 'randomized controlled trial' OR (randomized AND controlled AND ('trial'/exp OR trial)) OR randomized OR 'placebo'/exp OR placebo

#16 (#3) AND (#14) AND (#15)

**Search strategy for Cochrane**

#1 MeSH descriptor: [Rectal Neoplasms] explode all trees

#2 (Neoplasm, Rectal):ti,ab,kw OR (Rectal Neoplasm):ti,ab,kw OR (Rectum Neoplasms):ti,ab,kw OR (Neoplasm, Rectum):ti,ab,kw OR (Rectum Neoplasm):ti,ab,kw

#3 (Rectal Tumors):ti,ab,kw OR (Rectal Tumor):ti,ab,kw OR (Tumor, Rectal):ti,ab,kw OR (Neoplasms, Rectal):ti,ab,kw OR (Cancer of Rectum):ti,ab,kw

#4 (Rectum Cancers):ti,ab,kw OR (Rectal Cancer):ti,ab,kw OR (Cancer, Rectal):ti,ab,kw OR (Rectal Cancers):ti,ab,kw OR (Rectum Cancer):ti,ab,kw

#5 (Cancer, Rectum):ti,ab,kw OR (Cancer of the Rectum):ti,ab,kw

#6 (#1) OR (#2) OR (#3) OR (#4) OR (#5)

#7 (side-to-end):ti,ab,kw OR (end-to-side):ti,ab,kw OR (baker):ti,ab,kw

#8 (end-to-end):ti,ab,kw OR (straight colorectal anastomosis):ti,ab,kw

#9 (J pouch):ti,ab,kw OR (J-pouch):ti,ab,kw OR (colonic-J-pouch):ti,ab,kw OR (coloanal-J-pouch):ti,ab,kw

#10 (transverse coloplasty):ti,ab,kw OR (TCP):ti,ab,kw OR (coloplasty pouch):ti,ab,kw OR (coloplasty):ti,ab,kw OR (Transverse coloplasty pouch):ti,ab,kw

#11 (#7) OR (#8) OR (#9) OR (#10)

#12 (#6) AND (#11)

**Supplementary Table 3** Characteristics of the 29 studies included in the network Meta-analysis.

| **Study** | **Country** | **Study**  **period** | **Multicenter** | **Group** | **Number** | **Age**  **(year)** | **Gender**  **(M/F)** | **Tumour level**  **(cm)** | **Anastomosis level**  **(cm)** | **protective stoma** | **Tumour stage** |
| --- | --- | --- | --- | --- | --- | --- | --- | --- | --- | --- | --- |
| Ortiz  1995 | Spain | NR | NO | CJP | 19 | 59.5±14.27 | 13/6 | 3(2-4.5)^b^ | 2.14±0.36 | 19 | NR |
|  |  |  |  | SCA | 19 | 67.3±9.2 | 10/9 | 3(2-4.5)^b^ | 3.38±0.56 | 19 | NR |
| Seow-Choen  1995 | Singapore | 1997.1-NR | NO | CJP | 20 | 64.5(38-83) | 13/7 | 6(4-8) | 3(1-4) | 20 | 1/8/11/0 (Dukes) |
|  |  |  |  | SCA | 20 | 62.5(44-86) | 15/5 |  | 3.25(2-5) | 20 | 5/8/6/0 (Dukes) |
| Hallböök  1996 | Sweden | NA-1993.12 | Yes | CJP | 45 | 67(40-86) | 20/25 | 7(4-12) | 3.5(2-2.5) | 32 | 8/19/17/1 (Dukes) |
|  |  |  |  | SCA | 52 | 69(29-82) | 25/27 | 7(3.5-12) | 4(2-6) | 31 | 13/21/17/1 (Dukes) |
| Huber  1999 | Germany | 1995.10-  1996.10 | NO | CJP | 29 | 62.3 | 13/16 | 5.2(2.5-9) | 3.8(2-5) | 23 | 1/12/17/0 (TNM) |
|  |  |  |  | SEA | 30 | 61.9 | 12/18 | 5.8(3-9) | 4.2(3-5.5) | 21 | 2/13/14/0 (TNM) |
| Ho  2000 | Singapore | 1995.7-  1997.10 | NO | CJP | 21 | 62.1(2.7)^a^ | NR | NR | 3(0.2)^a^ | 21 | 5/4/8/4 (Dukes) |
|  |  |  |  | SCA | 26 | 61.1(2.03)^a^ | NR | NR | 3(0.2)^a^ | 26 | 5/8/5/8 (Dukes) |
| Ho  2001 | Singapore | 1995.11-  1997.8 | NO | CJP | 16 | 61.3±3.2 | 11/5 | 3±0.5^b^ | NR | 16 | 1/6/8/1 (Dukes) |
|  |  |  |  | SCA | 19 | 62.1±2.3 | 17/2 | 3±0.2^b^ | NR | 19 | 1/7/10/1 (Dukes) |
| Fürst  2002 | Germany | 1996.8-  1999.1 | NO | CJP | 37 | 60.4(35-80) | 55/19 | 8.9 | NR | 37 | 27.6%/27.6%/34.5%/10.3% (UICC) |
|  |  |  |  | SCA | 37 |  |  | 8.1 | NR | 37 | 32.3%/21.8%/34.1%/11.8% (UICC) |
| Ho  2002 | Singapore | 1998.10-  2000.4 | NO | CJP | 44 | 68.3(1.2)^a^ | 26/18 | 7.2(0.3)^a^ | 3.4(0.2)^a^ | 44 | 0/11/32/1 (TNM) |
|  |  |  |  | TCP | 44 | 65.4(1.6)^a^ | 27/17 | 7.6(0.4)^a^ | 3.2(0.3)^a^ | 44 | 0/10/33/1 (TNM) |
| Oya  2002 | Japan | 1995.1-  1998.12 | NO | CJP | 21 | 61(47-79) | 8/12 | 6.5(3-8) | 5(3-6) | 12 | 4/5/11/0 (Dukes) |
|  |  |  |  | SCA | 21 | 59(42-80) | 8/13 | 6(3-8) | 5(2.5-6.0) | 2 | 6/4/11/0 (Dukes) |
| Sailer  2002 | Germany | NR | NO | CJP | 32 | 64±11 | 21/11 | 8.7±2.9 | NR | NR | NR |
|  |  |  |  | SCA | 32 | 67±9 | 19/13 | 9.4±2.4 | NR | NR | NR |
| Machado  2003 | Sweden | 1995.10-  1999.4 | NO | CJP | 50 | 67(38–83) | 27/23 | 10(3-15) | 4(2-6) | NR | 10/17/21+2 Adenoma (Dukes) |
|  |  |  |  | SEA | 50 | 66.5(40-87) | 32/18 | 10(4-15) | 4(2-5) | NR | 14/19/16+1 Adenoma (Dukes) |
| Pimentel  2003 | Portugal | 1999.5-  2001.4 | NO | CJP | 15 | 62.3(37-84) | 8/7 | 5.3(2.5-10) | 2.9(1.5-4) | 15 | 3/7/5/0 (TNM) |
|  |  |  |  | TCP | 15 | 60.2(33-83) | 9/6 | 5.1(2.5-9) | 2.7(1.5-3.8) | 15 | 4/7/4/0 (TNM) |
| Jiang  2005 | China | 1998.1-  1999.12 | NO | CJP | 28 | 64.9±2.8 | 12/12 | 7.9(1.5)^a^ | 4.8(0.2) | 9 | 3/10/10/1 (Dukes) |
|  |  |  |  | SEA | 28 | 62.3±3.3 | 15/9 | 8.6(0.3)^a^ | 5.3(0.3) | 7 | 7/8/6/3 (Dukes) |
| Machado  2005 | Sweden | NR | NO | CJP | 36 | 65(38-83) | 18/18 | NR | 4(2-5) | 1 | 9/13/12/0+2 Adenoma (Dukes) |
|  |  |  |  | SEA | 35 | 66(50-87) | 21/14 | NR | 4(2-5) | NR | 9/15/10/0+1 Adenoma (Dukes) |
| Park  2005 | Korea | 1998.9-  2002.1 | NO | CJP | 24 | 57(37-68) | 11/13 | 3.9(3.5-4.5) | NR | 24 | pT stage(T1:T2:T3)2:8:14 ;  pN stage(N0:N1:N2)10:11:3 |
|  |  |  |  | SCA | 26 | 59(32-76) | 18/8 | 4.0(3.5-4.5) | NR | 26 | pT stage(T1:T2:T3)3:7:16;  pN stage(N0:N1:N2)14:8:4 |
| Fazio  2007 | America  et al | 2000.11-  2004.6 | Yes | CJP | 137 | 60.2±12.6 | 79/36 | NR | NR | 137 | NR |
|  |  |  |  | TCP | 178 | 60.4±10.4 | 94/50 | NR | NR | 178 | NR |
|  |  |  |  | SCA | 49 | 60.7±10.9 | 36/2 | NR | NR | 49 | NR |
| Liang  2007 | China | 2004.5-  2006.4 | NO | CJP | 24 | 64.4±10.4 | 13/11 | 6.4±1.2 | NR | 4 | 0/15/9/0 (TNM) |
|  |  |  |  | SCA | 24 | 62.4±9.8 | 14/10 | 6.8±1.6 | NR | 3 | 0/14/10/0 (TNM) |
| Stratilatovas  2011 | Lithuania | 2003-  2007.12 | NO | CJP | 29 | 62.8(30-70) | NR | NR | NR | NR | I-III (UICC) |
|  |  |  |  | TCP | 21 |  | NR | NR | NR | NR |  |
|  |  |  |  | SCA | 31 |  | NR | NR | NR | NR |  |
| Doeksen  2012 | Netherlands | 2002.4-  2007.1 | Yes | CJP | 55 | 66(33-82) | 36/19 | NR | NR | 55 | 22/14/19/0 (TNM) |
|  |  |  |  | SEA | 52 | 66(44-79) | 37/15 | NR | NR | 52 | 12/13/25/2 (TNM) |
| Biondo  2013 | Spain | 2000-2005 | Yes | CJP | 54 | 64.6±9.3 | 39/15 | NR | 1.7±1.1 | 54 | 24/15/15（III-IV）(UICC) |
|  |  |  |  | TCP | 52 | 63.6±11.2 | 40/12 | NR | 1.7±1.2 | 52 | 29/14/9（III-IV）(UICC) |
| Okkabaz  2017 | Turkey | 2009.6-NR | NO | CJP | 29 | 58.9±13.7 | 18/11 | 7.9±3.8^b^ | NR | 29 | 3/5/8/11/2 (UICC) |
|  |  |  |  | SEA | 28 | 59.1±11.9 | 19/9 | 6.2±3.8^b^ | NR | 28 | 3/7/7/9/2 (UICC) |
| Parc  2019 | France  et al | NR | Yes | CJP | 80 | 60.2±9.7 | 59/21 | 3.0(0-4)^b^ | 0-1.5^b^ | 80 | 4/18/14/15/0 (TNM) |
|  |  |  |  | SEA | 87 | 59.6±10.6 | 52/35 | 2.0(0-4)^b^ | 0-0.6^b^ | 87 | 5/21/20/20/0 (TNM) |
| Tsunoda  2008 | Japan | 1999-2004 | NO | CJP | 19 | 60(45-79) | 11/8 | NR | NR | 6 | 11/5/3/0 (Dukes) |
|  |  |  |  | SCA | 13 | 63(48-78) | 9/4 | NR | NR | 0 | 2/5/6/0 (Dukes) |
|  |  |  |  | SEA | 17 | 62(29-89) | 11/6 | NR | NR | 15 | 6/5/6/0 (Dukes) |
| Rybakov  2016 | Russia | 2012.5-  2015.5 | NO | SCA | 40 | 61(55-65) | 20/20 | 8.5±1.3 | 5.1±1.8 | 22 | 2/13/12/7/4 (TNM) |
|  |  |  |  | SEA | 41 | 63(58-69) | 13/28 | 8.4±1.3 | 4.8±1.6 | 25 | 2/10/13/9/3 (TNM) |
| Brisinda  2009 | Italy | 1998.1-  2006.1 | NO | SCA | 37 | 63.7±9.2 | 20/17 | NR | NR | 0 | NR |
|  |  |  |  | SEA | 40 | 67.0±5.9 | 19/21 | NR | NR | 0 | NR |
| Parray  2014 | India | 2007.6-  2009.12 | NO | CJP | 22 | 50.7±8.8 | 11/11 | NR | NR | NR | NR |
|  |  |  |  | SCA | 20 | 47.8±16.9 | 10/10 | NR | NR | NR | NR |
| Pucciarelli  2019 | Italy | 2009.10-  2016.2 | Yes | CJP | 190 | 66(58-72) | 108/82 | 7(5-9) | 4(3-5) | NR | 26/77/38/49/0 (TNM)^c^ |
|  |  |  |  | SCA | 189 | 63(54-72) | 114/75 | 8(6-9) | 4(3-5) | NR | 18/65/53/52/0 (TNM)^c^ |
| Ho  1996 | Singapore | 1991.1-  1995.7 | NO | CJP | 17 | 61.2(3.2)^a^ | 6/11 | NR | 3.0(0.2)^a^ | 17 | 1/7/9/0 (Dukes) |
|  |  |  |  | SCA | 16 | 61.2(3.8)^a^ | 10/6 | NR | 3.1(0.1)^a^ | 16 | 2/7/7/0 (Dukes) |
| Ulrich  2008 | Germany | 2002.10.21-  2005.12.5 | NO | CJP | 73 | 63(23-83) | 45/28 | NR | NR | 73 | 7/23/21/19/3 (TNM) |
|  |  |  |  | TCP | 76 | 62(43-81) | 55/21 | NR | NR | 76 | 5/28/19/21/3 (TNM) |

Continuous variables are indicated by default as mean (standard deviation) or median (range), a indicates the mean (standard error). Tumour level, the distance from the lower edge of the tumour to the anal edge, b indicates the distance from the dentate line. Anastomosis level, the distance of the anastomosis from the anal verge. TNM, T1/T2/T3/T4, c indicates T0/T1/T2/T3/T4; Dukes, A/B/C/D; UICC, I/II/III/IV. NR, not report. CJP, colon J-pouch; SCA, straight colorectal anastomosis; TCP, transverse coloplasty; SEA, side-to-end anastomosis.

**Supplementary Fig. 1** Risk-of-bias summary of the randomized controlled trials.


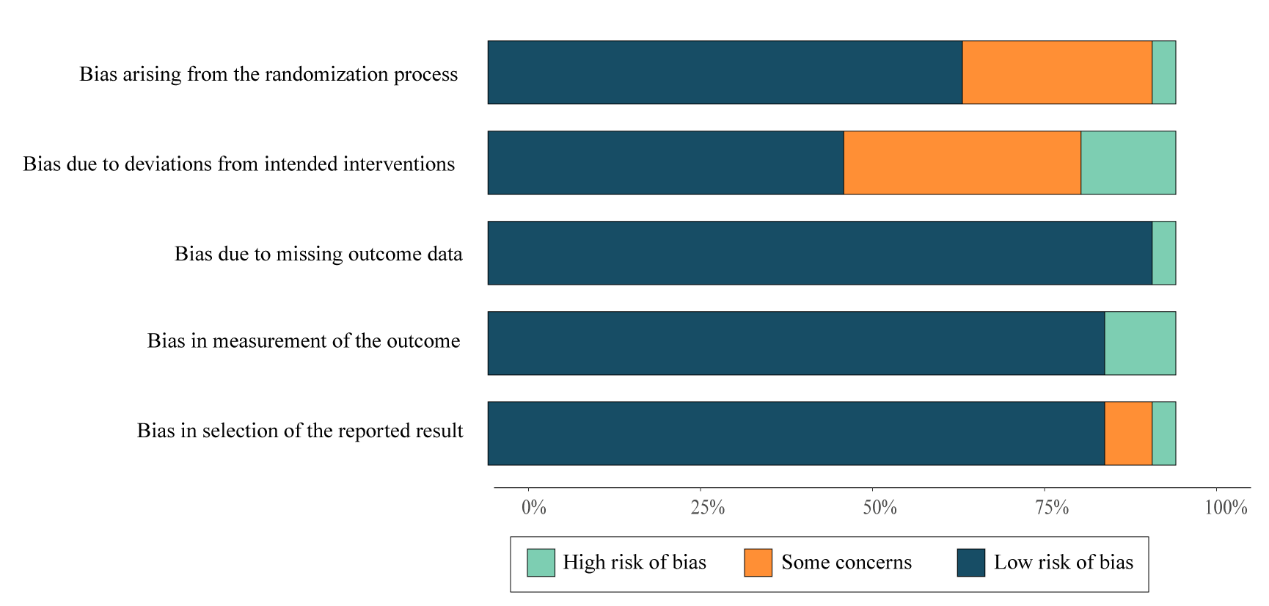


**Supplementary Table 4** Quality assessment of included randomized controlled trials.

| **Study** | **Randomization**  **process** | **Deviations from intended**  **interventions** | **Missing outcome data** | **Measurement of the outcome** | **Selection of the reported result** |
| --- | --- | --- | --- | --- | --- |
| Ortiz 1995 | Some concerns | Low | Low | Low | Low |
| Seow-Choen 1995 | Low | Low | Low | Low | Low |
| Hallböök 1996 | Low | Some concerns | Low | Low | Low |
| Ho 1996 | Low | High | Low | Low | Low |
| Huber 1999 | Low | Low | Low | Low | Low |
| Ho 2000 | Low | Low | Low | Low | Low |
| Ho 2001 | Low | Low | Low | Low | High |
| Fürst 2002 | Some concerns | Some concerns | High | Low | Some concerns |
| Ho 2002 | Low | High | Low | Low | Low |
| Oya 2002 | Low | Low | Low | Low | Low |
| Sailer 2002 | Low | Low | Low | Low | Low |
| Machado 2003 | Low | Low | Low | Low | Low |
| Pimentel 2003 | Some concerns | Low | Low | Low | Low |
| Jiang 2005 | Low | Some concerns | Low | Low | Low |
| Machado 2005 | Some concerns | Low | Low | Low | Low |
| Park 2005 | Some concerns | Low | Low | Low | Some concerns |
| Fazio 2007 | High | Some concerns | Low | Low | Low |
| Liang 2007 | Low | Low | Low | Low | Low |
| Tsunoda 2008 | Some concerns | Low | Low | Low | Low |
| Ulrich 2008 | Low | Some concerns | Low | Low | Low |
| Brisinda 2009 | Some concerns | Some concerns | Low | Low | Low |
| Stratilatovas 2011 | Some concerns | High | Low | Low | Low |
| Doeksen 2012 | Low | Low | Low | High | Low |
| Biondo 2013 | Low | Low | Low | Low | Low |
| Parray 2014 | Low | High | Low | High | Low |
| Rybakov 2016 | Low | Some concerns | Low | Low | Low |
| Okkabaz 2017 | Low | Some concerns | Low | Low | Low |
| Parc 2019 | Low | Some concerns | Low | Low | Low |
| Pucciarelli 2019 | Low | Some concerns | Low | High | Low |

**Supplementary Table 5** Results of global heterogeneity and local heterogeneity.

| **Item** | **Comparisons** | **Local** | | **Global** | | **Heterogeneity assessment** |
| --- | --- | --- | --- | --- | --- | --- |
|  |  | Pairwise ( *I^2^* ) | Network ( *I^2^* ) | Pairwise ( *I^2^* ) | Consistency effect ( *I^2^* ) |  |
| Complication | Anastomotic leakage | | | | | |
|  | SCA vs CJP | 0.5% | 0.0% | 0.0% | 0.0% | Low |
|  | TCP vs CJP | 0.0% | 0.0% |  |  |  |
|  | SEA vs CJP | 15.9% | 9.4% |  |  |  |
|  | TCP vs SCA | 0.0% | 0.0% |  |  |  |
|  | SEA vs SCA | 0.0% | 0.0% |  |  |  |
|  | Anastomotic stricture | | | | | |
|  | SCA vs CJP | 0.0% | 0.0% | 0.0% | 0.0% | Low |
|  | TCP vs CJP | 0.0% | 0.0% |  |  |  |
|  | SEA vs CJP | 28.3% | 19.3% |  |  |  |
|  | TCP vs SCA | NA | 0.0% |  |  |  |
|  | SEA vs SCA | NA | 24.3% |  |  |  |
|  | Reoperation | | | | | |
|  | SCA vs CJP | 0.0% | 0.0% | 37.6% | 42.5% | Low to high |
|  | TCP vs CJP | 28.8% | 28.8% |  |  |  |
|  | SEA vs CJP | 52.0% | 57.6% |  |  |  |
|  | SEA vs SCA | **75.0%** | 51.4% |  |  |  |
|  | Postoperative mortality within 30 days | | | | | |
|  | SCA vs CJP | 17.3% | 17.7% | 12.2% | 12.4% | Low |
|  | TCP vs CJP | 0.0% | 0.0% |  |  |  |
|  | SEA vs CJP | 28.4% | 27.7% |  |  |  |
| Bowel function at 3 months postoperatively | Defecation frequency | | | | | |
|  | SCA vs CJP | **83.9%** | **82.9%** | **95.3%** | **93.5%** | Low to high |
|  | TCP vs CJP | NA | NA |  |  |  |
|  | SEA vs CJP | **96.5%** | **96.4%** |  |  |  |
|  | SEA vs SCA | NA | 0.0% |  |  |  |
|  | Fecal urgency | | | | | |
|  | SCA vs CJP | 60.6% | 31.9% | 22.4% | 15.8% | Low to moderate |
|  | TCP vs CJP | NA | NA |  |  |  |
|  | SEA vs CJP | 10.2% | 0.0% |  |  |  |
|  | SEA vs SCA | 0.0% | 0.0% |  |  |  |
|  | Use of antidiarrheal medication | | | | | |
|  | SCA vs CJP | NA | 0.0% | 0.0% | 4.7% | Low |
|  | TCP vs CJP | NA | NA |  |  |  |
|  | SEA vs CJP | 0.0% | 0.0% |  |  |  |
|  | SEA vs SCA | NA | 0.0% |  |  |  |
| Bowel function at 6 months postoperatively | Defecation frequency | | | | | |
|  | SCA vs CJP | **85.6%** | **85.5%** | **81.6%** | **79.8%** | Low to high |
|  | TCP vs CJP | NA | NA |  |  |  |
|  | SEA vs CJP | 72.3% | 72.1% |  |  |  |
|  | SEA vs SCA | NA | 0.0% |  |  |  |
|  | Fecal urgency | | | | | |
|  | SCA vs CJP | 4.5% | 51.9% | 0.0% | 9.6% | Low to moderate |
|  | TCP vs CJP | NA | NA |  |  |  |
|  | SEA vs CJP | 0.0% | 0.0% |  |  |  |
|  | SEA vs SCA | 0.0% | 0.0% |  |  |  |
|  | Incomplete defecation | | | | | |
|  | SCA vs CJP | 0.0% | 0.0% | 32.3% | 32.2% | Low to moderate |
|  | TCP vs CJP | NA | NA |  |  |  |
|  | SEA vs CJP | 57.3% | 57.2% |  |  |  |
|  | Use of antidiarrheal medication | | | | | |
|  | SCA vs CJP | 45.3% | 0.0% | 24.1% | 1.2% | Low |
|  | TCP vs CJP | 0.0% | 0.0% |  |  |  |
|  | SEA vs CJP | 38.0% | 13.7% |  |  |  |
|  | SEA vs SCA | NA | 0.0% |  |  |  |
| Bowel function at 12 months postoperatively | Defecation frequency | | | | | |
|  | SCA vs CJP | **78.7%** | **81.1%** | **80.1%** | **77.7%** | low to high |
|  | TCP vs CJP | **91.0%** | **87.3%** |  |  |  |
|  | SEA vs CJP | 36.6% | 38.7% |  |  |  |
|  | TCP vs SCA | NA | 59.3% |  |  |  |
|  | Fecal urgency | | | | | |
|  | SCA vs CJP | 13.4% | 17.4% | 0.0% | 0.0% | Low |
|  | TCP vs CJP | 0.0% | 0.0% |  |  |  |
|  | SEA vs CJP | NA | 0.0% |  |  |  |
|  | TCP vs SCA | NA | 0.0% |  |  |  |
|  | SEA vs SCA | NA | 0.0% |  |  |  |
|  | Incomplete defecation | | | | | |
|  | SCA vs CJP | 70.3% | 70.1% | 57.9% | 57.7% | Low to moderate |
|  | TCP vs CJP | NA | NA |  |  |  |
|  | SEA vs CJP | 0.0% | 0.0% |  |  |  |
|  | Use of antidiarrheal medication | | | | | |
|  | SCA vs CJP | **83.1%** | **82.5%** | 74.7% | 73.7% | Low to high |
|  | TCP vs CJP | 0.0% | 0.0% |  |  |  |
|  | SEA vs CJP | NA | NA |  |  |  |
| Bowel function at 24 months postoperatively | Defecation frequency | | | | | |
|  | SCA vs CJP | **99.9%** | **99.9%** | **99.7%** | **99.6%** | Low to high |
|  | TCP vs CJP | NA | NA |  |  |  |
|  | SEA vs CJP | 0.0% | 0.0% |  |  |  |
|  | TCP vs SCA | NA | NA |  |  |  |
|  | Use of antidiarrheal medication | | | | | |
|  | SCA vs CJP | 0.0% | 0.0% | 0.0% | 0.0% | Low |
|  | TCP vs CJP | NA | NA |  |  |  |
|  | SEA vs CJP | NA | NA |  |  |  |
|  | TCP vs SCA | NA | NA |  |  |  |

The numbers with high heterogeneity are in bold (We inferred the magnitude of heterogeneity by comparing the estimated *I^2^* to empirical distributions of heterogeneity typically found in meta-analyses. Low heterogeneity could be considered when the estimated *I^2^* is less than the 50% quantile of the empirical distribution, moderate heterogeneity for *I^2^* between 50% and 75% quantile, and high heterogeneity for *I^2^* larger than the 75% quantile.). NA, Not available (because only one study was included in this type of comparison). CJP, colon J-pouch; SCA, straight colorectal anastomosis; TCP, transverse coloplasty; SEA, side-to-end anastomosis.

**Supplementary Table 6** Node-splitting analysis of inconsistency.

| **Item** | **Comparison** | **Direct effect** | **Indirect effect** | **Overall** | ***P*** |
| --- | --- | --- | --- | --- | --- |
|  |  | RR/MD（95% CI） | | |  |
| Complication | Anastomotic leakage | | | | |
|  | SCA vs CJP | 1.30(0.58, 2.90) | 2.6(0.27, 23.00) | 1.40(0.77, 2.70) | 0.54 |
|  | SEA vs CJP | 0.55(0.21, 1.20) | 0.31(0.05, 2.00) | 0.52(0.22, 1.10) | 0.58 |
|  | TCP vs SCA | 1.30(0.22, 12.00) | 1.80(0.37, 11.00) | 1.50(0.53, 4.50) | 0.79 |
|  | SEA vs SCA | 0.36(0.08, 1.60) | 0.34(0.08, 1.10) | 0.36(0.14, 0.82) | 0.97 |
|  | Anastomotic stricture | | | | |
|  | SCA vs CJP | 1.70(0.47, 5.50) | 0.24(0.01, 6.20) | 1.40(0.44, 3.90) | 0.25 |
|  | SEA vs CJP | 0.88(0.19, 3.90) | 5.90(0.33, 290.00) | 1.20(0.34, 5.00) | 0.23 |
|  | TCP vs SCA | 0.63(0.06, 7.60) | 0.85(0.01, 72.00) | 3.60(0.23, 180.00) | 0.91 |
|  | SEA vs SCA | 3.60(0.23, 180.00) | 0.49(0.07, 3.40) | 0.88(0.20, 4.50) | 0.23 |
|  | Reoperation | | | | |
|  | SCA vs CJP | 1.10(0.22, 5.50) | 5.80(0.06, 280.00) | 1.30(0.26, 5.80) | 0.38 |
|  | SEA vs CJP | 0.57(0.05, 2.00) | 0.10(0.01, 4.30) | 0.41(0.05, 1.50) | 0.37 |
|  | SEA vs SCA | 0.10(0.01, 2.70) | 0.51(0.02, 3.40) | 0.30(0.03, 1.60) | 0.39 |
|  | Postoperative mortality within 30 days | | | | |
|  | NA | NA | NA | NA | NA |
| Bowel function at 3 months postoperatively | Defecation frequency | | | | |
|  | SEA vs SCA | -0.69(-5.40, 4.00) | -1.20(-6.90, 4.30) | -0.81(-4.30, 2.70) | 0.87 |
|  | Fecal urgency | | | | |
|  | SCA vs CJP | 1.60(0.55, 4.60) | 2.70(0.47, 17.00) | 1.90(0.81, 4.40) | 0.58 |
|  | SEA vs CJP | 1.20(0.52, 3.00) | 0.42(0.05, 3.10) | 1.10(0.50, 2.30) | 0.28 |
|  | SEA vs SCA | 0.44(0.17, 1.10) | 1.70(0.28, 11.00) | 0.56(0.25, 1.30) | 0.17 |
|  | Use of antidiarrheal medication | | | | |
|  | SCA vs CJP | 2.70(0.76, 9.90) | 1.30(0.24, 8.70) | 2.20(0.80, 5.90) | 0.49 |
|  | SEA vs CJP | 0.78(0.27, 2.40) | 1.60(0.24, 10.00) | 0.92(0.38, 2.40) | 0.48 |
|  | SEA vs SCA | 0.58(0.14, 2.30) | 0.29(0.06, 1.60) | 0.43(0.15, 1.30) | 0.48 |
| Bowel function at 6 months postoperatively | Defecation frequency | | | | |
|  | SEA vs SCA | -1.90(-4.80, 0.89) | -1.80(-3.40, -0.32) | -1.80(-3.00, -0.73) | 0.92 |
|  | Fecal urgency | | | | |
|  | SCA vs CJP | 1.60(0.37, 15.00) | 2.50(0.10, 95.00) | 1.50(0.63, 6.50) | 0.74 |
|  | SEA vs CJP | 1.40(0.59, 8.50) | 0.49(0.03, 7.70) | 1.10(0.50, 4.40) | 0.33 |
|  | SEA vs SCA | 0.48(0.12, 1.70) | 1.30(0.35, 7.50) | 0.76(0.21, 2.40) | 0.22 |
|  | Incomplete defecation | | | | |
|  | NA | NA | NA | NA | NA |
|  | Use of antidiarrheal medication | | | | |
|  | SCA vs CJP | 3.60(0.82, 36.00) | 1.40(0.04, 80.00) | 3.00(0.92, 19.00) | 0.54 |
|  | SEA vs CJP | 0.65(0.09, 8.00) | 1.70(0.06, 88.00) | 0.83(0.19, 5.80) | 0.54 |
|  | SEA vs SCA | 0.46(0.02, 11.00) | 0.17(0.01, 3.10) | 0.27(0.04, 1.70) | 0.55 |
| Bowel function at 12 months postoperatively | Defecation frequency | | | | |
|  | TCP vs SCA | -0.77(-2.50, 0.87) | -1.60(-3.00, -0.05) | -1.10(-2.10, -0.27) | 0.37 |
|  | Fecal urgency | | | | |
|  | TCP vs SCA | 0.94(0.15, 6.20) | 0.51(0.02, 8.00) | 0.90(0.30, 1.80) | 0.68 |
|  | SEA vs SCA | 0.01(0.01, 0.24) | 0.70(0.26, 2.0) | 0.62(0.18, 1.50) | **0.02** |
|  | Incomplete defecation | | | | |
|  | NA | NA | NA | NA | NA |
|  | Use of antidiarrheal medication | | | | |
|  | NA | NA | NA | NA | NA |
| Bowel function at 24 months postoperatively | Defecation frequency | | | | |
|  | NA | NA | NA | NA | NA |
|  | Use of antidiarrheal medication | | | | |
|  | NA | NA | NA | NA | NA |

Nonsignificant values (*P* > 0.05) indicate no inconsistency between direct and indirect effects. NA, Not available; CJP, colon J-pouch; SCA, straight colorectal anastomosis; TCP, transverse coloplasty; SEA, side-to-end anastomosis.

**Supplementary Table 7** Comparisons of the fitness of consistency and inconsistency models using deviance information criterion.

| **Item** | **Model** | |
| --- | --- | --- |
|  | Consistency | Inconsistency |
| Complication | | |
| Anastomotic leakage | 77.68 | 83.17 |
| Anastomotic stricture | 37.89 | 41.52 |
| Reoperation | 49.36 | 45.88 |
| Postoperative mortality within 30 days | 36.12 | 32.85 |
| Bowel function at 3 months postoperatively | | |
| Defecation frequency | 21.71 | 21.96 |
| Fecal urgency | 24.41 | 26.54 |
| Use of antidiarrheal medication | 18.50 | 20.47 |
| Bowel function at 6 months postoperatively | | |
| Defecation frequency | 53.69 | 53.97 |
| Fecal urgency | 32.89 | 33.16 |
| Incomplete defecation | 26.06 | 27.95 |
| Use of antidiarrheal medication | 31.25 | 31.24 |
| Bowel function at 12 months postoperatively | | |
| Defecation frequency | 50.63 | 50.01 |
| Fecal urgency | 35.35 | 36.97 |
| Incomplete defecation | 24.05 | 24.01 |
| Use of antidiarrheal medication | 23.18 | 23.56 |
| Bowel function at 24months postoperatively | | |
| Defecation frequency | 17.75 | 18.02 |
| Use of antidiarrheal medication | 12.49 | 14.28 |

**Supplementary Fig. 2** Results of pairwise meta-analysis for postoperative complications.
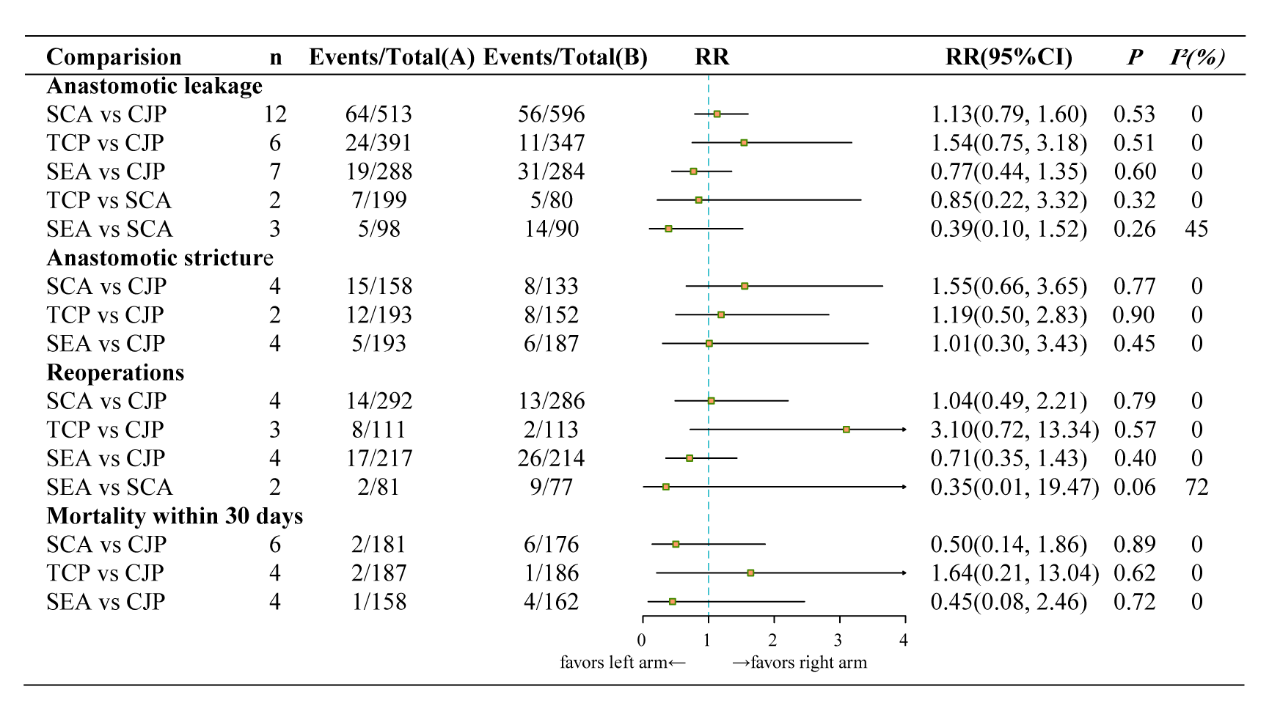


n, number of randomized trials included. CJP, colon J-pouch; SCA, straight colorectal anastomosis; TCP, transverse coloplasty; SEA, side-to-end anastomosis.

**Supplementary Fig. 3** Results of pairwise meta-analysis for defecation frequency.
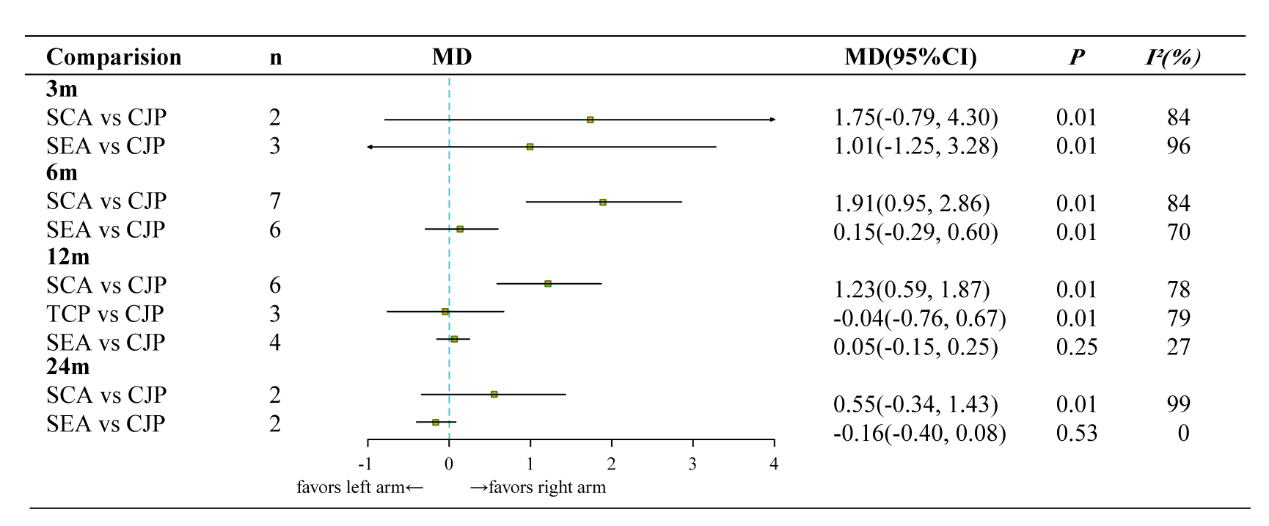


n, number of randomized trials included. CJP, colon J-pouch; SCA, straight colorectal anastomosis; TCP, transverse coloplasty; SEA, side-to-end anastomosis.

**Supplementary Fig. 4** Results of pairwise meta-analysis for bowel function.
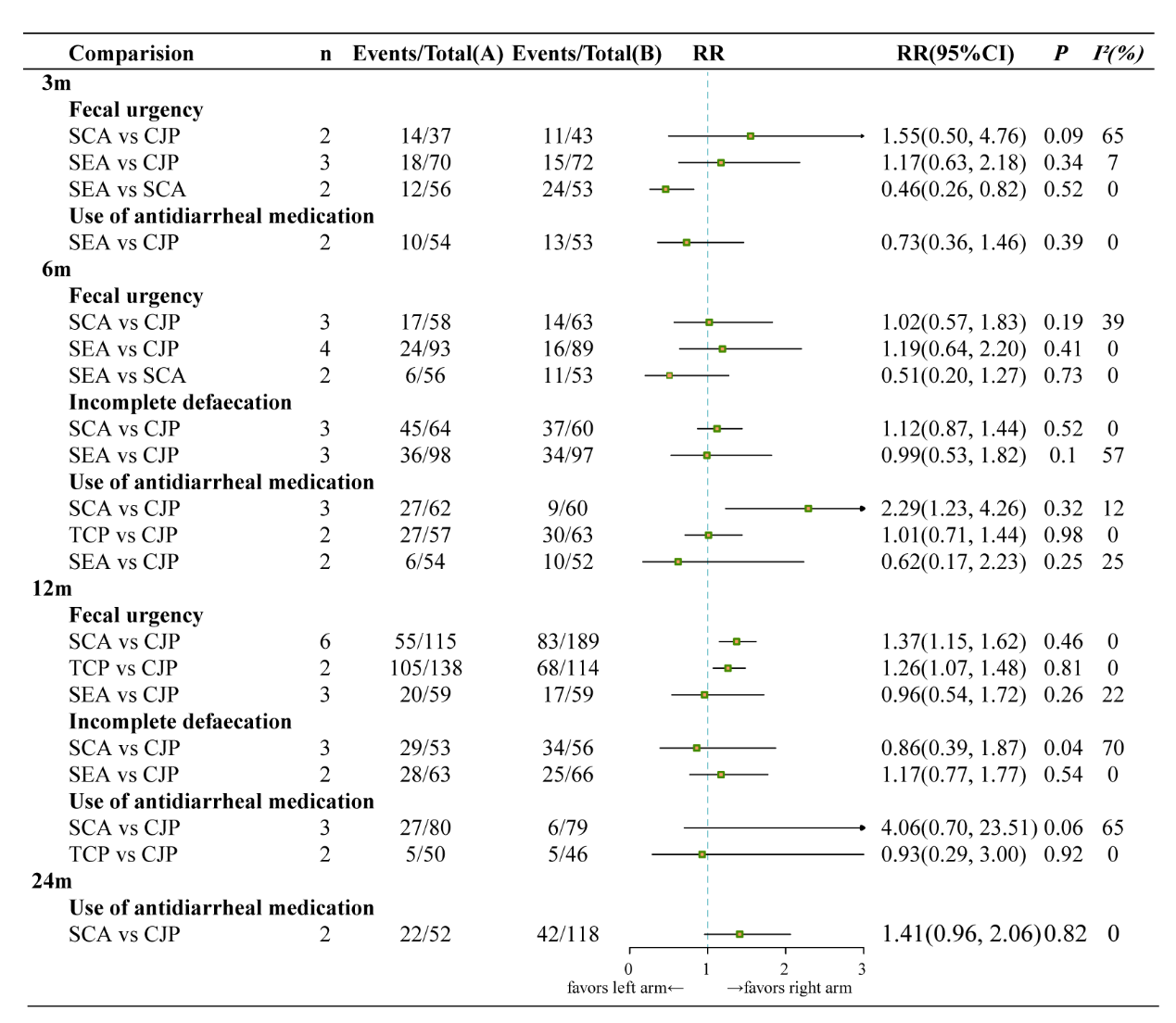


n, number of randomized trials included. CJP, colon J-pouch; SCA, straight colorectal anastomosis; TCP, transverse coloplasty; SEA, side-to-end anastomosis.

**Supplementary Table 8A** Relative effects table for postoperative anastomotic leakage.

| **CJP** | 1.44  (0.77, 2.76) | 2.11  (0.87, 5.53) | 0.52  (0.22, 1.07) |
| --- | --- | --- | --- |
|  | **SCA** | 1.45  (0.53, 4.30) | **0.36**  **(0.14, 0.82)** |
|  |  | **TCP** | **0.24**  **(0.06, 0.74)** |
|  |  |  | **SEA** |

Estimates are presented as risk ratio with a 95% confidence interval. Each cell gives the effect of the column-defining intervention relative to the row-defining intervention. The statistically significant results are indicated in bold. CJP, colon J-pouch; SCA, straight colorectal anastomosis; TCP, transverse coloplasty; SEA, side-to-end anastomosis.

**Supplementary Table 8B** Rank probabilities for postoperative anastomotic leakage.

|  | **Rank 1** | **Rank 2** | **Rank 3** | **Rank 4** | **SUCRA** |
| --- | --- | --- | --- | --- | --- |
| **CJP** | 0.031 | 0.825 | 0.135 | 0.009 | 0.628 |
| **SCA** | 0.006 | 0.100 | 0.678 | 0.216 | 0.299 |
| **TCP** | 0.006 | 0.040 | 0.180 | 0.774 | 0.092 |
| **SEA** | 0.957 | 0.035 | 0.007 | 0.001 | 0.982 |

CJP, colon J-pouch; SCA, straight colorectal anastomosis; TCP, transverse coloplasty; SEA, side-to-end anastomosis.

**Supplementary Fig. 5B** Comparison-adjusted funnel plot for postoperative anastomotic leakage.


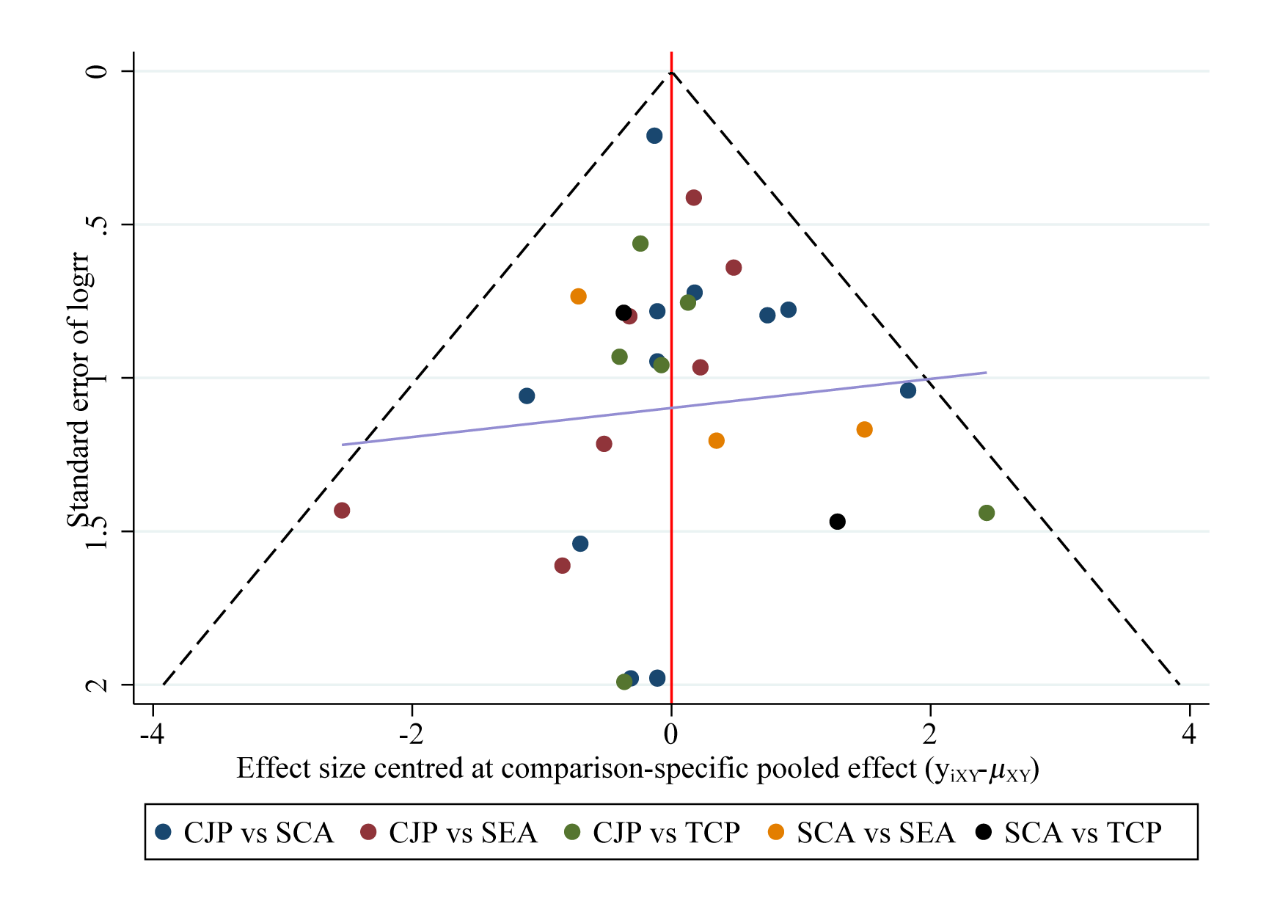
The red line shows the null hypothesis. Each point represents a direct comparison; different colours correspond to different comparisons. The dashed black line represents the 95% confidence interval. The horizontal line represents the regression line; the purple regression line demonstrates that no asymmetry is present. CJP, colon J-pouch; SCA, straight colorectal anastomosis; TCP, transverse coloplasty; SEA, side-to-end anastomosis.

**Supplementary Fig. 6A** Network plot for postoperative anastomotic stricture.


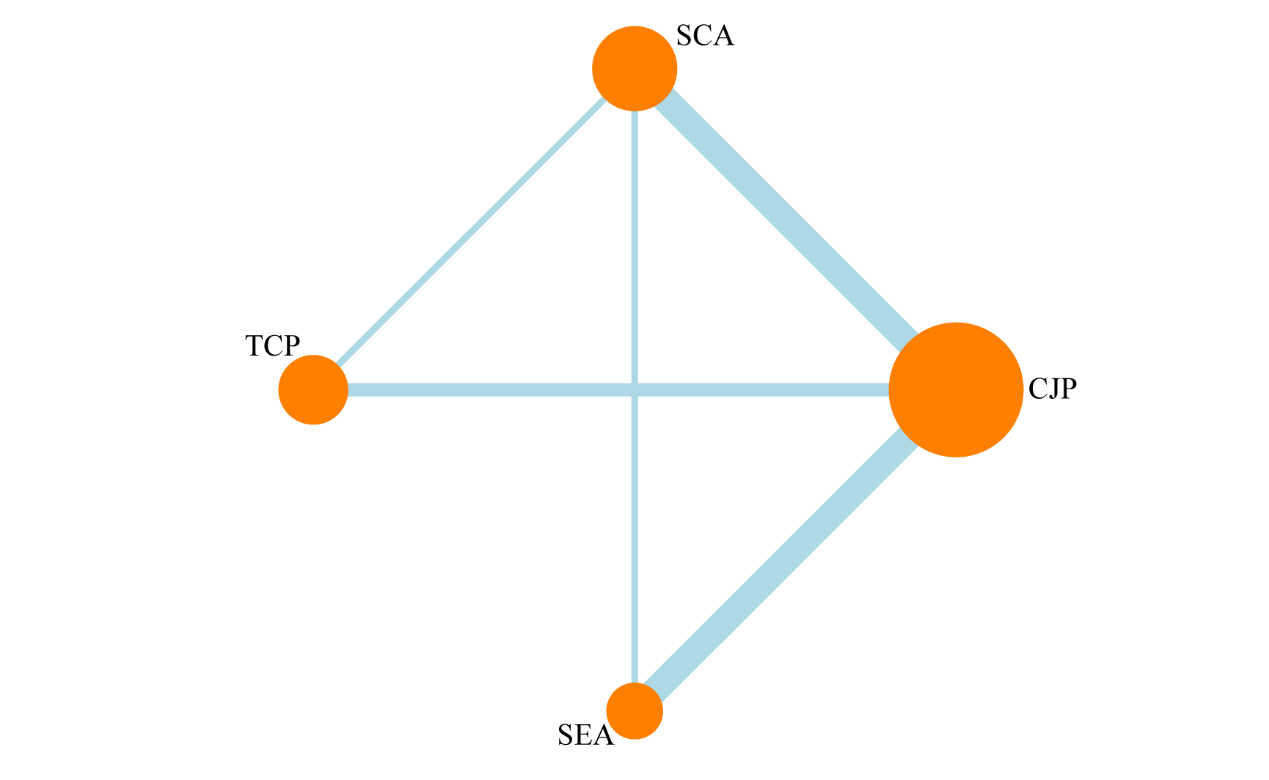


Circles represent interventions and their size is proportional to the number of patients who received the corresponding intervention. Lines represent direct comparisons, and their width is proportional to the number of studies in the corresponding comparison. CJP, colon J-pouch; SCA, straight colorectal anastomosis; TCP, transverse coloplasty; SEA, side-to-end anastomosis.

**Supplementary Table 9A** Relative effects table for postoperative anastomotic stricture.

| **CJP** | 1.37  (0.43, 3.86) | 1.05  (0.22, 4.59) | 1.23  (0.31, 4.77) |
| --- | --- | --- | --- |
|  | **SCA** | 0.76  (0.16, 4.04) | 0.90  (0.19, 4.56) |
|  |  | **TCP** | 1.18  (0.17, 8.92) |
|  |  |  | **SEA** |

Estimates are presented as risk ratio with a 95% confidence interval. Each cell gives the effect of the column-defining intervention relative to the row-defining intervention. The statistically significant results are indicated in bold. CJP, colon J-pouch; SCA, straight colorectal anastomosis; TCP, transverse coloplasty; SEA, side-to-end anastomosis.

**Supplementary Table 9B** Rank probabilities for postoperative anastomotic stricture.

|  | **Rank 1** | **Rank 2** | **Rank 3** | **Rank 4** | **SUCRA** |
| --- | --- | --- | --- | --- | --- |
| **CJP** | 0.289 | 0.394 | 0.239 | 0.078 | 0.632 |
| **SCA** | 0.113 | 0.193 | 0.329 | 0.365 | 0.351 |
| **TCP** | 0.355 | 0.218 | 0.204 | 0.223 | 0.568 |
| **SEA** | 0.243 | 0.195 | 0.228 | 0.334 | 0.449 |

CJP, colon J-pouch; SCA, straight colorectal anastomosis; TCP, transverse coloplasty; SEA, side-to-end anastomosis.

**Supplementary Fig. 6B** Comparison-adjusted funnel plot for postoperative anastomotic stricture.


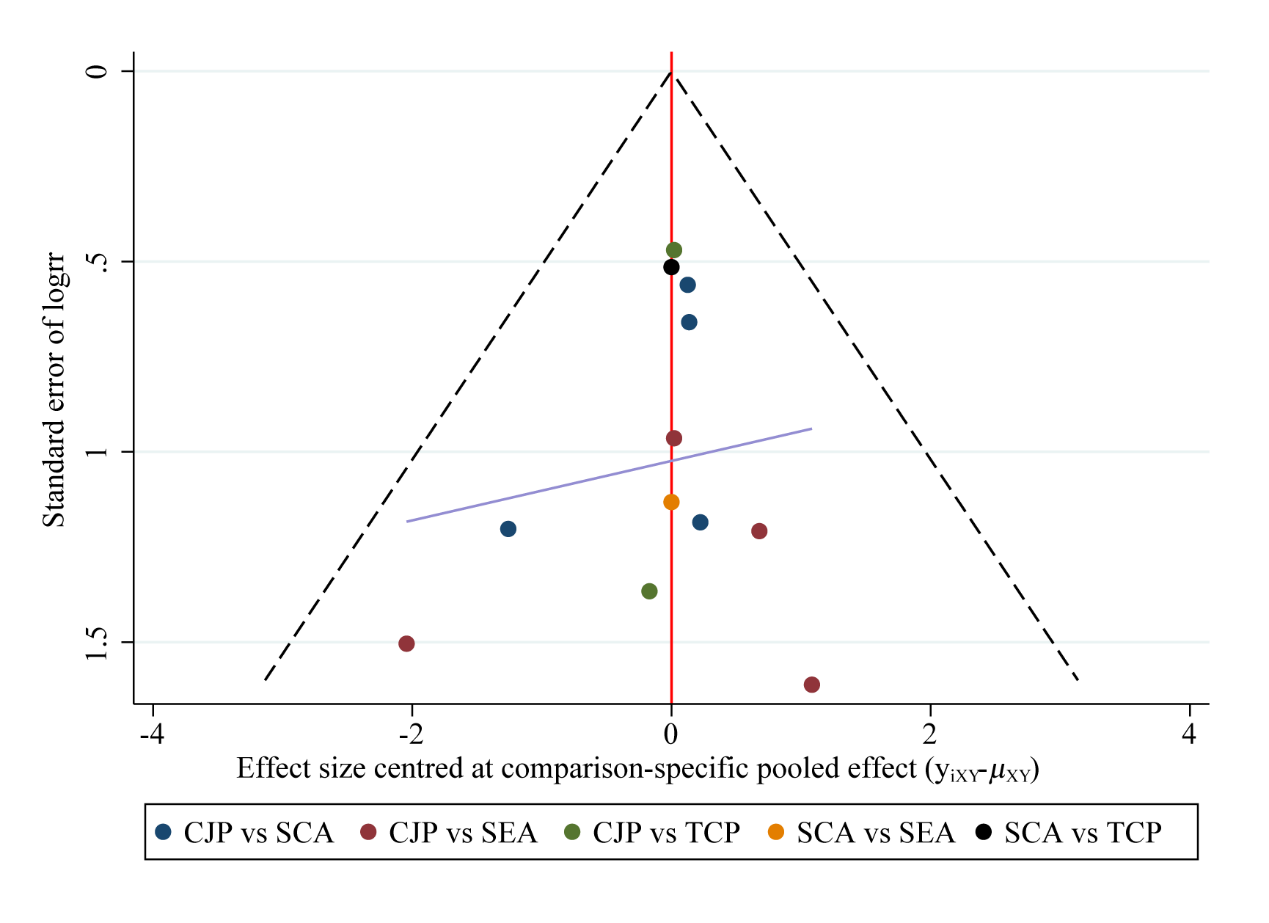
The red line shows the null hypothesis. Each point represents a direct comparison; different colours correspond to different comparisons. The dashed black line represents the 95% confidence interval. The horizontal line represents the regression line; the purple regression line demonstrates that asymmetry is present. CJP, colon J-pouch; SCA, straight colorectal anastomosis; TCP, transverse coloplasty; SEA, side-to-end anastomosis.

**Supplementary Fig. 7A** Network plot for postoperative reoperation.
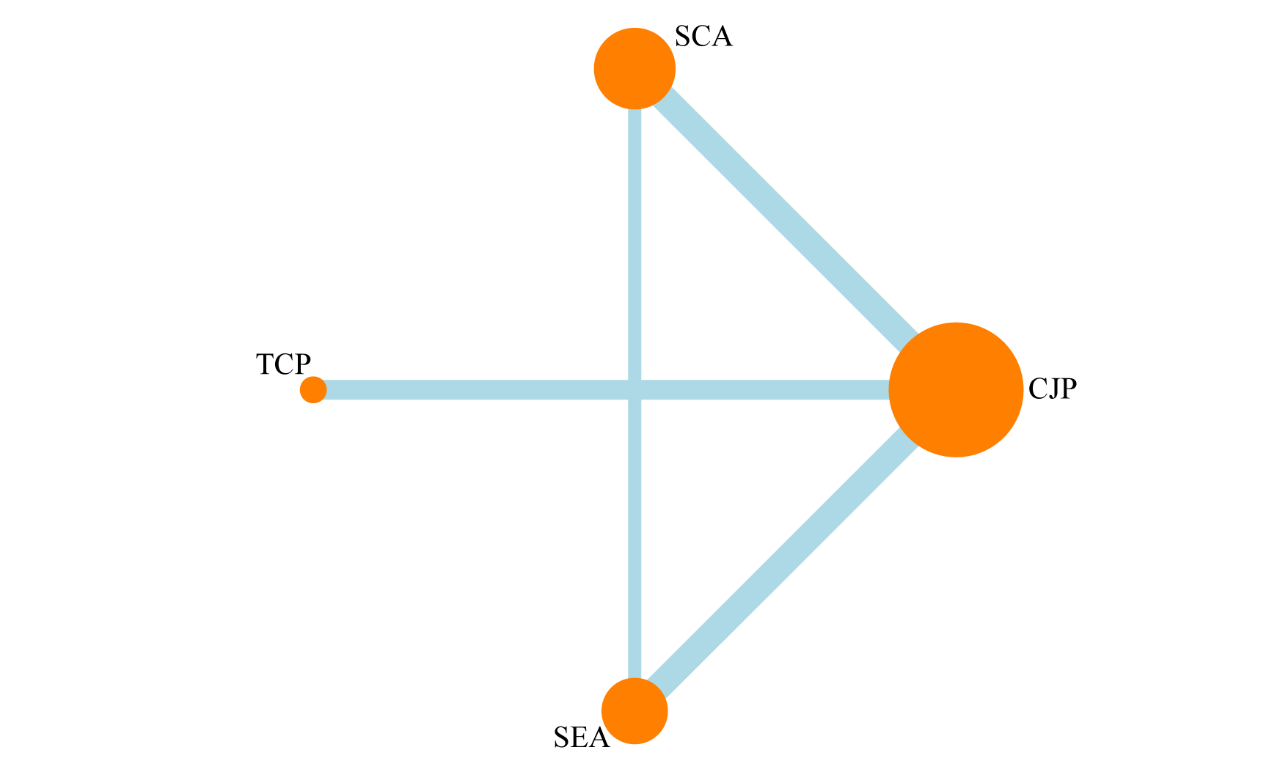


Circles represent interventions and their size is proportional to the number of patients who received the corresponding intervention. Lines represent direct comparisons, and their width is proportional to the number of studies in the corresponding comparison. CJP, colon J-pouch; SCA, straight colorectal anastomosis; TCP, transverse coloplasty; SEA, side-to-end anastomosis.

**Supplementary Table 10A** Relative effects table for postoperative reoperation.

| **CJP** | 1.38  (0.27, 5.58) | 4.98  (0.51, 74.24) | 0.42  (0.05, 1.50) |
| --- | --- | --- | --- |
|  | **SCA** | 3.64  (0.26, 91.33) | 0.31  (0.03, 1.61) |
|  |  | **TCP** | 0.08  (0.01, 1.01) |
|  |  |  | **SEA** |

Estimates are presented as risk ratio with a 95% confidence interval. Each cell gives the effect of the column-defining intervention relative to the row-defining intervention. The statistically significant results are indicated in bold. CJP, colon J-pouch; SCA, straight colorectal anastomosis; TCP, transverse coloplasty; SEA, side-to-end anastomosis.

**Supplementary Table 10B** Rank probabilities for postoperative reoperation.

|  | **Rank 1** | **Rank 2** | **Rank 3** | **Rank 4** | **SUCRA** |
| --- | --- | --- | --- | --- | --- |
| **CJP** | 0.052 | 0.610 | 0.312 | 0.026 | 0.563 |
| **SCA** | 0.052 | 0.255 | 0.555 | 0.138 | 0.407 |
| **TCP** | 0.019 | 0.046 | 0.104 | 0.832 | 0.084 |
| **SEA** | 0.877 | 0.089 | 0.030 | 0.005 | 0.946 |

CJP, colon J-pouch; SCA, straight colorectal anastomosis; TCP, transverse coloplasty; SEA, side-to-end anastomosis.

**Supplementary Fig. 7B** Comparison-adjusted funnel plot for postoperative reoperation.


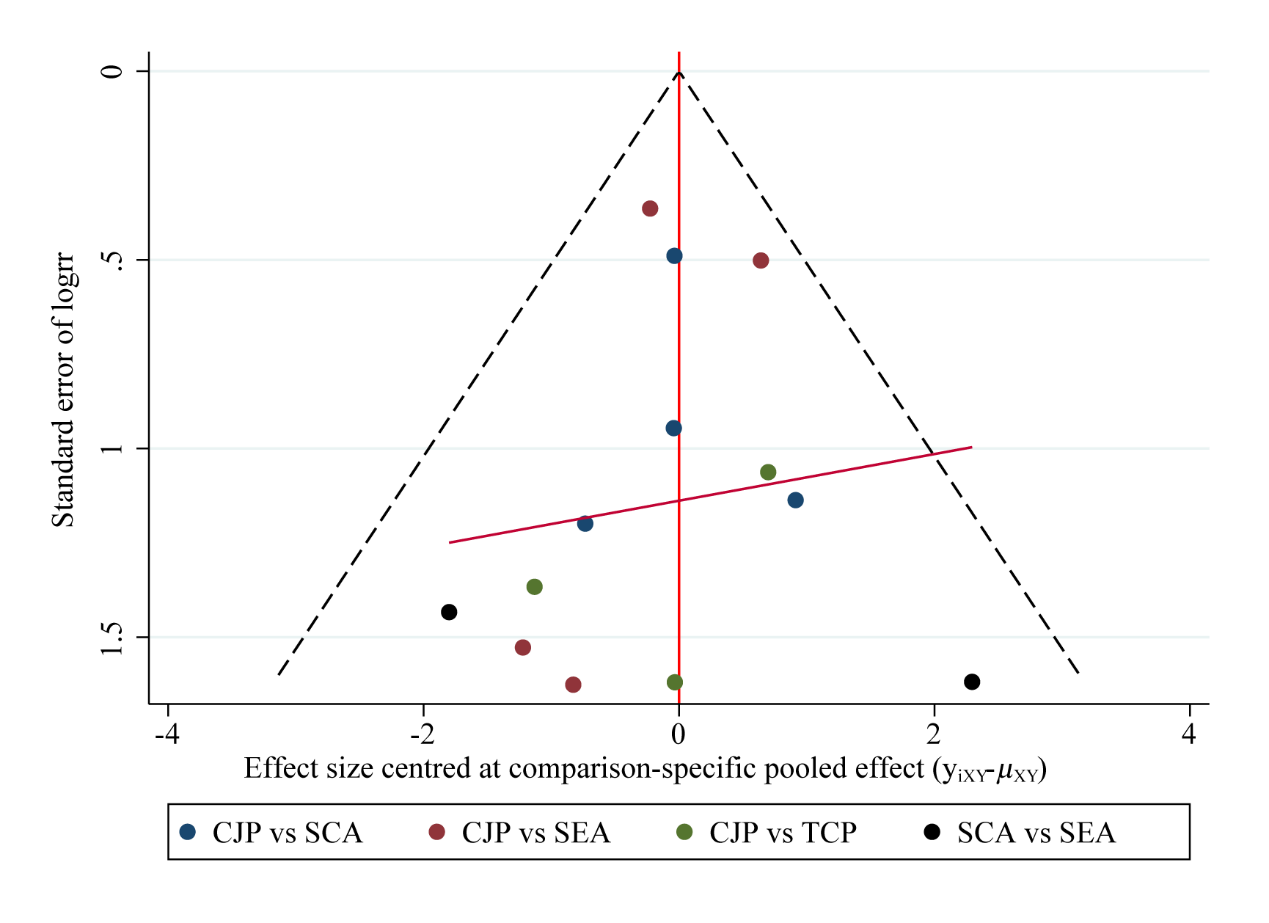
The red line shows the null hypothesis. Each point represents a direct comparison; different colours correspond to different comparisons. The dashed black line represents the 95% confidence interval. The horizontal line represents the regression line; the dark red regression line demonstrates that asymmetry is present. CJP, colon J-pouch; SCA, straight colorectal anastomosis; TCP, transverse coloplasty; SEA, side-to-end anastomosis.

**Supplementary Fig. 8A** Network plot for postoperative mortality within 30 days.


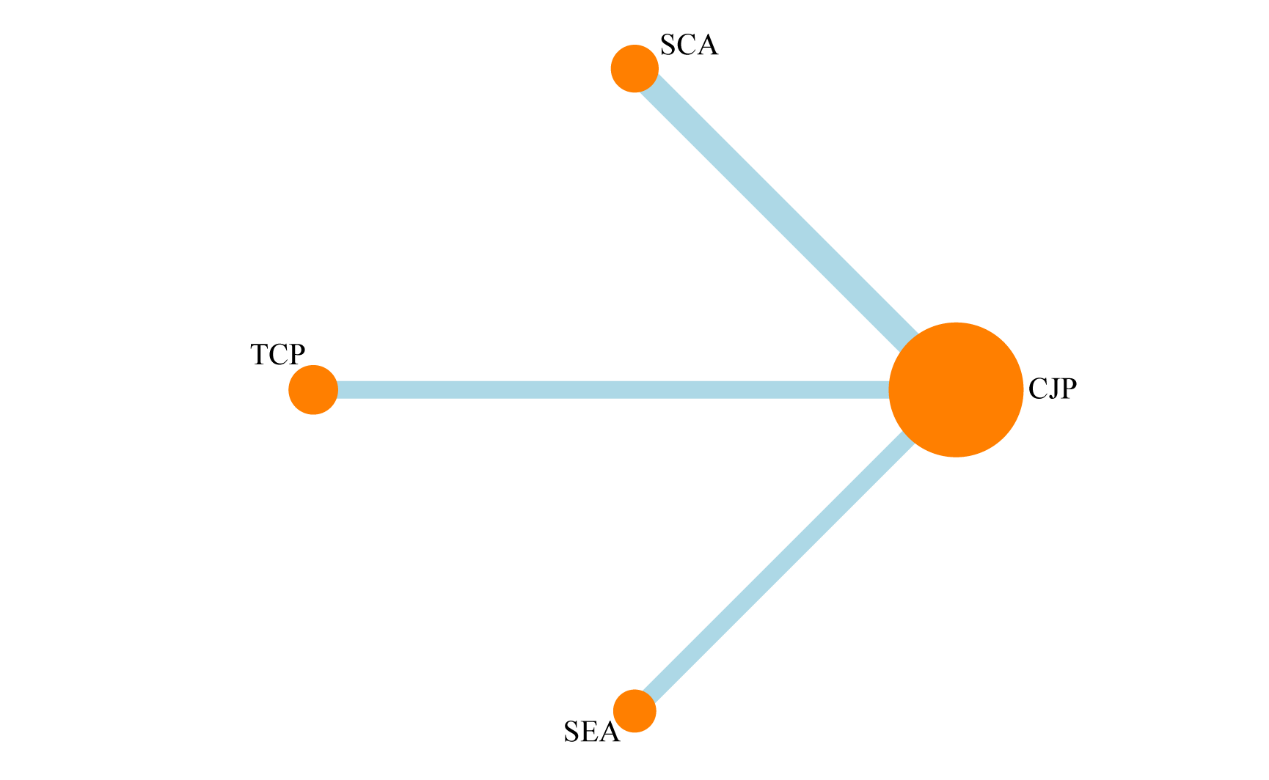


Circles represent interventions and their size is proportional to the number of patients who received the corresponding intervention. Lines represent direct comparisons, and their width is proportional to the number of studies in the corresponding comparison. CJP, colon J-pouch; SCA, straight colorectal anastomosis; TCP, transverse coloplasty; SEA, side-to-end anastomosis.

**Supplementary Table 11A** Relative effects table for postoperative mortality within 30 days.

| **CJP** | 0.27  (0.03, 1.54) | 2.35  (0.14, 74.92) | 0.16  (0.01, 1.74) |
| --- | --- | --- | --- |
|  | **SCA** | 9.87  (0.33, 565.50) | 0.58  (0.01, 16.46) |
|  |  | **TCP** | 0.06  (0.01, 2.89) |
|  |  |  | **SEA** |

Estimates are presented as risk ratio with a 95% confidence interval. Each cell gives the effect of the column-defining intervention relative to the row-defining intervention. The statistically significant results are indicated in bold. CJP, colon J-pouch; SCA, straight colorectal anastomosis; TCP, transverse coloplasty; SEA, side-to-end anastomosis.

**Supplementary Table 11B** Rank probabilities for postoperative mortality within 30 days.

|  | **Rank 1** | **Rank 2** | **Rank 3** | **Rank 4** | **SUCRA** |
| --- | --- | --- | --- | --- | --- |
| **CJP** | 0.004 | 0.096 | 0.673 | 0.226 | 0.293 |
| **SCA** | 0.355 | 0.527 | 0.093 | 0.025 | 0.738 |
| **TCP** | 0.036 | 0.083 | 0.158 | 0.724 | 0.143 |
| **SEA** | 0.605 | 0.294 | 0.076 | 0.025 | 0.826 |

CJP, colon J-pouch; SCA, straight colorectal anastomosis; TCP, transverse coloplasty; SEA, side-to-end anastomosis.

**Supplementary Fig. 8B** Comparison-adjusted funnel plot for postoperative mortality within 30 days.


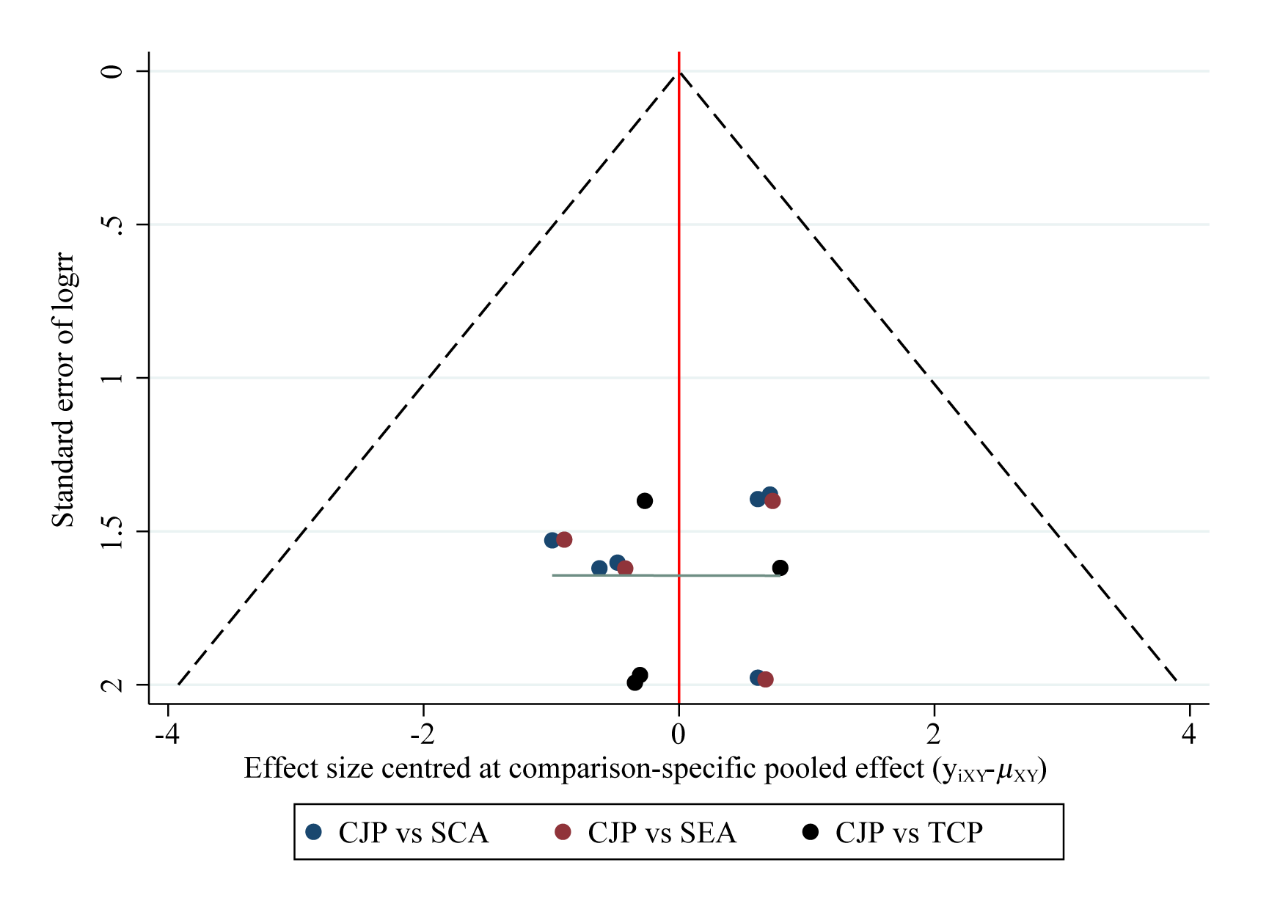


The red line shows the null hypothesis. Each point represents a direct comparison; different colours correspond to different comparisons. The dashed black line represents the 95% confidence interval. The horizontal line represents the regression line; the dark regression line demonstrates that no asymmetry is present. CJP, colon J-pouch; SCA, straight colorectal anastomosis; TCP, transverse coloplasty; SEA, side-to-end anastomosis.

**Supplementary Fig. 9A** Network plot for defecation frequency at 3 months postoperatively.
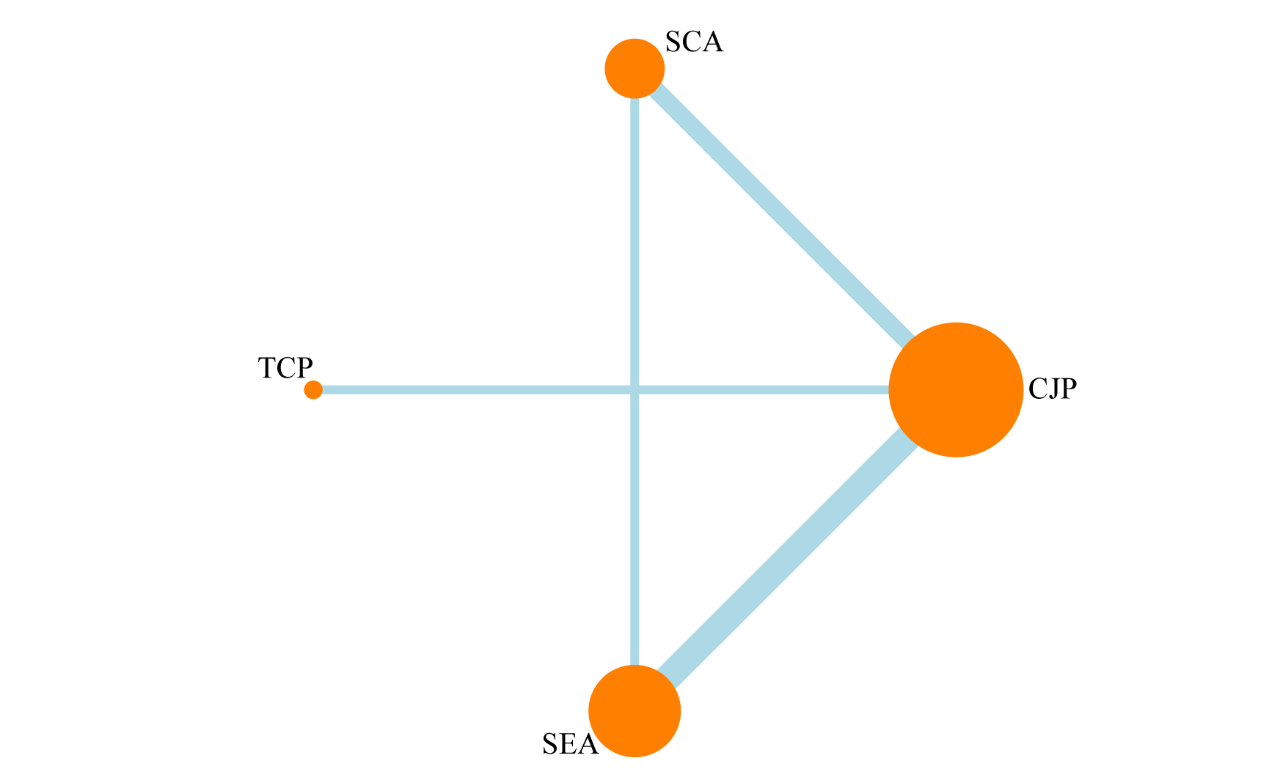


Circles represent interventions and their size is proportional to the number of patients who received the corresponding intervention. Lines represent direct comparisons, and their width is proportional to the number of studies in the corresponding comparison. CJP, colon J-pouch; SCA, straight colorectal anastomosis; TCP, transverse coloplasty; SEA, side-to-end anastomosis.

**Supplementary Table 12B** Rank probabilities for defecation frequency at 3 months postoperatively.

|  | **Rank 1** | **Rank 2** | **Rank 3** | **Rank 4** | **SUCRA** |
| --- | --- | --- | --- | --- | --- |
| **CJP** | 0.315 | 0.545 | 0.123 | 0.016 | 0.720 |
| **SCA** | 0.042 | 0.094 | 0.258 | 0.607 | 0.190 |
| **TCP** | 0.571 | 0.168 | 0.131 | 0.130 | 0.727 |
| **SEA** | 0.072 | 0.193 | 0.488 | 0.247 | 0.363 |

CJP, colon J-pouch; SCA, straight colorectal anastomosis; TCP, transverse coloplasty; SEA, side-to-end anastomosis.

**Supplementary Fig. 9B** Comparison-adjusted funnel plot for defecation frequency at 3 months postoperatively.


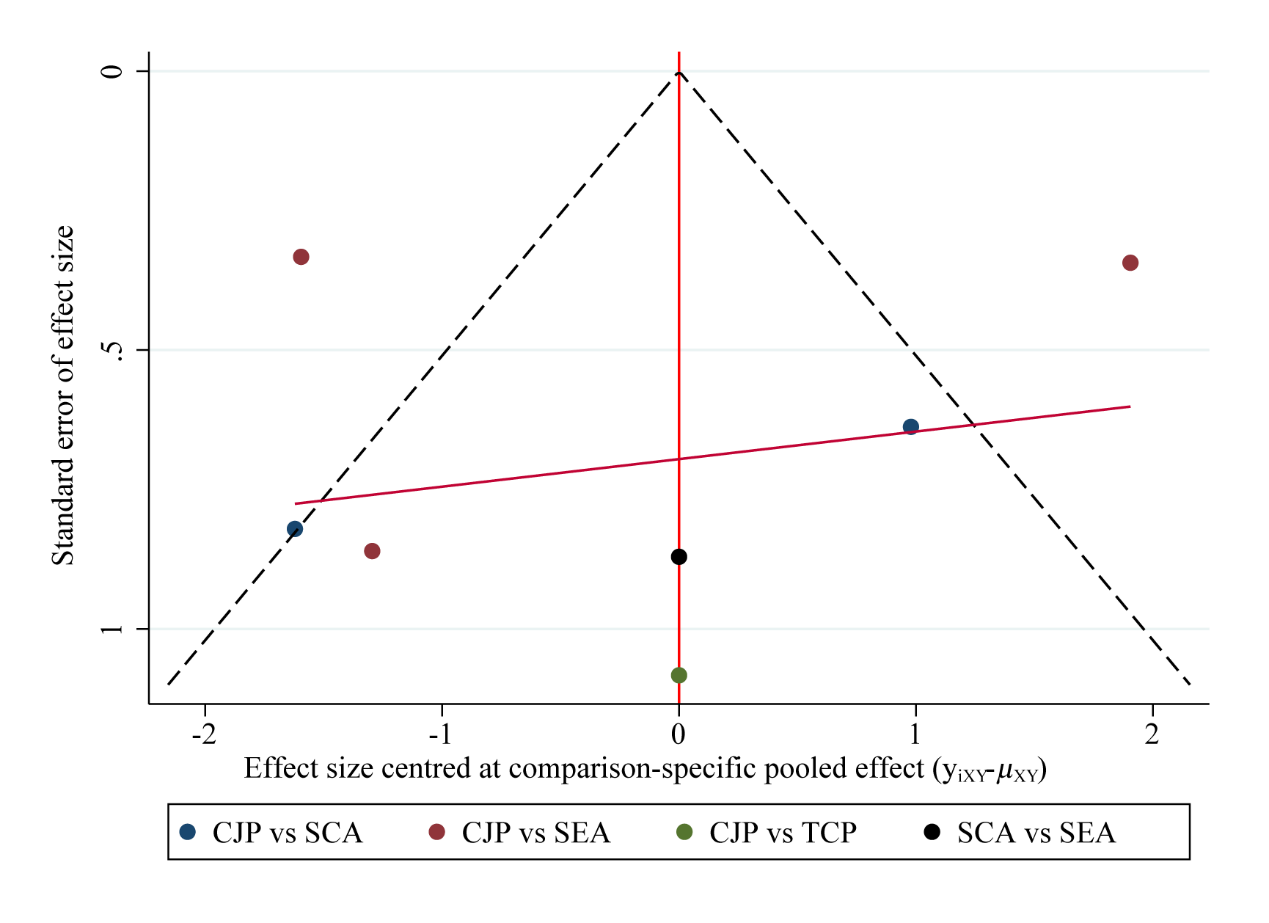
The red line shows the null hypothesis. Each point represents a direct comparison; different colours correspond to different comparisons. The dashed black line represents the 95% confidence interval. The horizontal line represents the regression line; the dark red regression line demonstrates that asymmetry is present. CJP, colon J-pouch; SCA, straight colorectal anastomosis; TCP, transverse coloplasty; SEA, side-to-end anastomosis.

**Supplementary Fig. 10A** Network plot for fecal urgency at 3 months postoperatively.
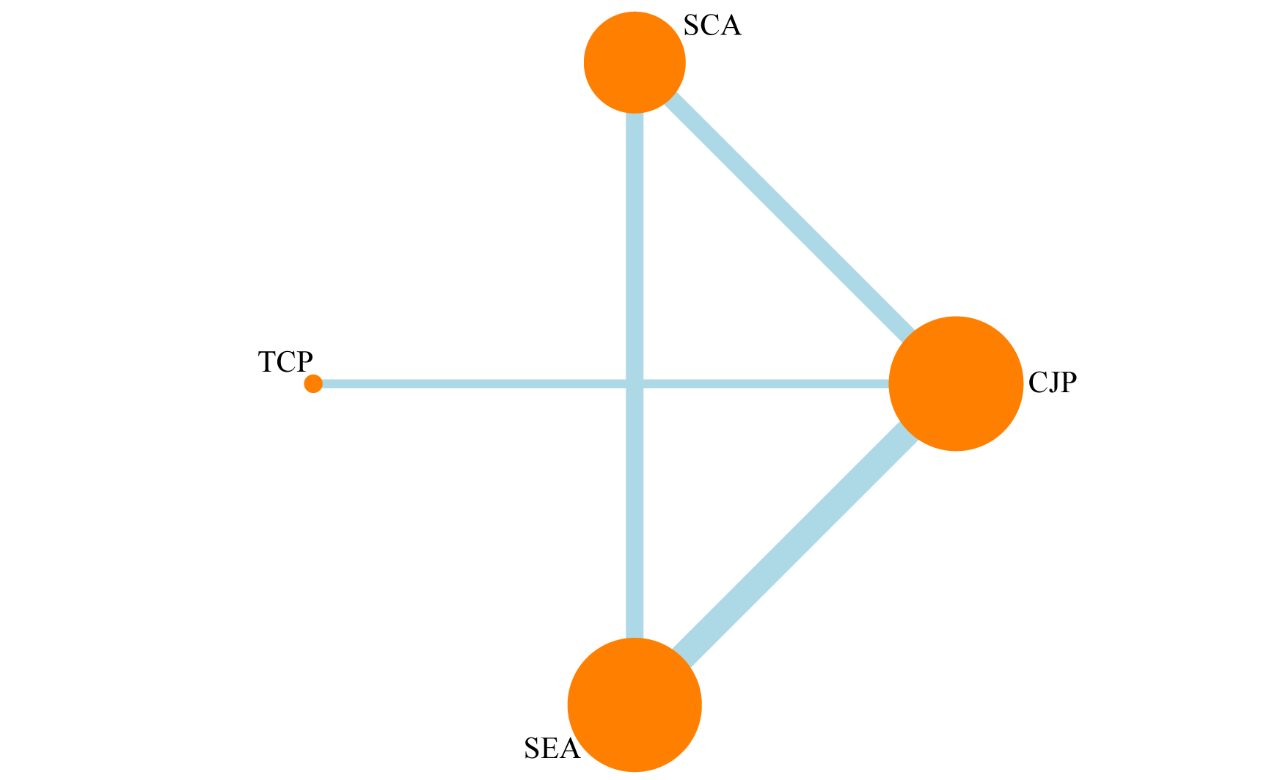
Circles represent interventions and their size is proportional to the number of patients who received the corresponding intervention. Lines represent direct comparisons, and their width is proportional to the number of studies in the corresponding comparison. CJP, colon J-pouch; SCA, straight colorectal anastomosis; TCP, transverse coloplasty; SEA, side-to-end anastomosis.

**Supplementary Table 13A** Relative effects table for fecal urgency at 3 months postoperatively.

| **CJP** | 1.92  (0.81, 4.39) | 0.74  (0.12, 4.06) | 1.07  (0.50, 2.30) |
| --- | --- | --- | --- |
|  | **SCA** | 0.39  (0.05, 2.64) | 0.56  (0.25, 1.28) |
|  |  | **TCP** | 1.45  (0.23, 10.23) |
|  |  |  | **SEA** |

Estimates are presented as risk ratio with a 95% confidence interval. Each cell gives the effect of the column-defining intervention relative to the row-defining intervention. The statistically significant results are indicated in bold. CJP, colon J-pouch; SCA, straight colorectal anastomosis; TCP, transverse coloplasty; SEA, side-to-end anastomosis.

**Supplementary Table 13B** Rank probabilities for fecal urgency at 3 months postoperatively.

|  | **Rank 1** | **Rank 2** | **Rank 3** | **Rank 4** | **SUCRA** |
| --- | --- | --- | --- | --- | --- |
| **CJP** | 0.205 | 0.497 | 0.269 | 0.029 | 0.626 |
| **SCA** | 0.013 | 0.038 | 0.167 | 0.782 | 0.094 |
| **TCP** | 0.588 | 0.117 | 0.154 | 0.141 | 0.717 |
| **SEA** | 0.194 | 0.348 | 0.410 | 0.048 | 0.563 |

CJP, colon J-pouch; SCA, straight colorectal anastomosis; TCP, transverse coloplasty; SEA, side-to-end anastomosis.

**Supplementary Fig. 10B** Comparison-adjusted funnel plot for fecal urgency at 3 months postoperatively.


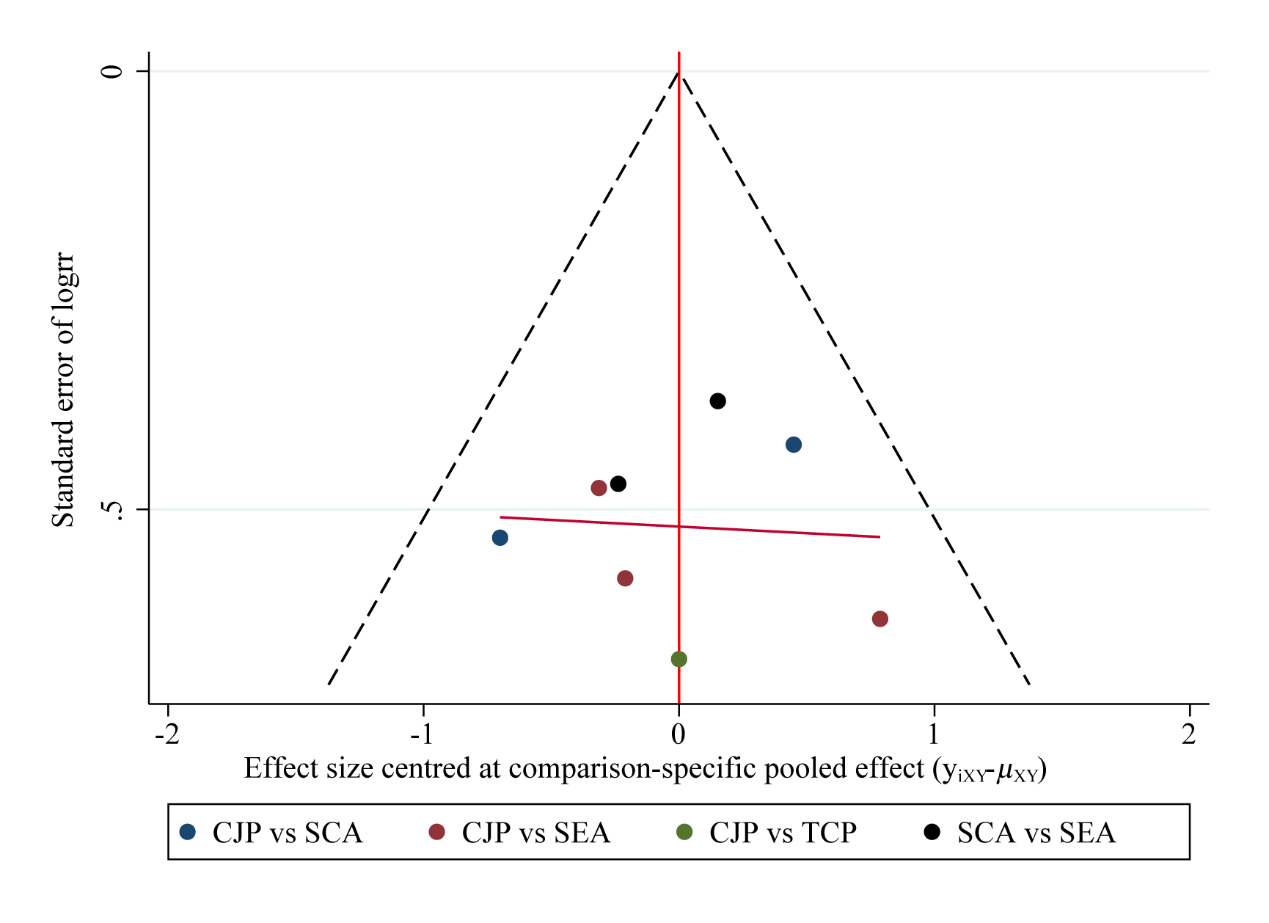
The red line shows the null hypothesis. Each point represents a direct comparison; different colours correspond to different comparisons. The dashed black line represents the 95% confidence interval. The horizontal line represents the regression line; the dark red regression line demonstrates that no asymmetry is present. CJP, colon J-pouch; SCA, straight colorectal anastomosis; TCP, transverse coloplasty; SEA, side-to-end anastomosis.

**Supplementary Fig. 11A** Network plot for use of antidiarrheal medication at 3 months postoperatively.


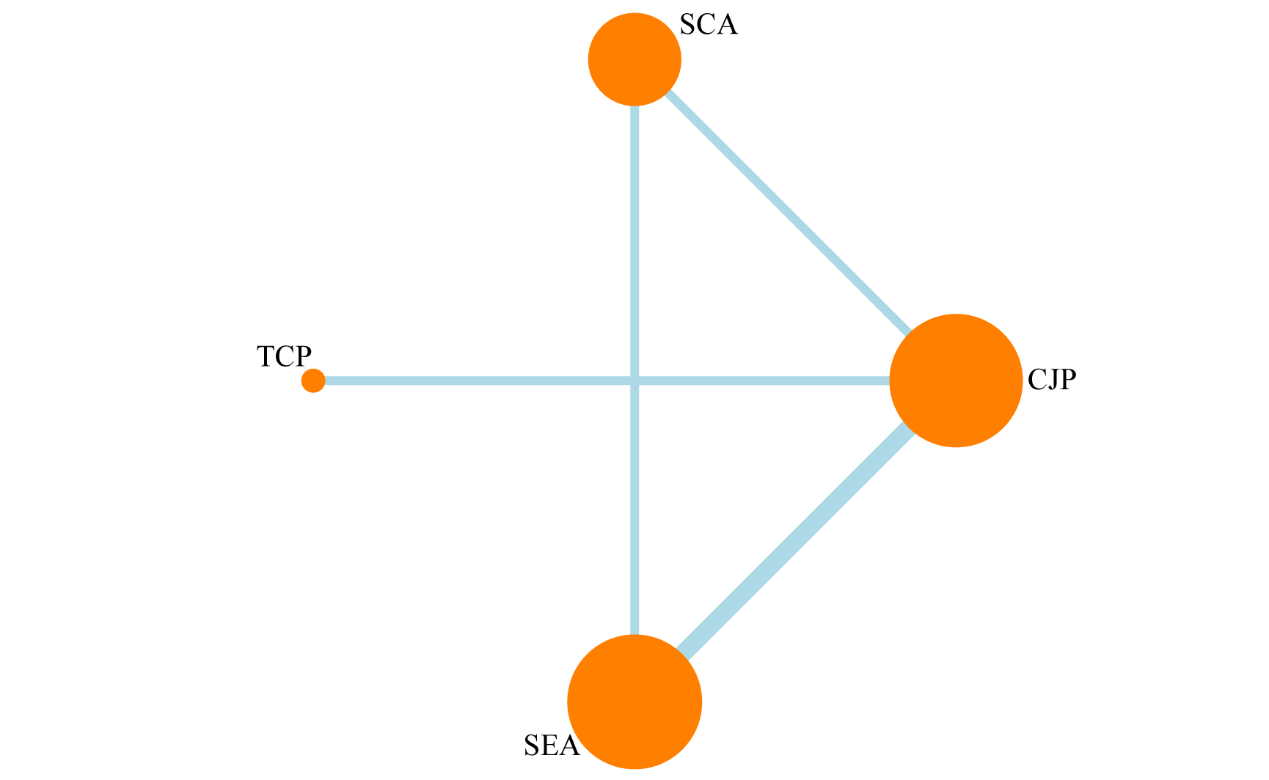


Circles represent interventions and their size is proportional to the number of patients who received the corresponding intervention. Lines represent direct comparisons, and their width is proportional to the number of studies in the corresponding comparison. CJP, colon J-pouch; SCA, straight colorectal anastomosis; TCP, transverse coloplasty; SEA, side-to-end anastomosis.

**Supplementary Table 14A** Relative effects table for use of antidiarrheal medication at 3 months postoperatively.

| **CJP** | 2.16  (0.82, 5.76) | 0.72  (0.12, 3.91) | 0.93  (0.39, 2.38) |
| --- | --- | --- | --- |
|  | **SCA** | 0.33  (0.04, 2.38) | 0.43  (0.16, 1.25) |
|  |  | **TCP** | 1.3  (0.19, 9.7) |
|  |  |  | **SEA** |

Estimates are presented as risk ratio with a 95% confidence interval. Each cell gives the effect of the column-defining intervention relative to the row-defining intervention. The statistically significant results are indicated in bold. CJP, colon J-pouch; SCA, straight colorectal anastomosis; TCP, transverse coloplasty; SEA, side-to-end anastomosis.

**Supplementary Table 14B** Rank probabilities for use of antidiarrheal medication at 3 months postoperatively.

|  | **Rank 1** | **Rank 2** | **Rank 3** | **Rank 4** | **SUCRA** |
| --- | --- | --- | --- | --- | --- |
| **CJP** | 0.146 | 0.462 | 0.367 | 0.025 | 0.576 |
| **SCA** | 0.009 | 0.030 | 0.135 | 0.825 | 0.075 |
| **TCP** | 0.557 | 0.140 | 0.189 | 0.115 | 0.713 |
| **SEA** | 0.288 | 0.368 | 0.309 | 0.035 | 0.636 |

CJP, colon J-pouch; SCA, straight colorectal anastomosis; TCP, transverse coloplasty; SEA, side-to-end anastomosis.

**Supplementary Fig. 11B** Comparison-adjusted funnel plot for use of antidiarrheal medication at 3 months postoperatively.


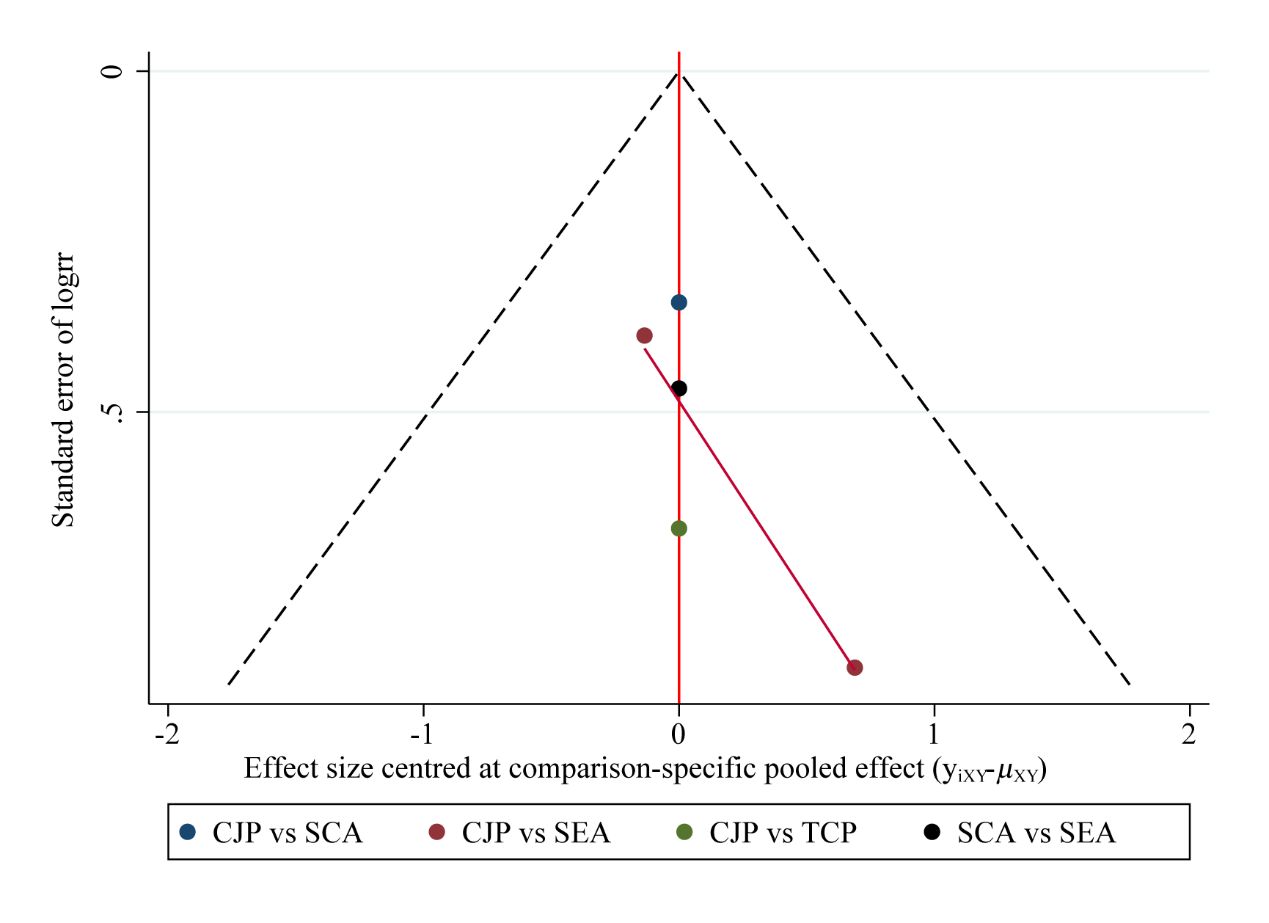
The red line shows the null hypothesis. Each point represents a direct comparison; different colours correspond to different comparisons. The dashed black line represents the 95% confidence interval. The horizontal line represents the regression line; the dark red regression line demonstrates that asymmetry is present. CJP, colon J-pouch; SCA, straight colorectal anastomosis; TCP, transverse coloplasty; SEA, side-to-end anastomosis.

**Supplementary Fig. 12A** Network plot for defecation frequency at 6 months postoperatively.


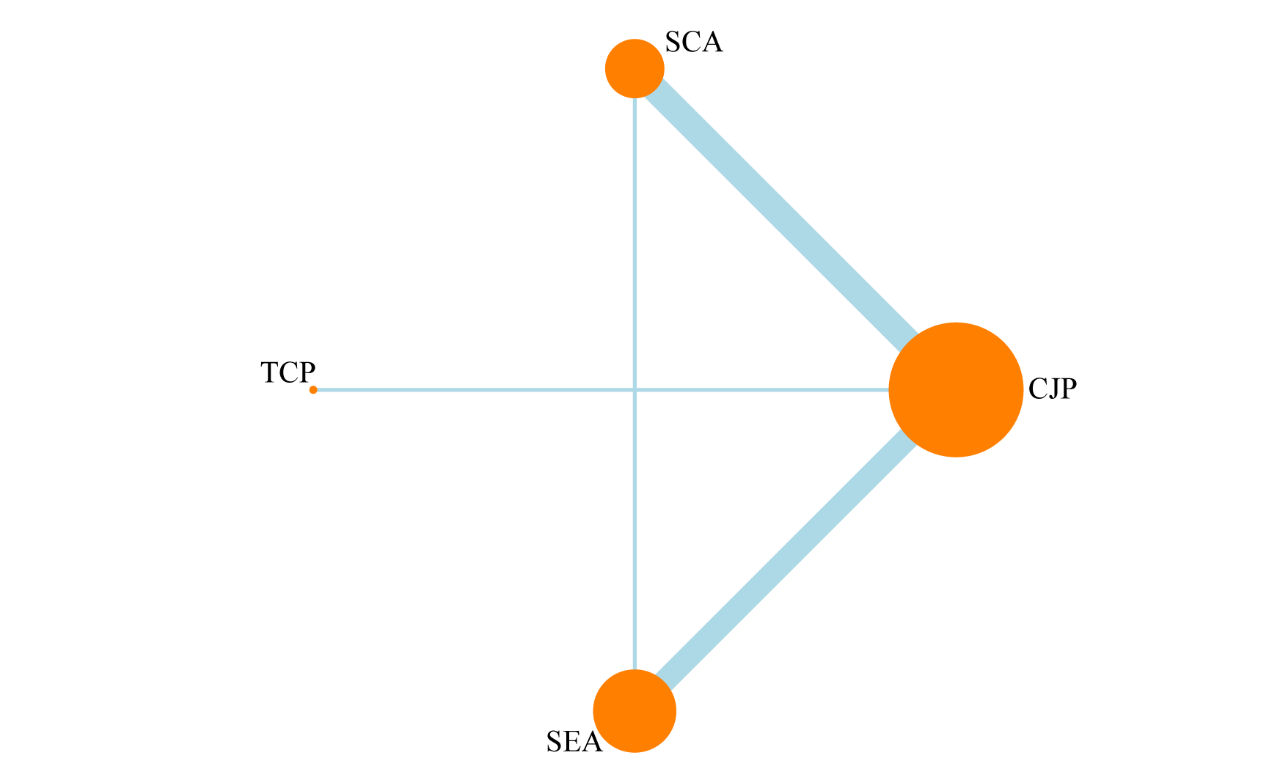
Circles represent interventions and their size is proportional to the number of patients who received the corresponding intervention. Lines represent direct comparisons, and their width is proportional to the number of studies in the corresponding comparison. CJP, colon J-pouch; SCA, straight colorectal anastomosis; TCP, transverse coloplasty; SEA, side-to-end anastomosis.

**Supplementary Table 15B** Rank probabilities for defecation frequency at 6 months postoperatively.

|  | **Rank 1** | **Rank 2** | **Rank 3** | **Rank 4** | **SUCRA** |
| --- | --- | --- | --- | --- | --- |
| **CJP** | 0.335 | 0.496 | 0.169 | 0.001 | 0.722 |
| **SCA** | 0.001 | 0.001 | 0.097 | 0.900 | 0.033 |
| **TCP** | 0.330 | 0.103 | 0.469 | 0.097 | 0.556 |
| **SEA** | 0.334 | 0.400 | 0.265 | 0.002 | 0.689 |

CJP, colon J-pouch; SCA, straight colorectal anastomosis; TCP, transverse coloplasty; SEA, side-to-end anastomosis.

**Supplementary Fig. 12B** Comparison-adjusted funnel plot for defecation frequency at 6 months postoperatively.


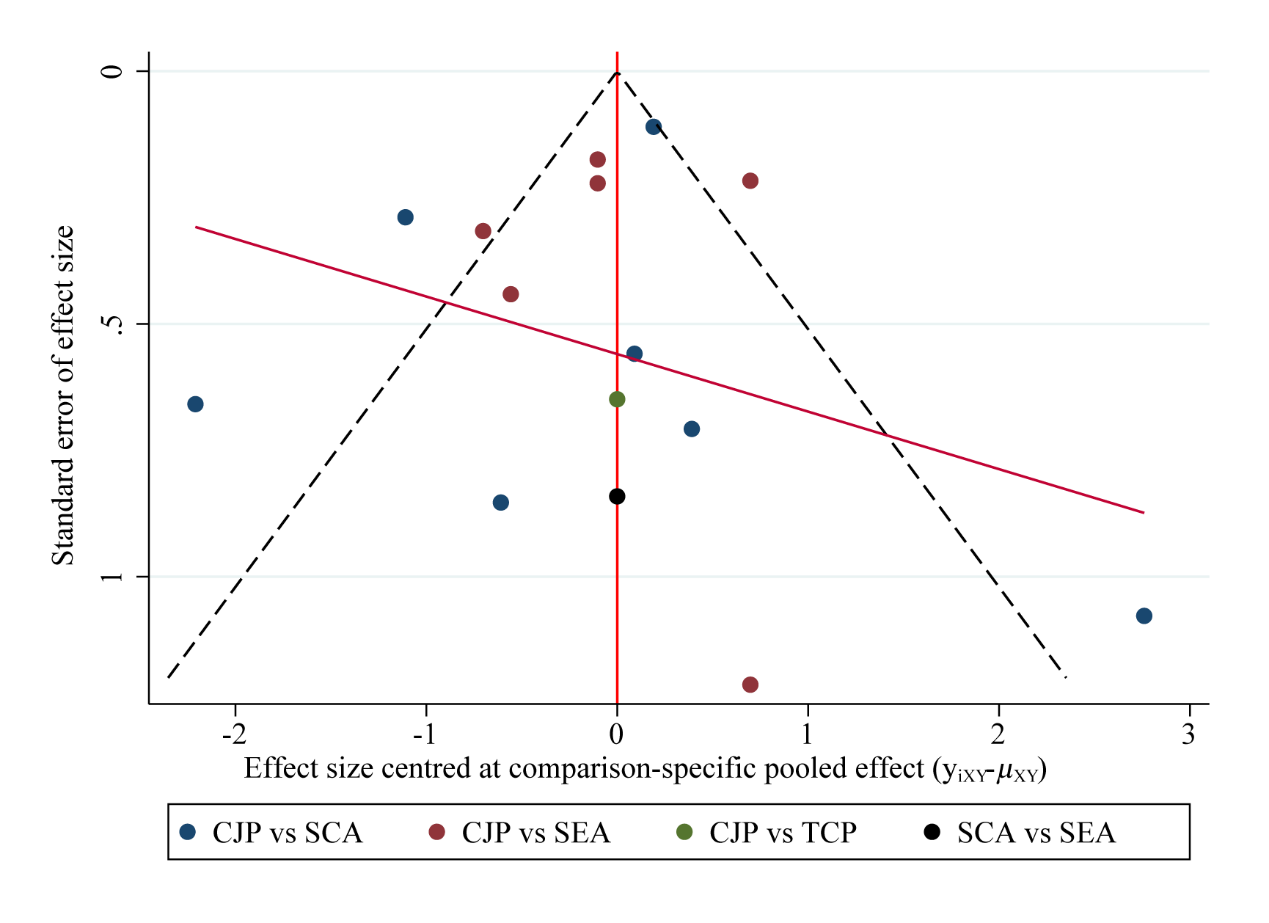
The red line shows the null hypothesis. Each point represents a direct comparison; different colours correspond to different comparisons. The dashed black line represents the 95% confidence interval. The horizontal line represents the regression line; the dark red regression line demonstrates that asymmetry is present. CJP, colon J-pouch; SCA, straight colorectal anastomosis; TCP, transverse coloplasty; SEA, side-to-end anastomosis.

**Supplementary Fig. 13A** Network plot for fecal urgency at 6 months postoperatively.


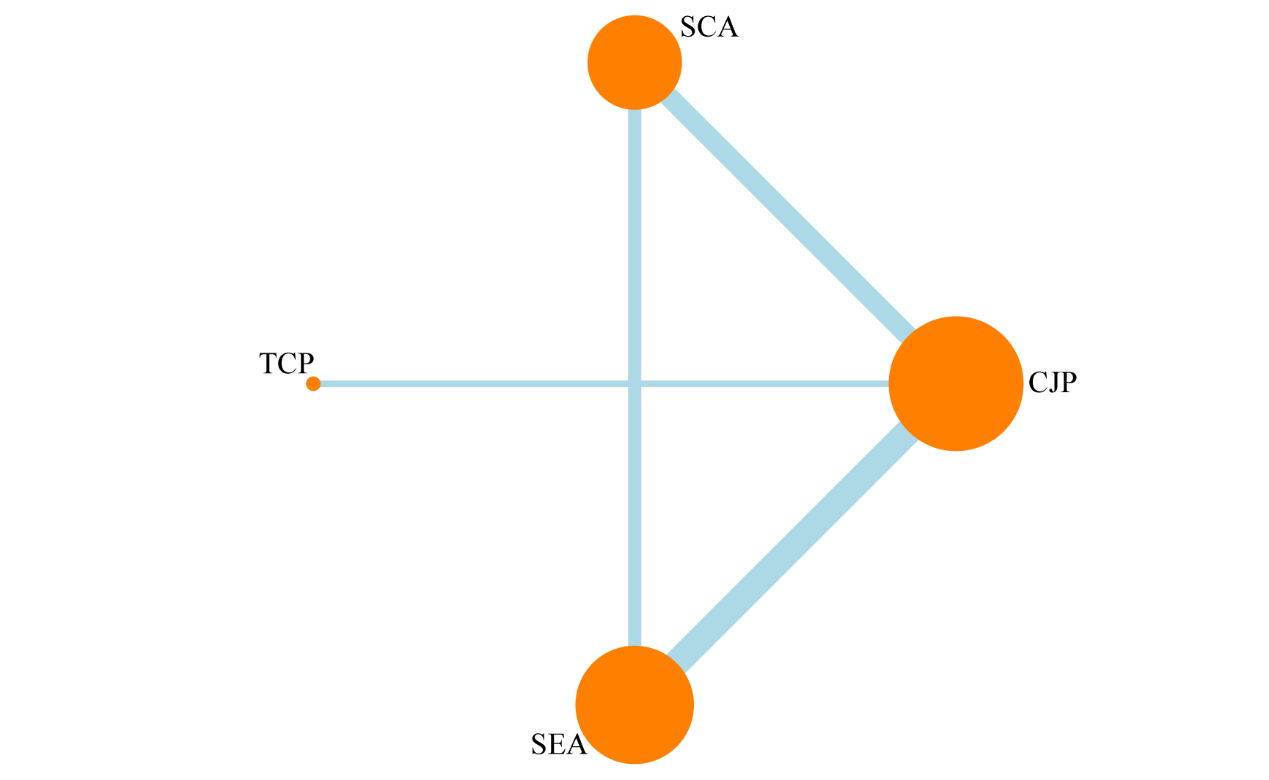
Circles represent interventions and their size is proportional to the number of patients who received the corresponding intervention. Lines represent direct comparisons, and their width is proportional to the number of studies in the corresponding comparison. CJP, colon J-pouch; SCA, straight colorectal anastomosis; TCP, transverse coloplasty; SEA, side-to-end anastomosis.

**Supplementary Table 16A** Relative effects table for fecal urgency at 6 months postoperatively.

| **CJP** | 1.48  (0.62, 5.75) | 0.73  (0.08, 6.47) | 1.14  (0.50, 3.94) |
| --- | --- | --- | --- |
|  | **SCA** | 0.49  (0.03, 4.54) | 0.77  (0.22, 2.41) |
|  |  | **TCP** | 1.58  (0.17, 22.4) |
|  |  |  | **SEA** |

Estimates are presented as risk ratio with a 95% confidence interval. Each cell gives the effect of the column-defining intervention relative to the row-defining intervention. The statistically significant results are indicated in bold. CJP, colon J-pouch; SCA, straight colorectal anastomosis; TCP, transverse coloplasty; SEA, side-to-end anastomosis.

**Supplementary Table 16B** Rank probabilities for fecal urgency at 6 months postoperatively.

|  | **Rank 1** | **Rank 2** | **Rank 3** | **Rank 4** | **SUCRA** |
| --- | --- | --- | --- | --- | --- |
| **CJP** | 0.207 | 0.481 | 0.253 | 0.059 | 0.612 |
| **SCA** | 0.057 | 0.127 | 0.275 | 0.542 | 0.232 |
| **TCP** | 0.583 | 0.106 | 0.104 | 0.206 | 0.689 |
| **SEA** | 0.153 | 0.286 | 0.368 | 0.193 | 0.466 |

CJP, colon J-pouch; SCA, straight colorectal anastomosis; TCP, transverse coloplasty; SEA, side-to-end anastomosis.

**Supplementary Fig. 13B** Comparison-adjusted funnel plot for fecal urgency at 6 months postoperatively.


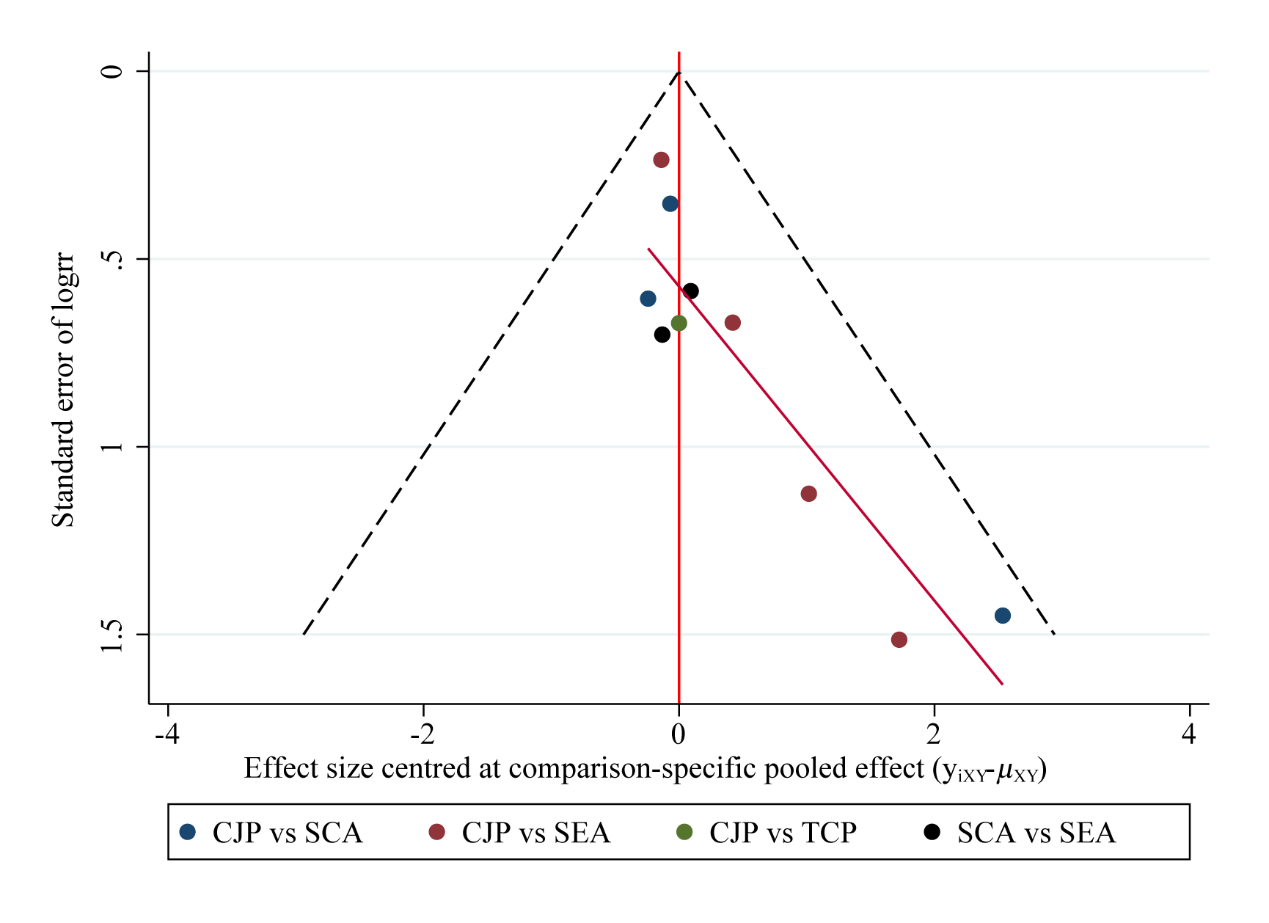
The red line shows the null hypothesis. Each point represents a direct comparison; different colours correspond to different comparisons. The dashed black line represents the 95% confidence interval. The horizontal line represents the regression line; the dark red regression line demonstrates that asymmetry is present. CJP, colon J-pouch; SCA, straight colorectal anastomosis; TCP, transverse coloplasty; SEA, side-to-end anastomosis.

**Supplementary Fig. 14A** Network plot for incomplete defecation at 6 months postoperatively.


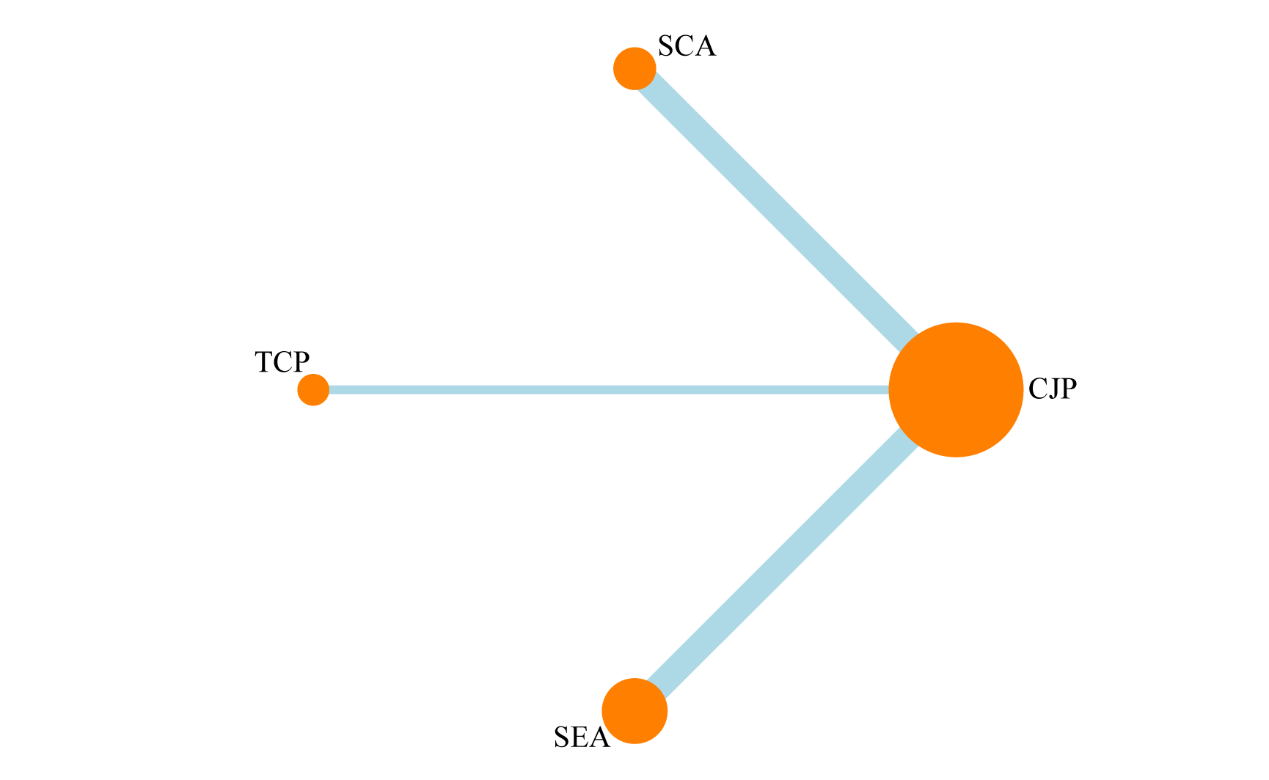
Circles represent interventions and their size is proportional to the number of patients who received the corresponding intervention. Lines represent direct comparisons, and their width is proportional to the number of studies in the corresponding comparison. CJP, colon J-pouch; SCA, straight colorectal anastomosis; TCP, transverse coloplasty; SEA, side-to-end anastomosis.

**Supplementary Table 17A** Relative effects table for incomplete defecation at 6 months postoperatively.

| **CJP** | 1.14  (0.67, 1.97) | 0.95  (0.4, 2.27) | 0.99  (0.5, 1.76) |
| --- | --- | --- | --- |
|  | **SCA** | 0.84  (0.3, 2.27) | 0.87  (0.36, 1.85) |
|  |  | **TCP** | 1.04  (0.34, 2.81) |
|  |  |  | **SEA** |

Estimates are presented as risk ratio with a 95% confidence interval. Each cell gives the effect of the column-defining intervention relative to the row-defining intervention. The statistically significant results are indicated in bold. CJP, colon J-pouch; SCA, straight colorectal anastomosis; TCP, transverse coloplasty; SEA, side-to-end anastomosis.

**Supplementary Table 17B** Rank probabilities for incomplete defecation at 6 months postoperatively.

|  | **Rank 1** | **Rank 2** | **Rank 3** | **Rank 4** | **SUCRA** |
| --- | --- | --- | --- | --- | --- |
| **CJP** | 0.153 | 0.417 | 0.350 | 0.080 | 0.548 |
| **SCA** | 0.110 | 0.160 | 0.257 | 0.472 | 0.303 |
| **TCP** | 0.416 | 0.195 | 0.172 | 0.218 | 0.603 |
| **SEA** | 0.321 | 0.228 | 0.221 | 0.230 | 0.547 |

CJP, colon J-pouch; SCA, straight colorectal anastomosis; TCP, transverse coloplasty; SEA, side-to-end anastomosis.

**Supplementary Fig. 14B** Comparison-adjusted funnel plot for incomplete defecation at 6 months postoperatively.


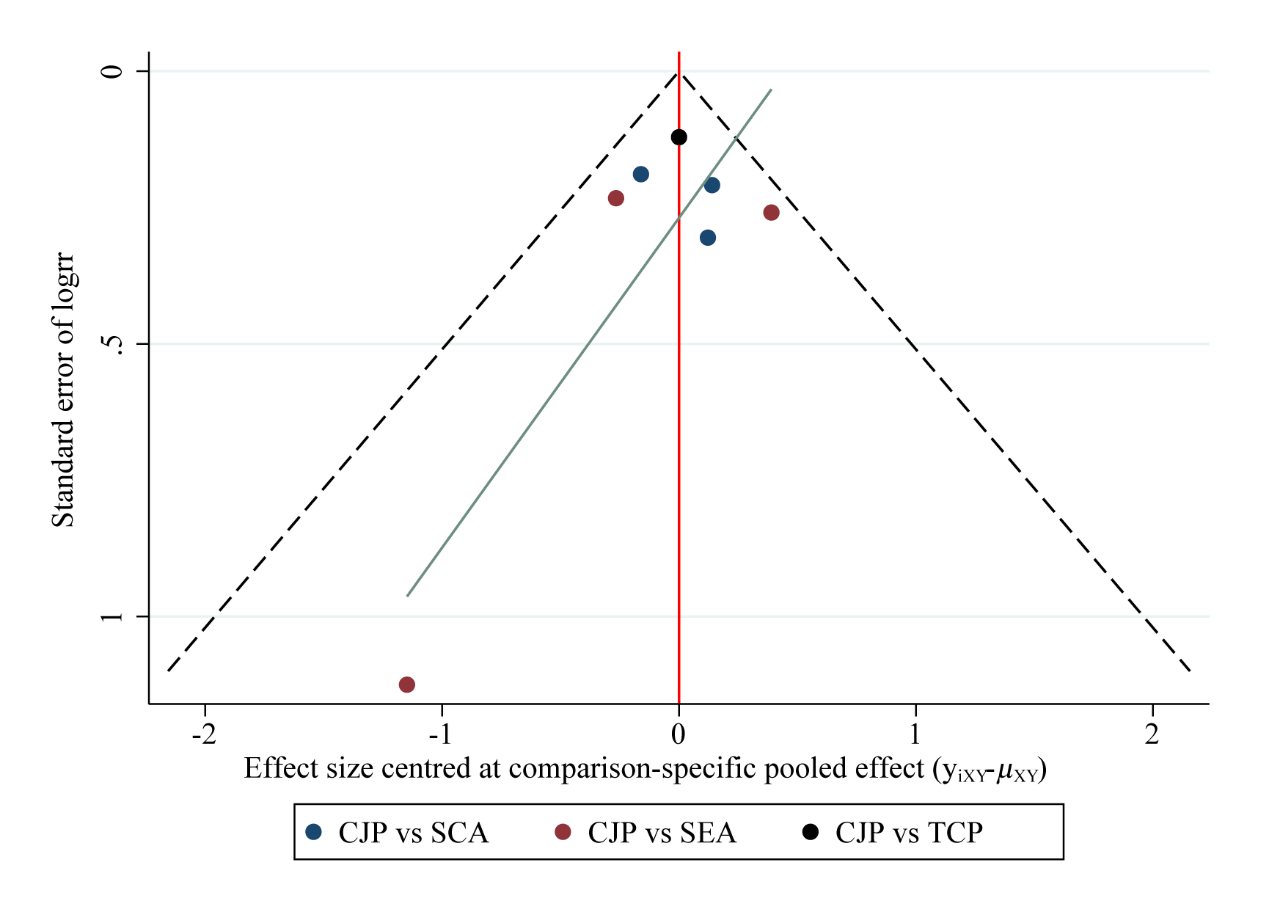
The red line shows the null hypothesis. Each point represents a direct comparison; different colours correspond to different comparisons. The dashed black line represents the 95% confidence interval. The horizontal line represents the regression line; the dark regression line demonstrates that asymmetry is present. CJP, colon J-pouch; SCA, straight colorectal anastomosis; TCP, transverse coloplasty; SEA, side-to-end anastomosis.

**Supplementary Fig. 15A** Network plot for use of antidiarrheal medication at 6 months postoperatively.


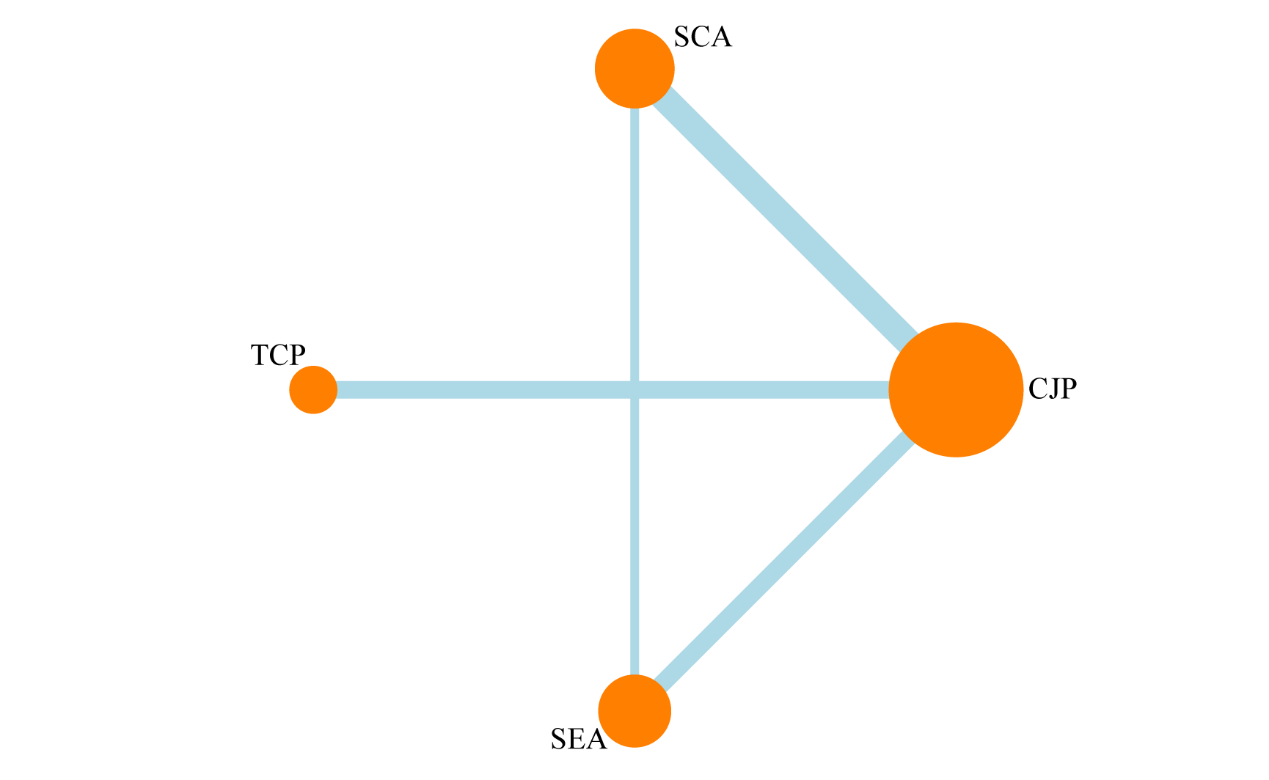
Circles represent interventions and their size is proportional to the number of patients who received the corresponding intervention. Lines represent direct comparisons, and their width is proportional to the number of studies in the corresponding comparison. CJP, colon J-pouch; SCA, straight colorectal anastomosis; TCP, transverse coloplasty; SEA, side-to-end anastomosis.

**Supplementary Table 18A** Relative effects table for use of antidiarrheal medication at 6 months postoperatively.

| **CJP** | 2.95  (0.92, 18.39) | 1.01  (0.17, 6.19) | 0.83  (0.19, 5.79) |
| --- | --- | --- | --- |
|  | **SCA** | 0.35  (0.02, 2.56) | 0.28  (0.04, 1.64) |
|  |  | **TCP** | 0.8  (0.09, 12.61) |
|  |  |  | **SEA** |

Estimates are presented as risk ratio with a 95% confidence interval. Each cell gives the effect of the column-defining intervention relative to the row-defining intervention. The statistically significant results are indicated in bold. CJP, colon J-pouch; SCA, straight colorectal anastomosis; TCP, transverse coloplasty; SEA, side-to-end anastomosis.

**Supplementary Table 18B** Rank probabilities for use of antidiarrheal medication at 6 months postoperatively.

|  | **Rank 1** | **Rank 2** | **Rank 3** | **Rank 4** | **SUCRA** |
| --- | --- | --- | --- | --- | --- |
| **CJP** | 0.192 | 0.494 | 0.303 | 0.011 | 0.622 |
| **SCA** | 0.008 | 0.027 | 0.109 | 0.857 | 0.062 |
| **TCP** | 0.303 | 0.272 | 0.338 | 0.087 | 0.597 |
| **SEA** | 0.497 | 0.208 | 0.250 | 0.045 | 0.718 |

CJP, colon J-pouch; SCA, straight colorectal anastomosis; TCP, transverse coloplasty; SEA, side-to-end anastomosis.

**Supplementary Fig. 15B** Comparison-adjusted funnel plot for use of antidiarrheal medication at 6 months postoperatively.


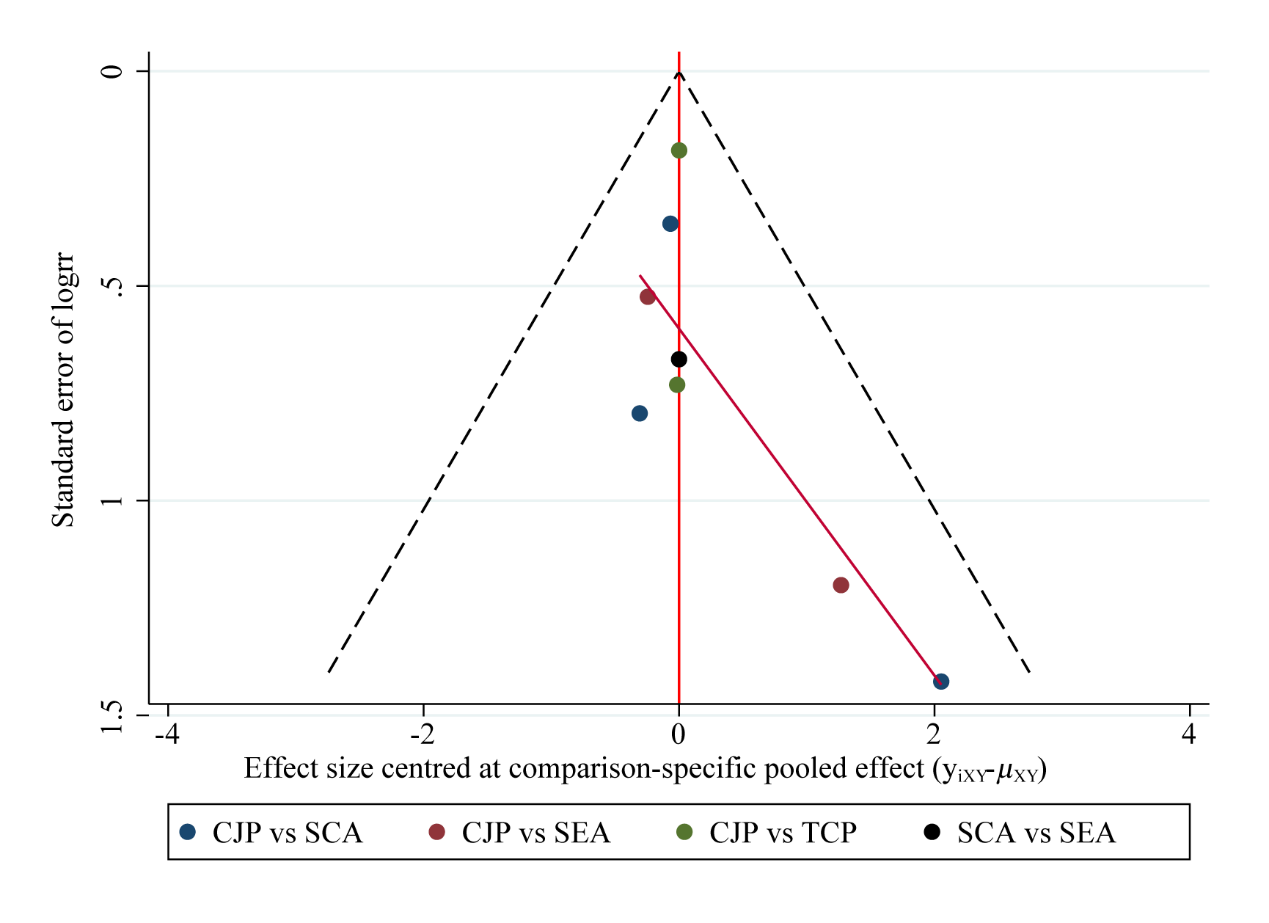
The red line shows the null hypothesis. Each point represents a direct comparison; different colours correspond to different comparisons. The dashed black line represents the 95% confidence interval. The horizontal line represents the regression line; the dark red regression line demonstrates that asymmetry is present. CJP, colon J-pouch; SCA, straight colorectal anastomosis; TCP, transverse coloplasty; SEA, side-to-end anastomosis.

**Supplementary Fig. 16A** Network plot for defecation frequency at 12 months postoperatively.


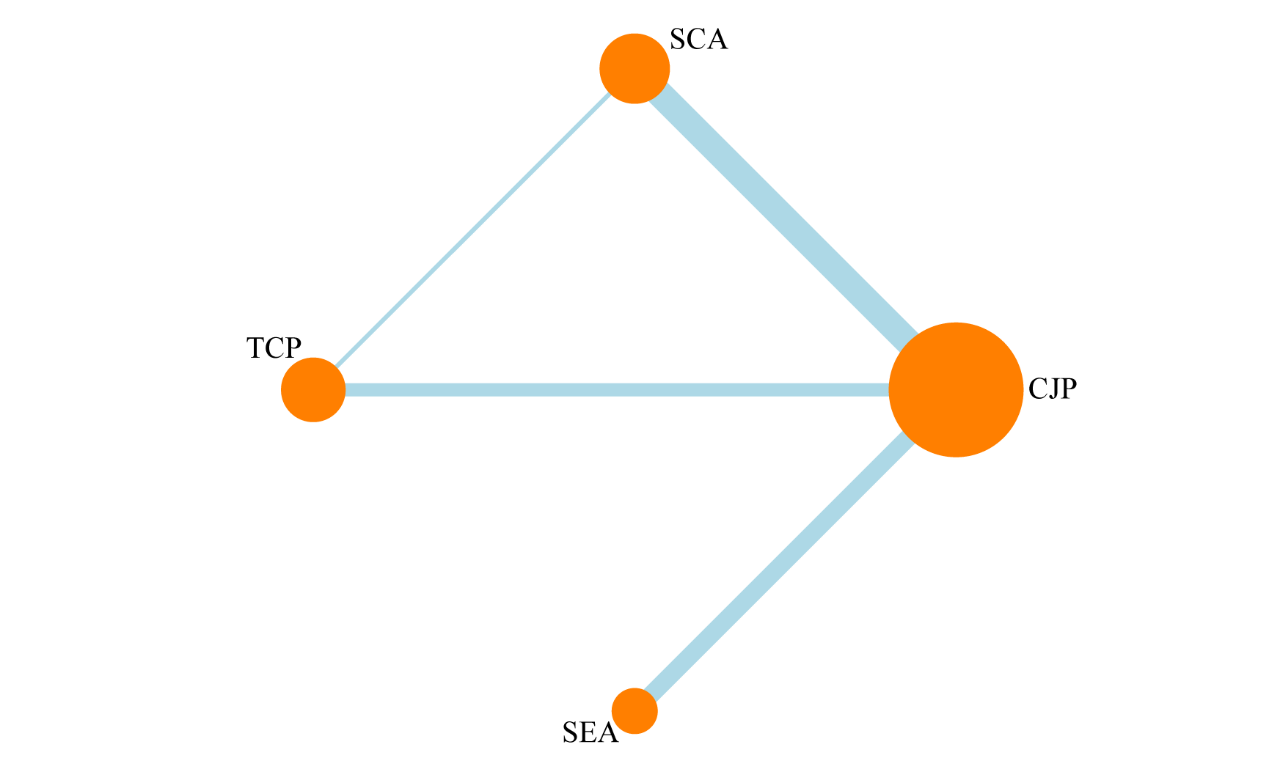


Circles represent interventions and their size is proportional to the number of patients who received the corresponding intervention. Lines represent direct comparisons, and their width is proportional to the number of studies in the corresponding comparison. CJP, colon J-pouch; SCA, straight colorectal anastomosis; TCP, transverse coloplasty; SEA, side-to-end anastomosis.

**Supplementary Table 19B** Rank probabilities for defecation frequency at 12 months postoperatively.

|  | **Rank 1** | **Rank 2** | **Rank 3** | **Rank 4** | **SUCRA** |
| --- | --- | --- | --- | --- | --- |
| **CJP** | 0.377 | 0.476 | 0.147 | 0.001 | 0.743 |
| **SCA** | 0.001 | 0.003 | 0.026 | 0.970 | 0.011 |
| **TCP** | 0.366 | 0.252 | 0.372 | 0.009 | 0.659 |
| **SEA** | 0.256 | 0.269 | 0.455 | 0.020 | 0.587 |

CJP, colon J-pouch; SCA, straight colorectal anastomosis; TCP, transverse coloplasty; SEA, side-to-end anastomosis.

**Supplementary Fig. 16B** Comparison-adjusted funnel plot for defecation frequency at 12 months postoperatively.


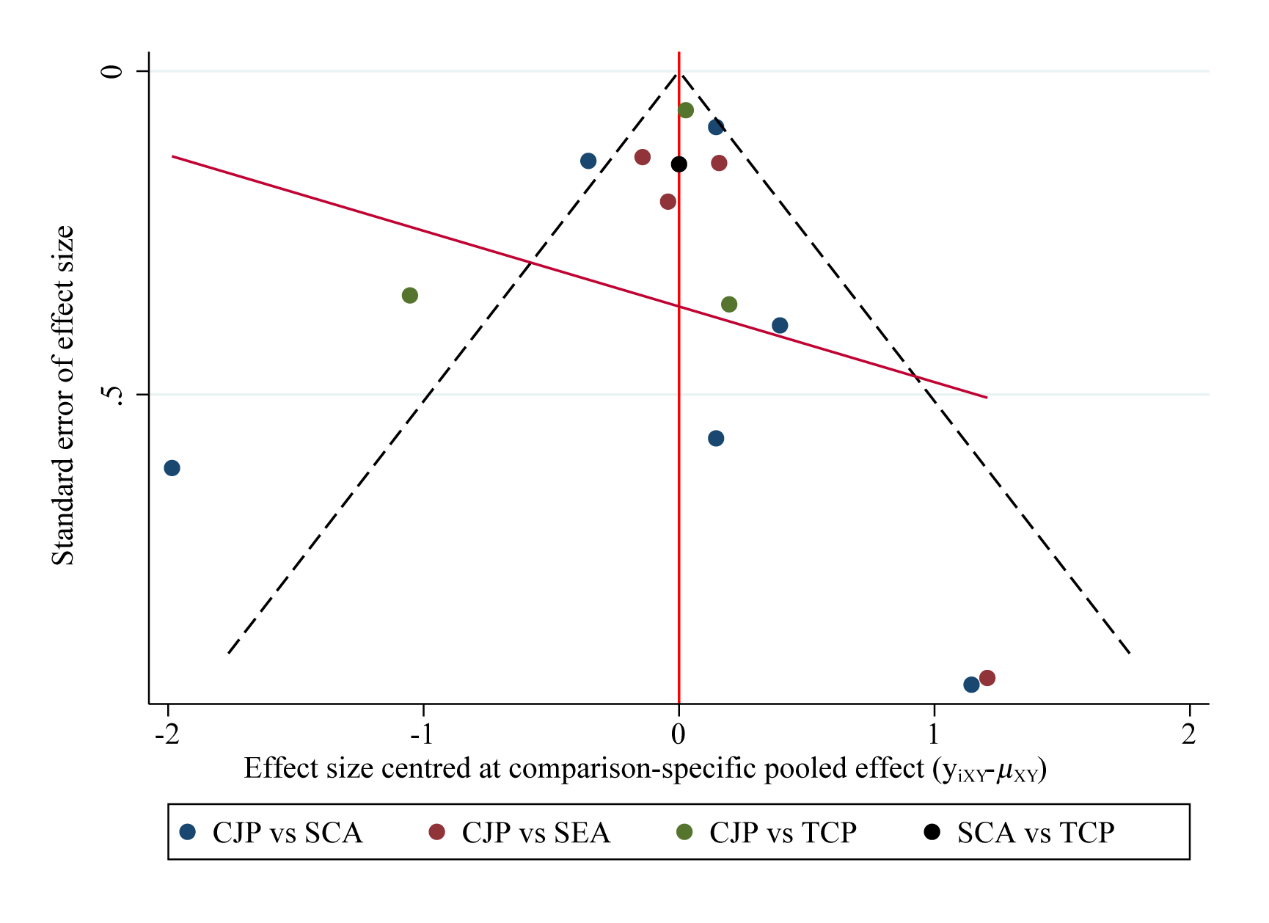


The red line shows the null hypothesis. Each point represents a direct comparison; different colours correspond to different comparisons. The dashed black line represents the 95% confidence interval. The horizontal line represents the regression line; the dark red regression line demonstrates that asymmetry is present. CJP, colon J-pouch; SCA, straight colorectal anastomosis; TCP, transverse coloplasty; SEA, side-to-end anastomosis.

**Supplementary Fig. 17A** Network plot for fecal urgency at 12 months postoperatively.


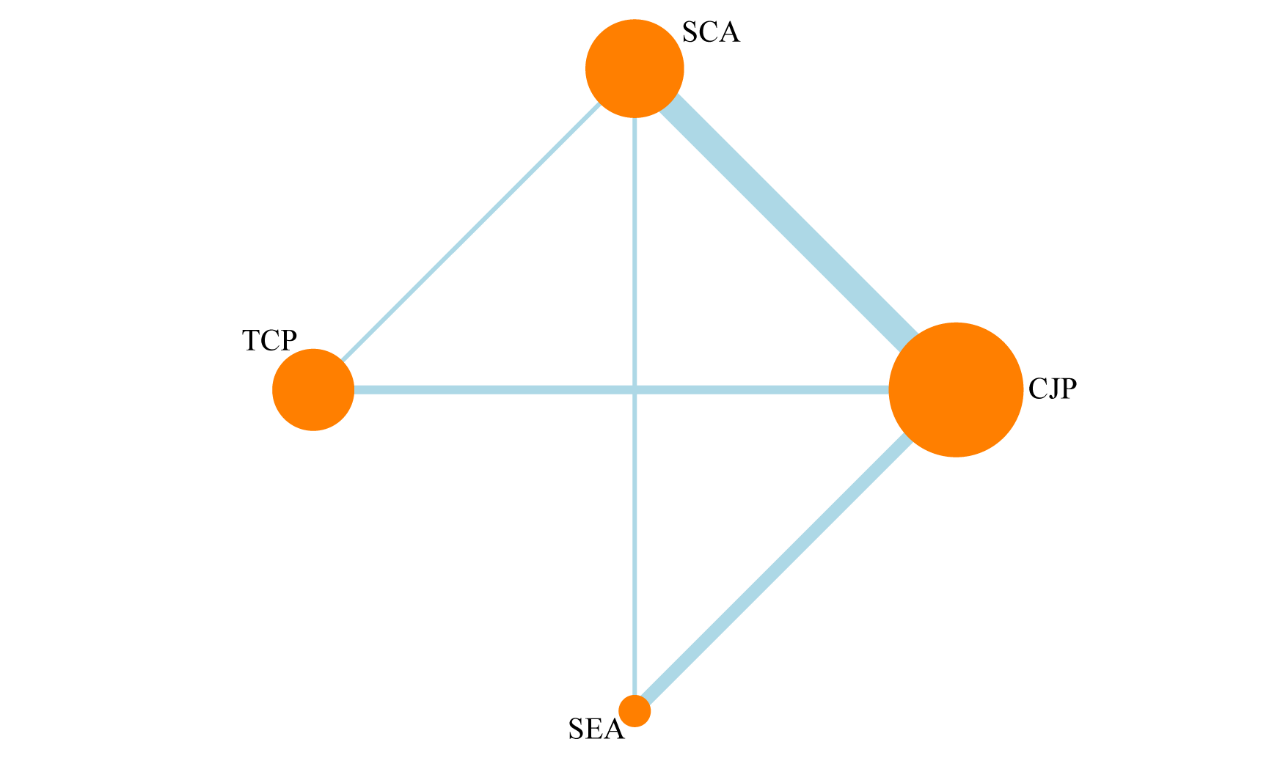


Circles represent interventions and their size is proportional to the number of patients who received the corresponding intervention. Lines represent direct comparisons, and their width is proportional to the number of studies in the corresponding comparison. CJP, colon J-pouch; SCA, straight colorectal anastomosis; TCP, transverse coloplasty; SEA, side-to-end anastomosis.

**Supplementary Table 20A** Relative effects table for fecal urgency at 12 months postoperatively.

| **CJP** | 1.45  (0.98, 3.25) | 1.28  (0.59, 3.10) | 0.91  (0.41, 2.18) |
| --- | --- | --- | --- |
|  | **SCA** | 0.9  (0.31, 1.79) | 0.63  (0.19, 1.47) |
|  |  | **TCP** | 0.71  (0.21, 2.24) |
|  |  |  | **SEA** |

Estimates are presented as risk ratio with a 95% confidence interval. Each cell gives the effect of the column-defining intervention relative to the row-defining intervention. The statistically significant results are indicated in bold. CJP, colon J-pouch; SCA, straight colorectal anastomosis; TCP, transverse coloplasty; SEA, side-to-end anastomosis.

**Supplementary Table 20B** Rank probabilities for fecal urgency at 12 months postoperatively.

|  | **Rank 1** | **Rank 2** | **Rank 3** | **Rank 4** | **SUCRA** |
| --- | --- | --- | --- | --- | --- |
| **CJP** | 0.308 | 0.592 | 0.092 | 0.008 | 0.733 |
| **SCA** | 0.011 | 0.047 | 0.292 | 0.650 | 0.139 |
| **TCP** | 0.093 | 0.141 | 0.504 | 0.263 | 0.354 |
| **SEA** | 0.589 | 0.220 | 0.112 | 0.079 | 0.773 |

CJP, colon J-pouch; SCA, straight colorectal anastomosis; TCP, transverse coloplasty; SEA, side-to-end anastomosis.

**Supplementary Fig. 17B** Comparison-adjusted funnel plot for fecal urgency at 12 months postoperatively.


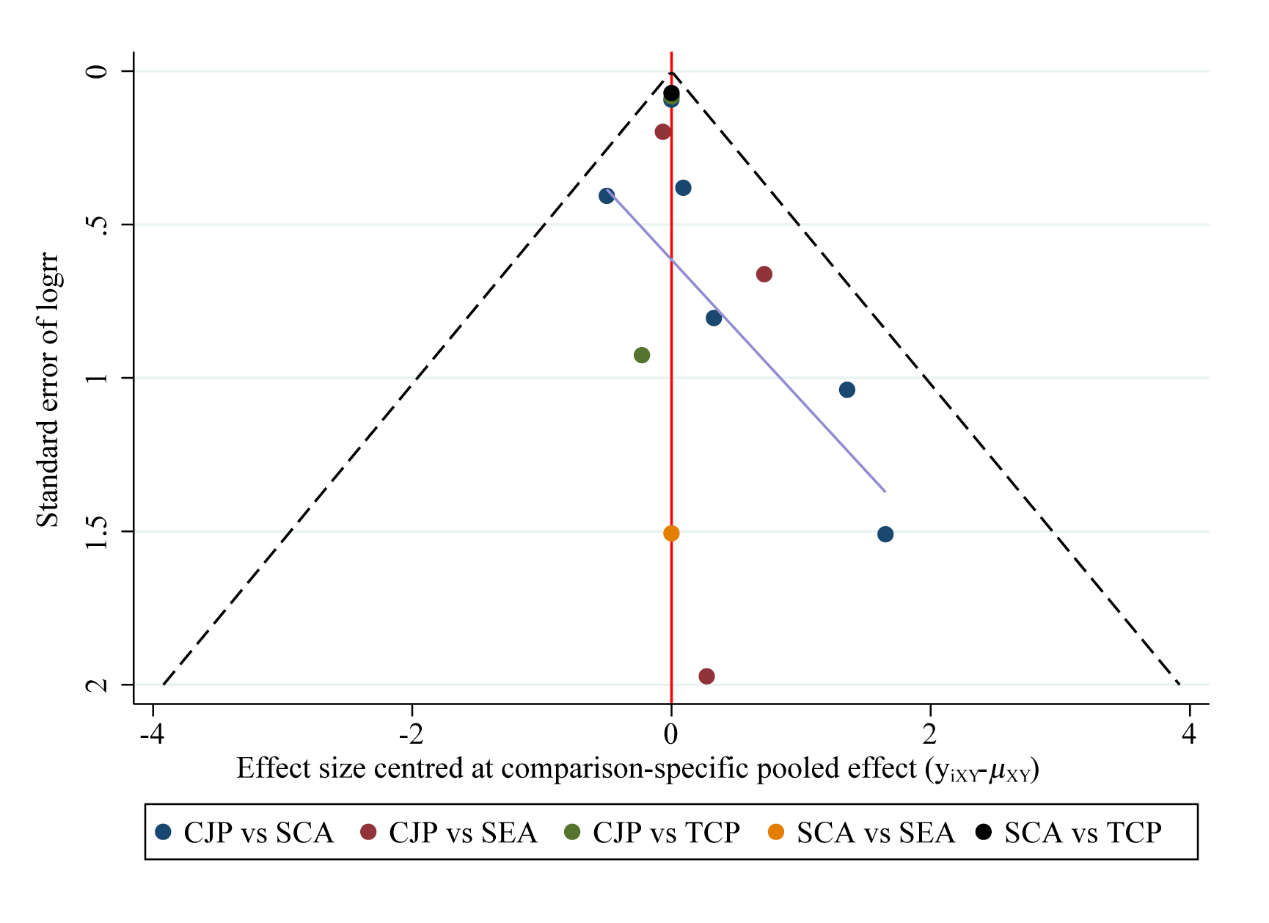
The red line shows the null hypothesis. Each point represents a direct comparison; different colours correspond to different comparisons. The dashed black line represents the 95% confidence interval. The horizontal line represents the regression line; the purple regression line demonstrates that asymmetry is present. CJP, colon J-pouch; SCA, straight colorectal anastomosis; TCP, transverse coloplasty; SEA, side-to-end anastomosis.

**Supplementary Fig. 18A** Network plot for incomplete defecation at 12 months postoperatively.


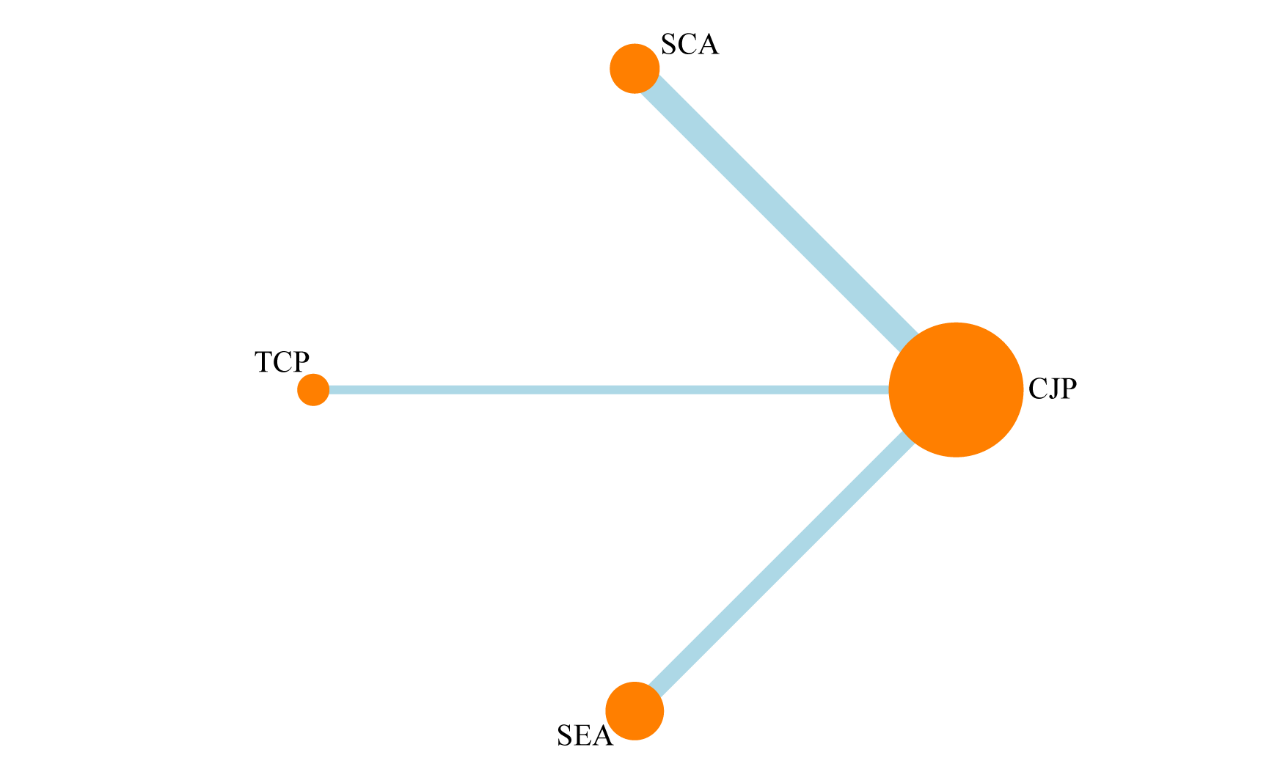


Circles represent interventions and their size is proportional to the number of patients who received the corresponding intervention. Lines represent direct comparisons, and their width is proportional to the number of studies in the corresponding comparison. CJP, colon J-pouch; SCA, straight colorectal anastomosis; TCP, transverse coloplasty; SEA, side-to-end anastomosis.

**Supplementary Table 21A** Relative effects table for incomplete defecation at 12 months postoperatively.

| **CJP** | 0.86  (0.36, 1.82) | 0.83  (0.21, 3.21) | 1.15  (0.43, 3.01) |
| --- | --- | --- | --- |
|  | **SCA** | 0.97  (0.21, 4.94) | 1.33  (0.4, 5.01) |
|  |  | **TCP** | 1.38  (0.26, 7.3) |
|  |  |  | **SEA** |

Estimates are presented as risk ratio with a 95% confidence interval. Each cell gives the effect of the column-defining intervention relative to the row-defining intervention. The statistically significant results are indicated in bold. CJP, colon J-pouch; SCA, straight colorectal anastomosis; TCP, transverse coloplasty; SEA, side-to-end anastomosis.

**Supplementary Table 21B** Rank probabilities for incomplete defecation at 12 months postoperatively.

|  | **Rank 1** | **Rank 2** | **Rank 3** | **Rank 4** | **SUCRA** |
| --- | --- | --- | --- | --- | --- |
| **CJP** | 0.072 | 0.325 | 0.447 | 0.156 | 0.438 |
| **SCA** | 0.346 | 0.324 | 0.195 | 0.135 | 0.627 |
| **TCP** | 0.446 | 0.176 | 0.146 | 0.231 | 0.613 |
| **SEA** | 0.135 | 0.175 | 0.212 | 0.478 | 0.323 |

CJP, colon J-pouch; SCA, straight colorectal anastomosis; TCP, transverse coloplasty; SEA, side-to-end anastomosis.

**Supplementary Fig. 18B** Comparison-adjusted funnel plot for incomplete defecation at 12 months postoperatively.


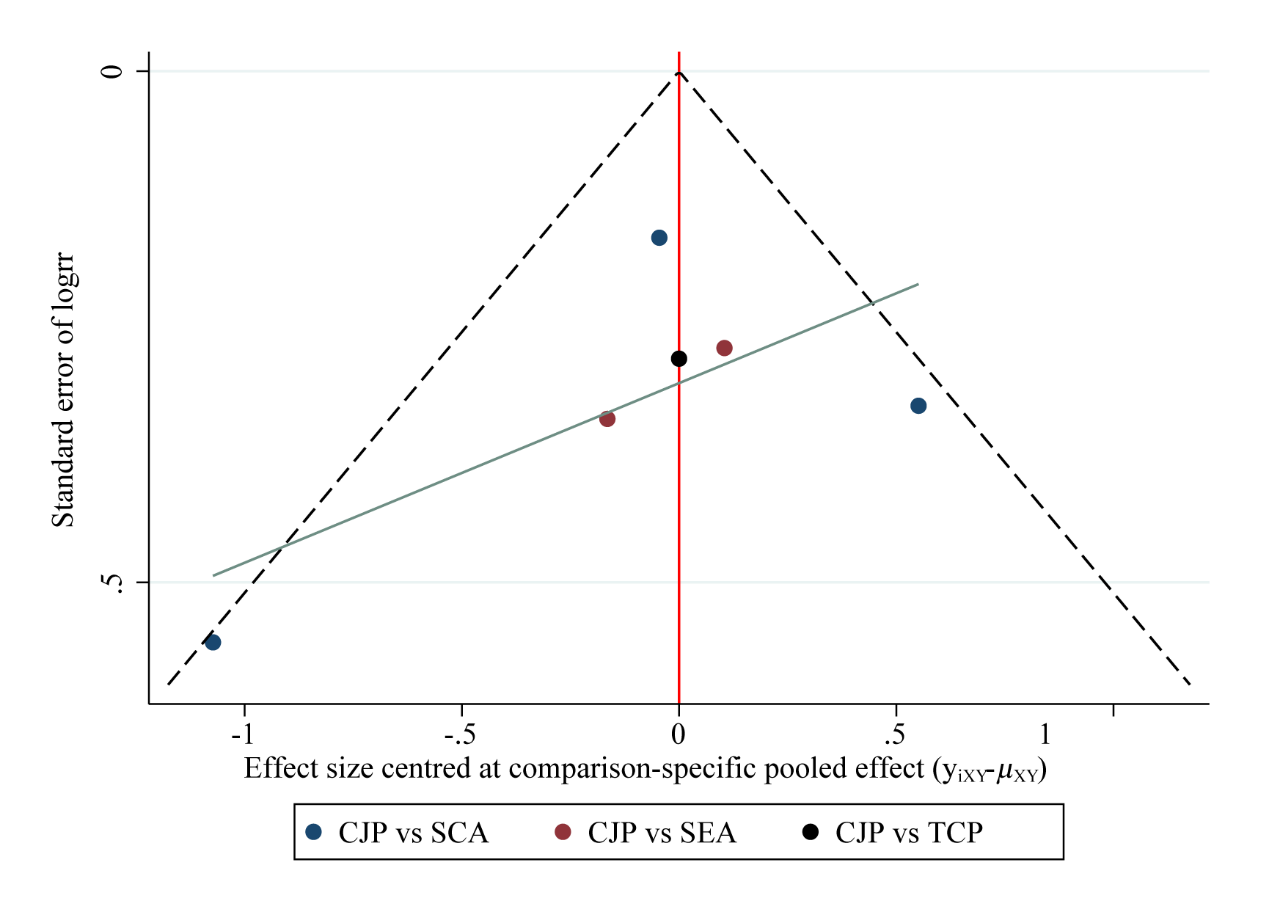
The red line shows the null hypothesis. Each point represents a direct comparison; different colours correspond to different comparisons. The dashed black line represents the 95% confidence interval. The horizontal line represents the regression line; the dark regression line demonstrates that asymmetry is present. CJP, colon J-pouch; SCA, straight colorectal anastomosis; TCP, transverse coloplasty; SEA, side-to-end anastomosis.

**Supplementary Fig. 19A** Network plot for use of antidiarrheal medication at 12 months postoperatively.


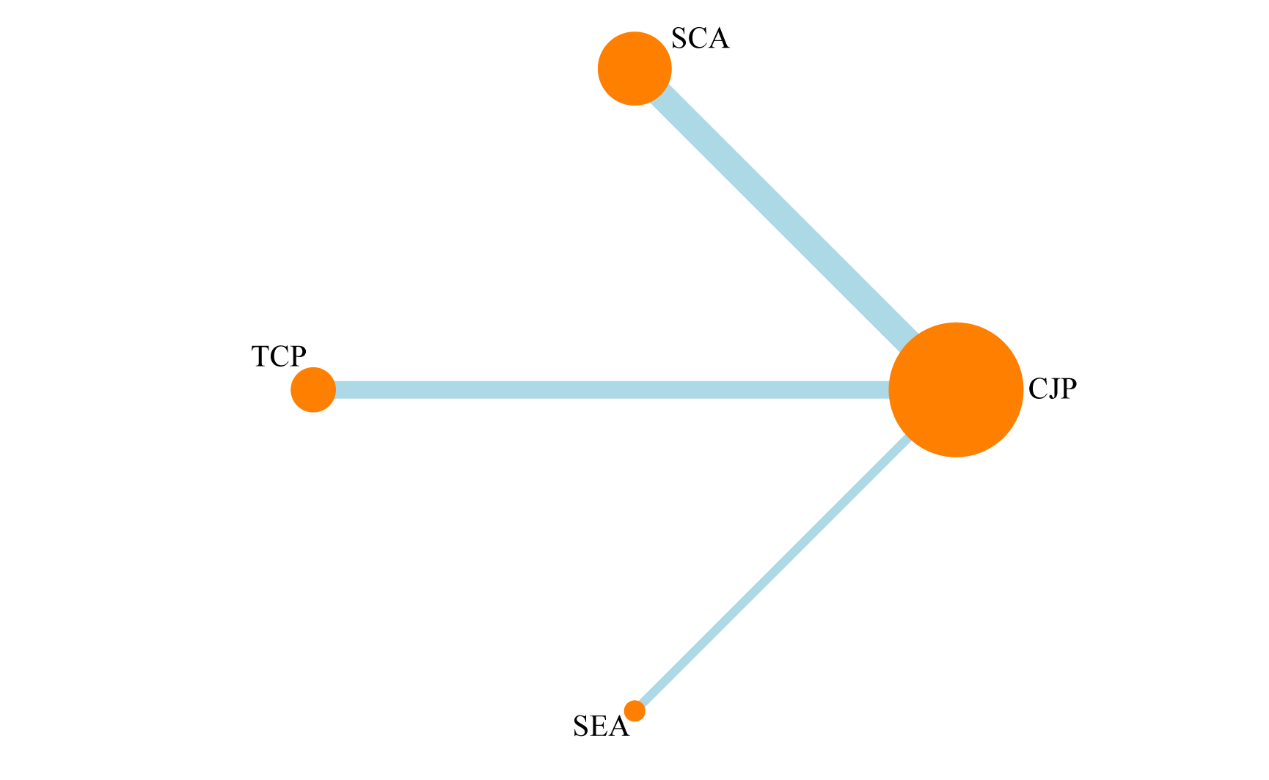


Circles represent interventions and their size is proportional to the number of patients who received the corresponding intervention. Lines represent direct comparisons, and their width is proportional to the number of studies in the corresponding comparison. CJP, colon J-pouch; SCA, straight colorectal anastomosis; TCP, transverse coloplasty; SEA, side-to-end anastomosis.

**Supplementary Table 22A** Relative effects table for use of antidiarrheal medication at 12 months postoperatively.

| **CJP** | 5.83  (0.85, 81.65) | 0.94  (0.07, 12.18) | 0.70  (0.02, 24.55) |
| --- | --- | --- | --- |
|  | **SCA** | 0.16  (0.01, 3.66) | 0.12  (0.01, 5.83) |
|  |  | **TCP** | 0.73  (0.01, 59.59) |
|  |  |  | **SEA** |

Estimates are presented as risk ratio with a 95% confidence interval. Each cell gives the effect of the column-defining intervention relative to the row-defining intervention. The statistically significant results are indicated in bold. CJP, colon J-pouch; SCA, straight colorectal anastomosis; TCP, transverse coloplasty; SEA, side-to-end anastomosis.

**Supplementary Table 22B** Rank probabilities for use of antidiarrheal medication at 12 months postoperatively.

|  | **Rank 1** | **Rank 2** | **Rank 3** | **Rank 4** | **SUCRA** |
| --- | --- | --- | --- | --- | --- |
| **CJP** | 0.188 | 0.486 | 0.316 | 0.010 | 0.618 |
| **SCA** | 0.012 | 0.035 | 0.157 | 0.796 | 0.088 |
| **TCP** | 0.320 | 0.295 | 0.297 | 0.088 | 0.616 |
| **SEA** | 0.480 | 0.184 | 0.230 | 0.106 | 0.679 |

CJP, colon J-pouch; SCA, straight colorectal anastomosis; TCP, transverse coloplasty; SEA, side-to-end anastomosis.

**Supplementary Fig. 19B** Comparison-adjusted funnel plot for use of antidiarrheal medication at 12 months postoperatively.


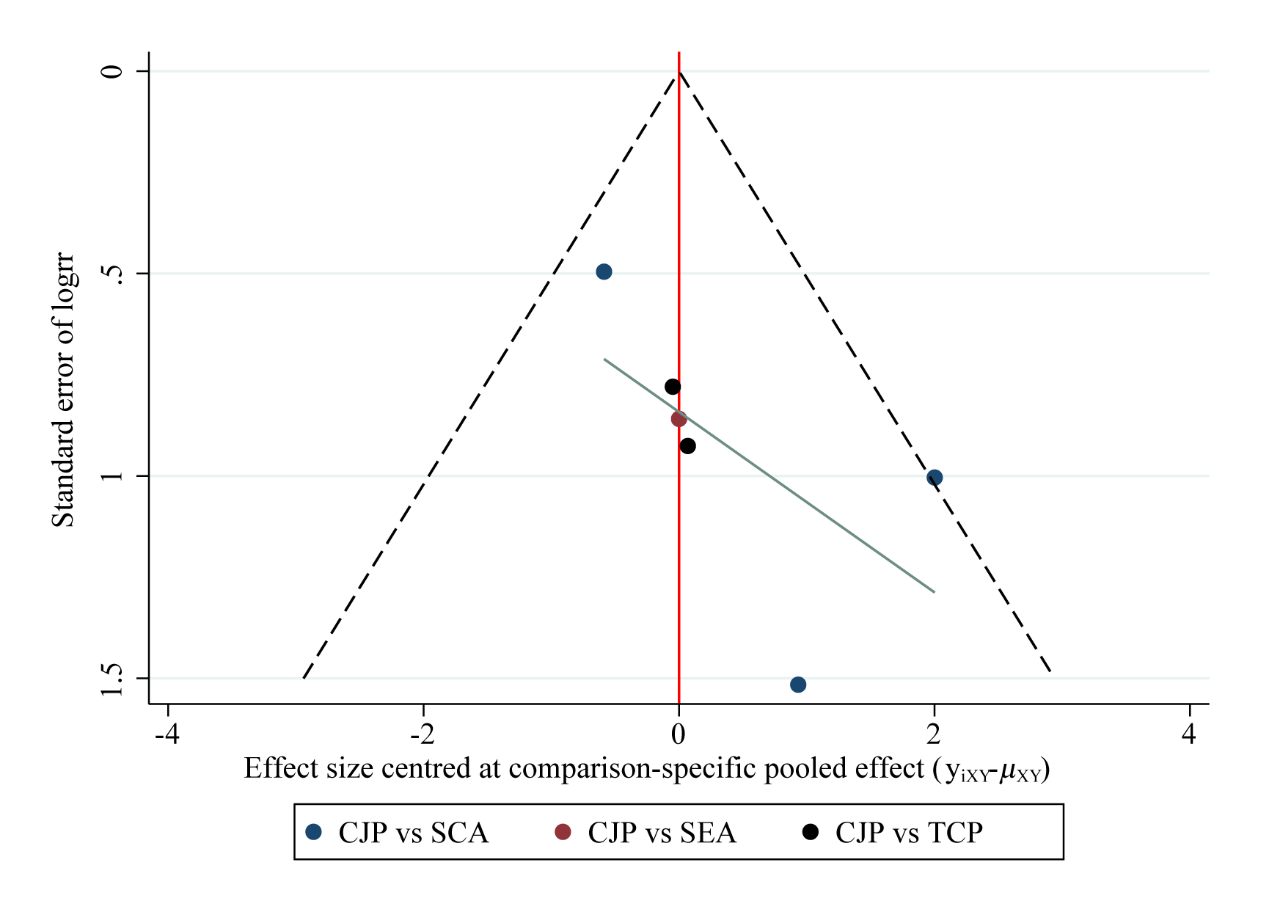


The red line shows the null hypothesis. Each point represents a direct comparison; different colours correspond to different comparisons. The dashed black line represents the 95% confidence interval. The horizontal line represents the regression line; the dark regression line demonstrates that asymmetry is present. CJP, colon J-pouch; SCA, straight colorectal anastomosis; TCP, transverse coloplasty; SEA, side-to-end anastomosis.

**Supplementary Fig. 20A** Network plot for defecation frequency at 24 months postoperatively.


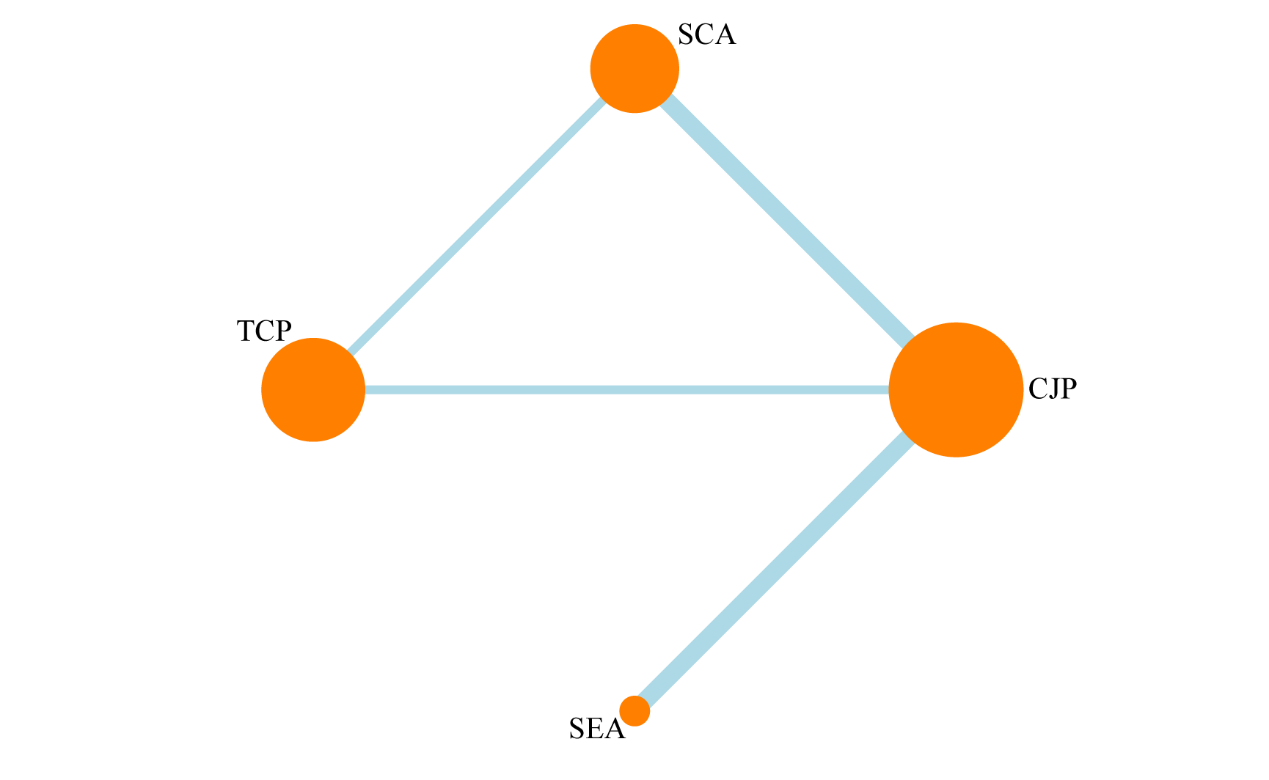


Circles represent interventions and their size is proportional to the number of patients who received the corresponding intervention. Lines represent direct comparisons, and their width is proportional to the number of studies in the corresponding comparison. CJP, colon J-pouch; SCA, straight colorectal anastomosis; TCP, transverse coloplasty; SEA, side-to-end anastomosis.

**Supplementary Table 23B** Rank probabilities for defecation frequency at 24 months postoperatively.

|  | **Rank 1** | **Rank 2** | **Rank 3** | **Rank 4** | **SUCRA** |
| --- | --- | --- | --- | --- | --- |
| **CJP** | 0.317 | 0.565 | 0.099 | 0.019 | 0.727 |
| **SCA** | 0.048 | 0.094 | 0.505 | 0.352 | 0.279 |
| **TCP** | 0.065 | 0.079 | 0.290 | 0.566 | 0.214 |
| **SEA** | 0.570 | 0.262 | 0.106 | 0.063 | 0.779 |

CJP, colon J-pouch; SCA, straight colorectal anastomosis; TCP, transverse coloplasty; SEA, side-to-end anastomosis.

**Supplementary Fig. 20B** Comparison-adjusted funnel plot for defecation frequency at 24 months postoperatively.


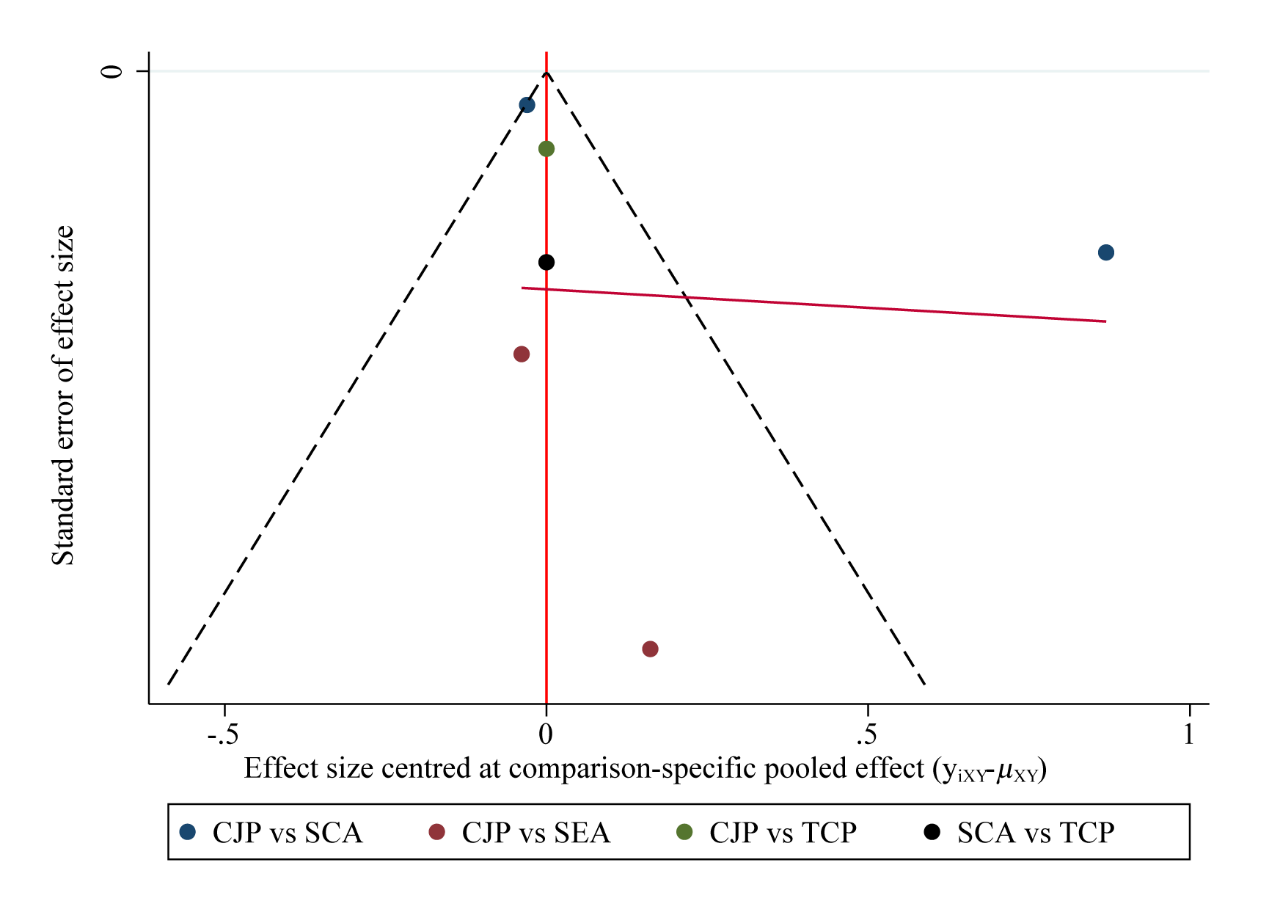


The red line shows the null hypothesis. Each point represents a direct comparison; different colours correspond to different comparisons. The dashed black line represents the 95% confidence interval. The horizontal line represents the regression line; the dark red regression line demonstrates that asymmetry is present. CJP, colon J-pouch; SCA, straight colorectal anastomosis; TCP, transverse coloplasty; SEA, side-to-end anastomosis.

**Supplementary Fig. 21A** Network plot for use of antidiarrheal medication at 24 months postoperatively.
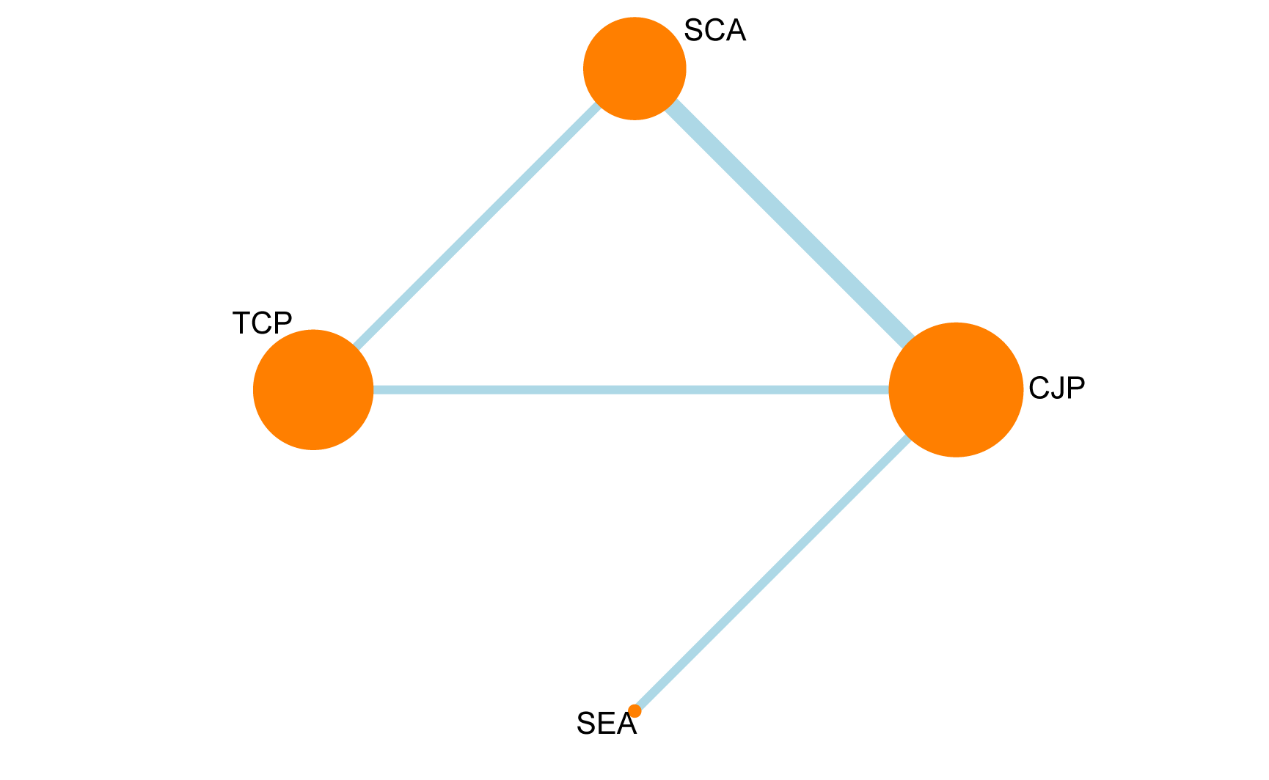


Circles represent interventions and their size is proportional to the number of patients who received the corresponding intervention. Lines represent direct comparisons, and their width is proportional to the number of studies in the corresponding comparison. CJP, colon J-pouch; SCA, straight colorectal anastomosis; TCP, transverse coloplasty; SEA, side-to-end anastomosis.

**Supplementary Table 24A** Relative effects table for use of antidiarrheal medication at 24 months postoperatively.

| **CJP** | 1.40  (0.77, 2.57) | 1.60  (0.91, 2.91) | 0.50  (0.02, 5.70) |
| --- | --- | --- | --- |
|  | **SCA** | 1.14  (0.63, 2.14) | 0.36  (0.02, 4.35) |
|  |  | **TCP** | 0.32  (0.01, 3.8) |
|  |  |  | **SEA** |

Estimates are presented as risk ratio with a 95% confidence interval. Each cell gives the effect of the column-defining intervention relative to the row-defining intervention. The statistically significant results are indicated in bold. CJP, colon J-pouch; SCA, straight colorectal anastomosis; TCP, transverse coloplasty; SEA, side-to-end anastomosis.

**Supplementary Table 24B** Rank probabilities for use of antidiarrheal medication at 24 months postoperatively.

|  | **Rank 1** | **Rank 2** | **Rank 3** | **Rank 4** | **SUCRA** |
| --- | --- | --- | --- | --- | --- |
| **CJP** | 0.244 | 0.650 | 0.093 | 0.013 | 0.708 |
| **SCA** | 0.039 | 0.192 | 0.525 | 0.244 | 0.342 |
| **TCP** | 0.012 | 0.076 | 0.331 | 0.580 | 0.173 |
| **SEA** | 0.705 | 0.082 | 0.051 | 0.163 | 0.776 |

CJP, colon J-pouch; SCA, straight colorectal anastomosis; TCP, transverse coloplasty; SEA, side-to-end anastomosis.

**Supplementary Fig. 21B** Comparison-adjusted funnel plot for use of antidiarrheal medication at 24 months postoperatively.
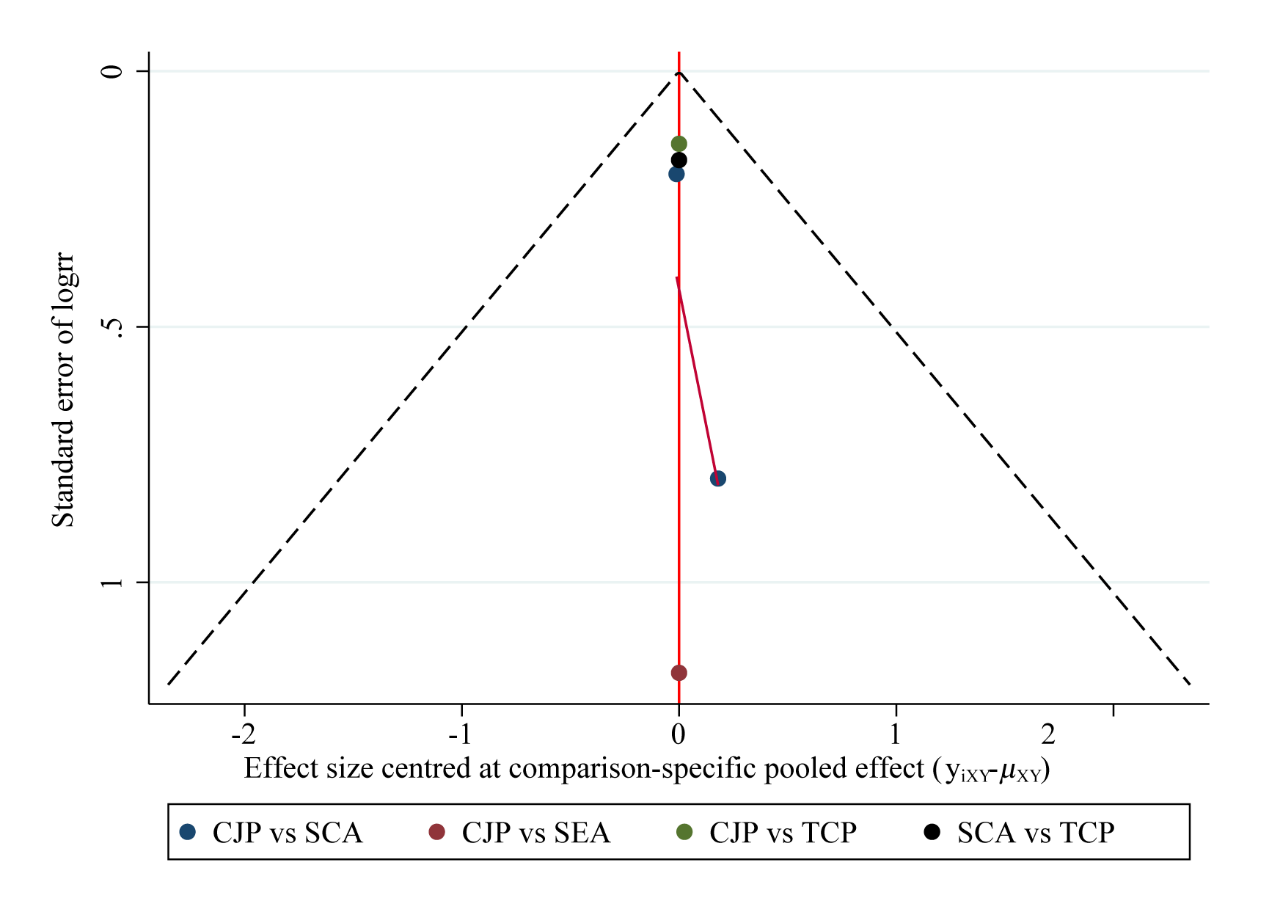


The red line shows the null hypothesis. Each point represents a direct comparison; different colours correspond to different comparisons. The dashed black line represents the 95% confidence interval. The horizontal line represents the regression line; the dark regression line demonstrates that asymmetry is present. CJP, colon J-pouch; SCA, straight colorectal anastomosis; TCP, transverse coloplasty; SEA, side-to-end anastomosis.

**Supplementary Table 25A** Relative effects table for postoperative anastomotic leakage in the sensitivity analysis.

| **CJP** | 1.64  (0.69, 4.14) | 2.52  (0.84, 9.45) | 0.48  (0.16, 1.16) |
| --- | --- | --- | --- |
|  | **SCA** | 1.53  (0.43, 6.55) | **0.29**  **(0.08, 0.83)** |
|  |  | **TCP** | **0.19**  **(0.03, 0.73)** |
|  |  |  | **SEA** |

Estimates are presented as risk ratio with a 95% confidence interval. Each cell gives the effect of the column-defining intervention relative to the row-defining intervention. The statistically significant results are indicated in bold. CJP, colon J-pouch; SCA, straight colorectal anastomosis; TCP, transverse coloplasty; SEA, side-to-end anastomosis.

**Supplementary Table 25B** Rank probabilities for postoperative anastomotic leakage in the sensitivity analysis.

|  | **Rank 1** | **Rank 2** | **Rank 3** | **Rank 4** | **SUCRA** |
| --- | --- | --- | --- | --- | --- |
| **CJP** | 0.043 | 0.816 | 0.132 | 0.009 | 0.631 |
| **SCA** | 0.008 | 0.100 | 0.657 | 0.235 | 0.293 |
| **TCP** | 0.006 | 0.038 | 0.202 | 0.754 | 0.099 |
| **SEA** | 0.943 | 0.046 | 0.009 | 0.002 | 0.977 |

CJP, colon J-pouch; SCA, straight colorectal anastomosis; TCP, transverse coloplasty; SEA, side-to-end anastomosis.
